# Access to cationic polyhedral carboranes via dynamic cage surgery with *N*-heterocyclic carbenes

**DOI:** 10.1038/s41467-021-25277-0

**Published:** 2021-08-17

**Authors:** Jan Vrána, Josef Holub, Maksim A. Samsonov, Zdeňka Růžičková, Josef Cvačka, Michael L. McKee, Jindřich Fanfrlík, Drahomír Hnyk, Aleš Růžička

**Affiliations:** 1 Department of General and Inorganic Chemistry, Faculty of Chemical Technology, University of Pardubice, Pardubice, Czech Republic; 2 Institute of Inorganic Chemistry, Czech Academy of Sciences, Řež, Czech Republic; 3 Institute of Organic Chemistry and Biochemistry of the Czech Academy of Sciences, Prague, Czech Republic; 4 Department of Chemistry and Biochemistry, Auburn University, Auburn, AL USA

**Keywords:** Chemical bonding, Ligands, Chemical bonding

## Abstract

Polyhedral boranes and heteroboranes appear almost exclusively as neutral or anionic species, while the cationic ones are protonated at exoskeletal heteroatoms or they are instable. Here we report the reactivity of 10-vertex *closo*-dicarbadecaboranes with one or two equivalents of *N*-heterocyclic carbene to 10-vertex *nido* mono- and/or bis-carbene adducts, respectively. These complexes easily undergo a reaction with HCl to give cages of stable and water soluble 10-vertex *nido*-type cations with protonation in the form of a BHB bridge or 10-vertex *closo*-type cations containing one carbene ligand when originating from *closo*-1,10-dicarbadecaborane. The reaction of a 10-vertex *nido* mono-carbene adduct with phosphorus trichloride gives *nido*-11-vertex 2-phospha-7,8-dicarbaundecaborane, which undergoes an oxidation of the phosphorus atom to P = O, while the product of a bis-carbene adduct reaction is best described as a distorted C_2_B_6_H_8_ fragment bridged by the (BH)_2_PCl_2_^+^ moiety.

## Abstract (web summary)

In comparison to their neutral or anionic counterparts, examples of cationic boron clusters remain scarce. Here, the authors prepare a variety of cationic polyhedral boranes by reacting *closo*-10-vertex carboranes with N-heterocyclic carbenes; the resulting open-cage cationic *nido*- *arachno*- or *closo*- derivatives are water soluble, which may enable unprecedented applications.

## Introduction

In the hundred-year-history of boron hydrides and their successors (polyhedral boranes, carboranes and other heteroboranes) and derivatives, the number, molecular shape, reactivity and applications of these species have become enormous, thus establishing a self-consistent field of chemistry^1–3^. Examples in synthetic organic chemistry and catalysis include the early use of diborane in organic chemistry as a reducing agent^4^; ionic *closo*-carboranes as weakly-coordinating anions, transfer agents^5^ or recently as a supporting scaffold for the stabilisation of low-valent main-group elements by silylenes^4–10^; and low-coordinate boron species for the one-pot reduction of dinitrogen to ammonium chloride^11–13^. Especially the last case, based on Robinson’s idea, further developed by Braunschweig, of the use of supporting *N*-heterocyclic carbene (NHC) ligands^14–19^, could open avenues for synthetic applications. There are also a number of applications in multidisciplinary areas such as energy storage and conversion^20^, materials science^21^, nuclear-waste treatment and medicine^22,23^, which confirms the broad scope of this area of chemistry.

Some of the most successful tools for the description and prediction of the structure of these mostly deltahedral species are structural relationships and the related Wade–Mingos rules^24–28^, which are based on the electron count around the cluster or the number of skeletal electron pairs (SEPs). In general, the species with the same number of SEPs (*n* + 1) are related if one or two vertices are removed from the neutral or anionic parent *closo* cluster−e.g. *closo* (*n* vertices), *nido* (*n* − 1 vertices), *arachno* (*n* − 2 vertices) and *hypho* (*n* − 3 vertices). In other words, for a series of clusters with the same number of vertices (*n*), each step requires one extra SEP, hence *n* + 1 for *closo* up to *n* + 4 for *hypho*. This is only achievable by an increase in the negative charge or the addition of bridging hydrogen atoms. At the same time, the number of the SEPs for monocationic clusters is likely to be different, *n* *+* 0.5 for *closo* up to *n* + 3.5 for *hypho*, but the cationic clusters are still elusive, except for *closo*-B_12_(3-methylbutyl)_12 _^+ ^radical cation published very recently by Spokoyny^29^. There is a big number of cationic boranes, however, the positive charge is predominantly located on a functional group connected to the parent borane (Me_2_S^30^, phosphonium^31^ and ammonium^32^), not in the borane cage itself.

Taking these considerations into account, one could expect that the addition of a strong σ-donor such as NHCs would increase the electron density on those traditionally taken electron-deficient skeletons and stabilise them like in the many cases of low-valent or cationic main-group elements, described specifically for boron compounds by Braunschweig^11–13^. Surprisingly enough, the number of studies dealing with the reactivity of polyhedral boranes and heteroboranes with NHCs is limited to 12-vertex *closo*-heteroboranes (*o*-carborane – 1,2-dicarbadodecaborane(12) and 1-thiadodecaborane(12))^33–35^ and bridged/strained 13-vertex heteroboranes^36–40^. The reactions of *o*-carborane and Xie’s 13-vertex dicarbaboranes mostly lead to deprotonated or deboronated species with one less vertex; in the reactions of thiaborane, however, also cage distortion and the rearrangement of one hydrogen atom from terminal to bridging position have been observed. Such a transformation should formally lead to a *nido*-arrangement, but the count of SEPs varies depending on the nature of the bond between the borane cluster and carbene. In addition, the *o*-carborane reacts with two equivalents of Os_3_(CO)_10_(NCMe)_2_ upon opening the carborane cage by C–B and B–B bonds cleavage^41^, while the reduction of main-group element compounds containing the redox non-innocent bis(silylenyl)-*o*-carboranyl ligand led to the breaking of C–C bond upon formation of radical anions^6–10^.

Based on the fact that decaborane(14) is the most prominent starting compound in the chemistry of boranes and heteroboranes, we started our exploration of the reactivity of NHCs with compounds in ten-vertex series in order to find access to elusive compounds and structural patterns.

In this work, we report the reactions of one of the most sterically demanding NHCs with *closo*-dicarbadecaborane series, as smaller congeners of the most frequently used 12-vertex *closo* compounds, which yields ten-vertex *nido* mono- and/or bis-carbene adducts, respectively. Upon reaction with HCl, thermally stable and water-soluble positively charged cage derivatives are obtained.

## Results and discussion

**Fig. 1:**
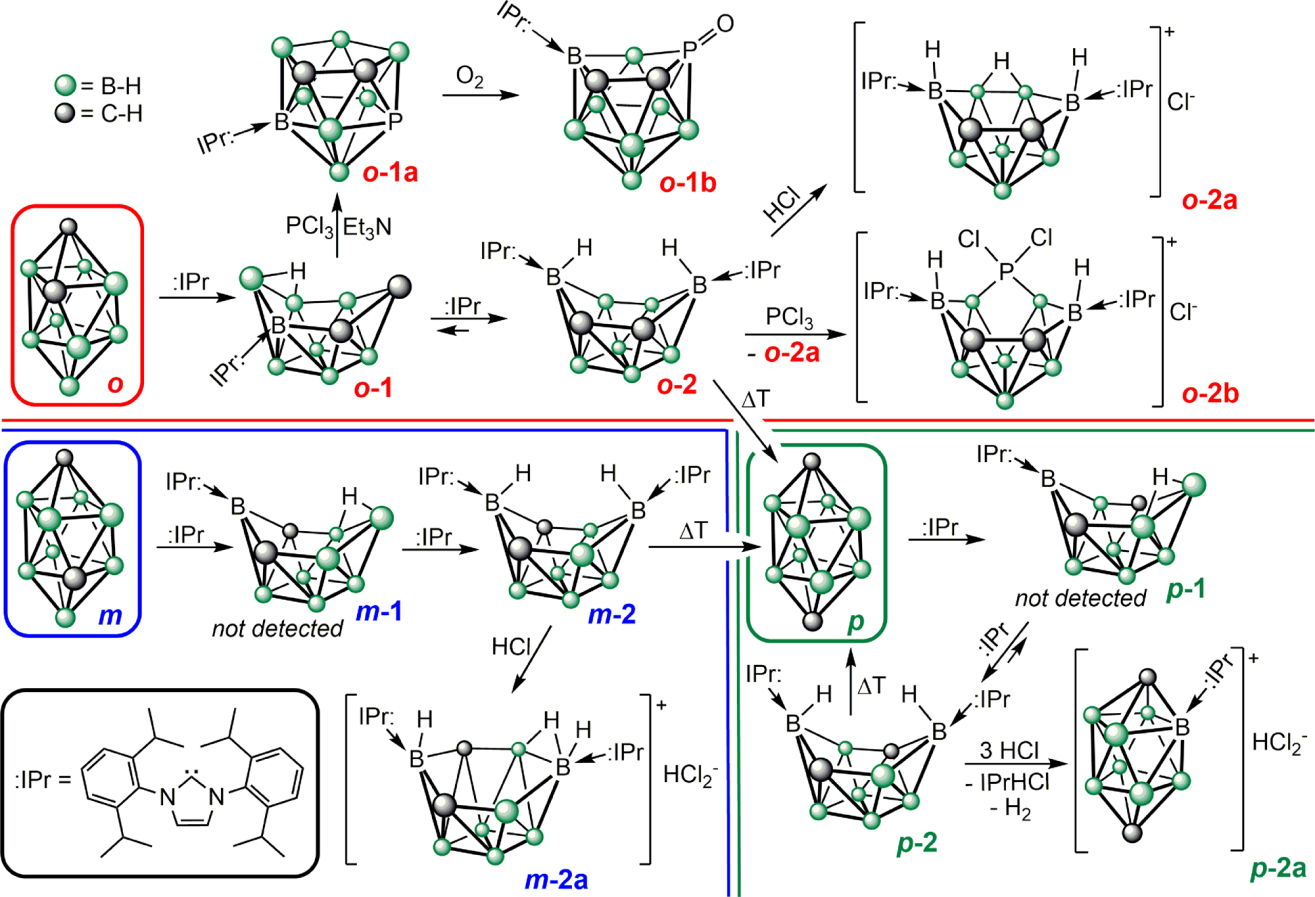
Reactivity of 10-vertex closo-dicarbadecaboranes. All the ten-vertex carboranes show different reactivity towards the carbene and subsequently hydrogen chloride or phosphorus trichloride. *closo*-1,2-dicarbadecaborane is referred to as *ortho* (***o***, red trace); *closo*-1,6-dicarbadecaborane is referred to as *meta* (***m***, blue race), *closo*-1,10-dicarbadecaborane is referred to as para (***p***, green trace). .

**Fig. 2:**
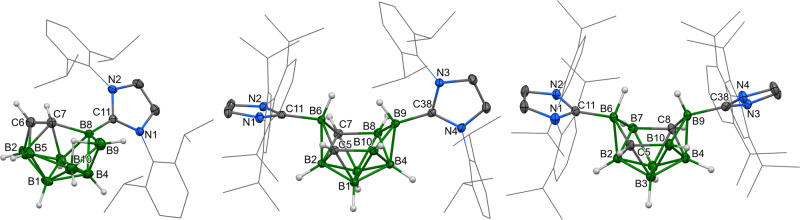
The molecular structures of *o*-1 (left), *m*-2 (centre) and *p*-2 (right). ORTEP-type plots, 40% probability level. The 2,6-diisopropylphenyl groups are displayed as wireframes for clarity.

Three particular pathways (Fig. 1) of selected NHC (:IPr, 1,3-(2,6-*i*Pr_2_C_6_H_3_)-imidazole-2-ylidene) reactivity with three different ten-vertex *closo*-dicarbadecaboranes (*closo*-1,2-dicarbadecaborane, in the trivial nomenclature referred to as *ortho* (***o*** – red trace); *closo*-1,6-dicarbadecaborane – *meta* (***m*** – blue trace); and *closo*-1,10-dicarbadecaborane – *para* (***p*** – green trace)) have been investigated. First, ***o*** reacts vigorously with one equivalent of :IPr, producing *nido* ***o***-**1**, which is in accordance with the reactivity of 12-vertex heteroboranes *closo*-C_2_B_10_H_12_ or *closo*-12-X-1-SB_11_H_10_ (R = H, I)^33–35^. The ^11^B NMR spectrum exhibits eight different signals of equal intensity. Despite the neutral charge of ***o*-1**, its pattern is remarkably similar to that of its structural analogue, *nido*-5,6-C_2_B_8_H_11_^-^^42^. The solid-state structure of ***o***-**1** has been determined by scXRD methods (Fig. 2). ***o***-**1** crystallises in the acentric group *I*4_1_*cd* with distorted decaborane(14)-like structure containing one bridging hydrogen located between B9 and B10 atoms. The separations of C11 atom from carbene and B8 [1.583(3) Å] are close to the values found for the reported adducts of a carbene and boranes, carboranes or thiaboranes, respectively^33–35^. Interestingly, the distances from the upper vertice C6 to C7 [1.474(4) Å] and B5 [1.503(4) Å] and from the upper vertice B9 to B8 [1.672(4) Å] are significantly shorter than the separations of the respective elements found in related compounds. On the other hand, the B5–B10 bond is much longer [1.969(4) Å] than the rest of such B–B bonds and is reminiscent of the upper-rim B–B bonds in pseudo-*nido* thiaborane–carbene adducts^35^.

Remarkably, ***o*** (***o*-1**) reacts solidly with two (one) equivalents of :IPr (Fig. 1), producing borane ***o*-2**. This is in contrast with the published reactivity of 12-vertex heteroboranes, which undergo deprotonation or boron-atom extrusion with the addition of the second equivalent of the carbene^33–35^. The addition of an overstoichiometric amount of the carbene does not yield any further product. On the contrary, the reaction between ***m*** or ***p*** and :IPr proceeded directly to furnish ***m*-2** or ***p*-2** regardless of the stoichiometry used. There is no evidence of the formation of ***m*-1** or ***p*-1** (Fig. 1) as intermediates according to the NMR spectra measured immediately after the mixing of the parent compounds. Whereas the reactions of ***o*** and ***m*** proceed instantly, the reaction with ***p*** takes 3 days until full conversion. The ΔGs calculated (Supplementary Fig. 62) in order to shed more light on the reactivity of parent carboranes are consistent with the experiment.

The ^1^H and ^13^C{^1^H} NMR spectra of compounds ***o*-2**, ***m*-2** and ***p*-2** have revealed patterns close to ***o*-1**, which suggests a symmetrical structure in the solution. This fact is further demonstrated by the ^11^B spectra, which exhibit analogous patterns with a smaller number of broad signals when compared to ***o*-1** (four signals for ***o*-2** and ***p*-2**; six signals for ***m*-2**). The solid-state structures of ***m*-2** and ***p*-2**, determined by scXRD methods, exhibit patterns similar to each other (Fig. 2), where the major difference is in the fact that the skeletal carbons in ***m*-2** are separated only by the upper vertex B6, but the carbons in positions 5 and 8 in ***p*-2** are arranged diagonally. In addition, carbene ligands are situated nearly perpendicularly in ***m*-2**, but they are almost parallel in ***p*-2**, which could also be related to the asymmetrical behaviour of ***m*-2**. In this molecule, the upper vertex B6 enters the virtual centre of the cage more closely than the B9 one, which is reflected in the magnitudes of the interatomic angles B4-B2-B6 [90.44(11)°] vs. B2-B4-B9 [97.50(9)°] and C11-B6-B9 [175.81(13)°] vs. C38-B9-B6 [159.83(11)°]. The lengths of the bonds between the upper vertex B atom and the carbene as well as the respective lower vertex are significantly different – B9-C38 1.577(3) and B6-C11 1.648(3) Å; B2-B6 1.863(3) and B4-B9 1.755(3) Å, whereas the other skeletal B–B and C–B bonds are similar to those of ***o*-1**. In the case of ***p*-2**, the alternating but symmetrical positions of the carbon atoms inside the cluster predetermine the averaging of the respective separations, such as 1.604(7) and 1.622(6) Å for the B-carbene ones. In fact, these structures evoke the structure suggested for the transition state of the isomerisation of ten-vertex clusters by Z-mechanism^43^.

Boranes ***o*-2**, ***m*-2** and ***p*-2** are crystalline compounds stable under an inert atmosphere, but their long-term heating (at 100 °C for 1 day in the case of ***p*-2**, for 1 month in the case of ***m*-2** and for 1 month to ~8% conversion in the case of ***o*-2**) in the THF solution surprisingly leads, in all cases, to their decomposition to one single product–the parent carborane ***p***. This transformation has been monitored by ^11^B NMR (see Supplementary Fig. 55). The ^1^H NMR spectra have revealed the decomposition of the carbene moiety into several undefined compounds. It is noteworthy that such isomerisation of parent carboranes ***o*** and ***m*** to the thermodynamically most stable ***p*** requires very harsh conditions (temperature above 350 °C)^44^.

**Fig. 3:**
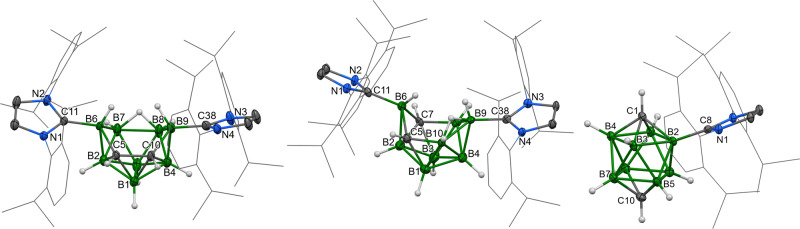
The molecular structures of *o*-2a (left), *m*-2a (centre) and *p*-2a (right). ORTEP-type plots, 40% probability level. Cl^−^ or HCl_2_^−^ anions and dichlormethane solvates are omitted for clarity. The 2,6-diisopropylphenyl groups are displayed as wireframes for clarity.

Compounds ***o*-1**, ***o*-2**, ***m*-2** and ***p*-2** were treated by hydrogen chloride in order to cleave the boron–carbene bond(s) and presumably obtain neutral open-cage *nido* or *arachno* compounds. Surprisingly, ***o*-1** did not even react with an overstoichiometric amount of hydrogen chloride—only a slow decomposition into several undefined compounds was observed after 1 week. On the contrary, ***o*-2** and ***m*-2** were unexpectedly converted into their corresponding hydrochlorides ***o*-2a** and ***m*-2a**, which is, to the best of our knowledge, the first example of a proton capture by a neutral borane cage leading to the formation of a cation without any changes in the functional groups. Such a reactivity is known for of amino, sulfido or phosphino based exoskeletal functional groups protonation or quaternization^30–32^. In this respect, there is only one example of the substitution of sulfide groups in bis-dialkylsulfido-dodecahydrodecaborane with hydrogen halogenides^45^. Both carboranes ***o*-2a** and ***m*-2a** are very stable–there was no decomposition observed after 1-month exposure to air and they do not react with the hydrogen chloride or even with the excess of triethylamine. The ^11^B NMR spectra of compounds ***o*-2a** and ***m*-2a** exhibit the same pattern as their precursors with only a few chemical-shift changes. This fact indicates only slight changes in the overall structure of the carborane cage, which was also confirmed by scXRD methods (Fig. 3).

The structures of both ***o*-2a** and ***m*-2a** cage parts closely resemble the neutral parent compound ***m*-2**, with the only exception being the elongation of the B7–B8 and B9–B10 bonds to ~1.84 Å, which is characteristic of boron atoms, bridged by the hydrogen atom originating from HCl. The difference between the position of that bridge in ***o*-2a** and ***m*-2a** is a result of the mutual positions of skeletal carbon atoms in each compound.

However, the most striking surprise has come in the reaction between hydrogen chloride with ***p*-2**, which undergoes cage closure, eliminating one molecule of dihydrogen (the gas evolution is even visible during the reaction), and the corresponding imidazolium chloride, forming ***p*-2a**. According to the ^1^H and ^11^B NMR spectra, the reaction is very pure (the combined amount of by-products is below 10%), but ***p*-2a** has been isolated only in the poor yield of 18% due to the multiple crystallisation required to purify it from the *N*,*N*-bis(2,6-diisopropylphenyl)imidazolium chloride, which exhibits very similar solubility in most of the common organic solvents. The ^11^B NMR spectrum contains one set of sharp signals in a very small range of chemical shifts (−11.1–(−13.6) ppm) with hardly any shift from the parent carborane ***p*** (−13.6 ppm). The significant stabilising effect of the carbene moiety is also demonstrated by the unexpected air stability of ***p*-2a**. The solid-state structure of this compound (Fig. 3 - right) is composed of the carbene-coordinated *closo*-ten-vertex cationic part compensated by the hydrogendichloride anion. All the C–B distances are comparable to the analogous separations in previously discussed compounds, whereas all the B–B separations of ~1.85 Å are even slightly longer than those reported for the other substituted *closo*-ten-vertex clusters^44^.

**Fig. 4:**
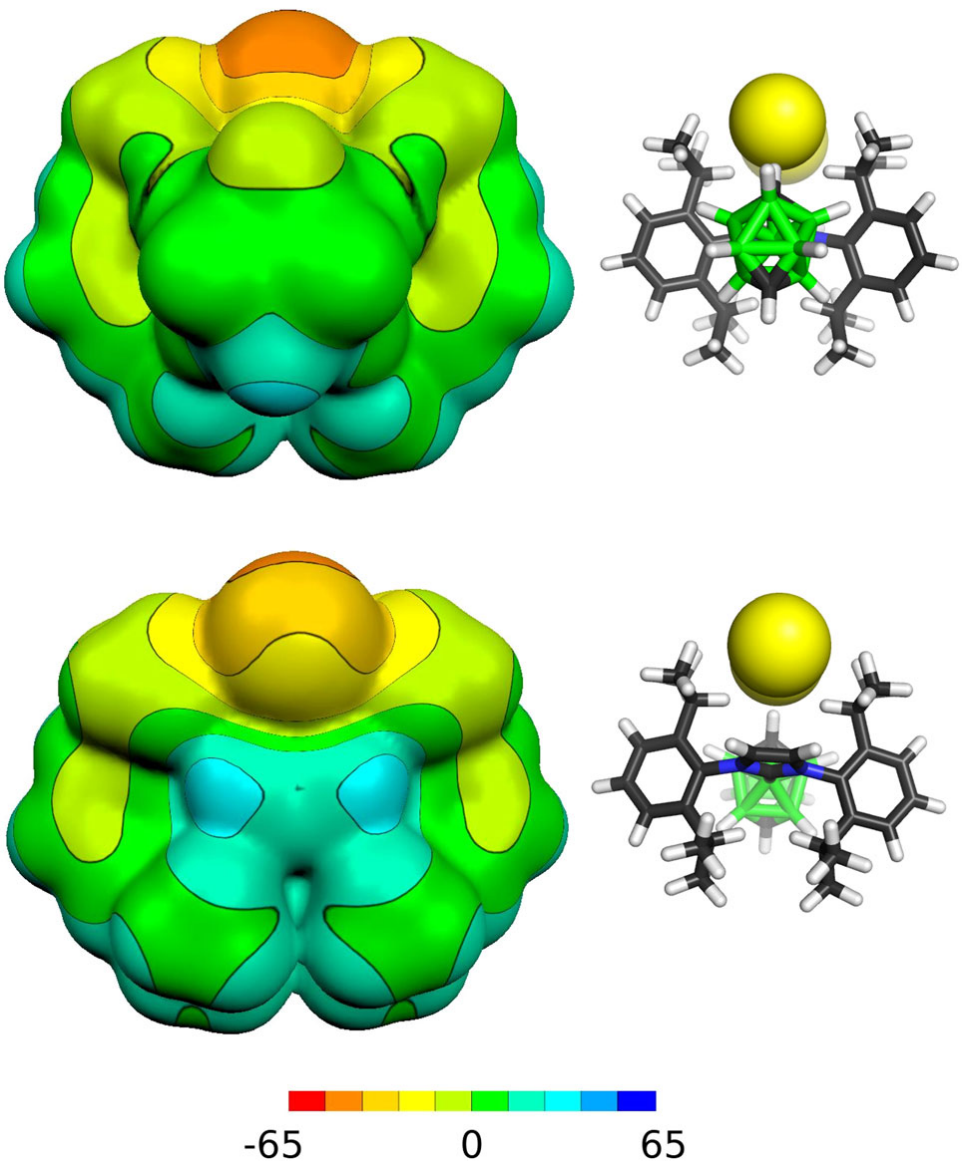
Computed (B3LYP/cc-pVTZ) ESP surface for *p-*2a. The colour range of the ESP in kcal/mol. The negative surface is located on HCl_2_^-^, the areas with the most positive ESP are the CH vertexes of the carborane cage and CH = CH fragment of the NHC.

The atomic charges of ***o*-1**, ***o*/*m*/*p*-2** and ***o*/*m*/*p*-2a** were calculated using AIM analysis along with electrostatic potential (ESP) surface (Fig. 4 and Supplementary Fig. 64 for the rest of ionic compounds) in order to shed more light on the charge distribution in prepared compounds. The central borane cluster C_2_B_8_H_10_ in ***o*-1** and ***o*/*m*/*p*-2** revealed slightly negative charge (−0.023 − (−0.044) e, for more details see Supplementary Table 11), while the carbene moieties showed slightly positive values (0.006–0.041 e) with the exception of ***m*-2** (+0.088 and −0.061 e), which is not surprising considering its unsymmetrical structure. The situation changes dramatically in the cationic series ***o*/*m*/*p*-2a**. The positive charge is predominantly localised in the boron cluster (0.692–0.713 e), however, the carbene groups still preserved the slightly positive charges (0.071–0.123 e).

The cationic character of the cages is also reflected in the fact that the compounds are sensitively detected in the positive ion mode. In contrast, electrospray-based mass spectrometry studies of the majority of borane and carborane compounds reveal the molecular peaks in the negative-ion mode^46^.

Having the cationic borane clusters in hand and bearing the fact that several boranes or carboranes exhibit promising biological activity in mind, we tested the solubility and stability of ***o*-2a** and ***p*-2** in water solution. Both compounds showed satisfactory solubility and appropriate NMR spectral patterns in D_2_O with no decomposition, and they had the same traces of ESI-MS spectra before and after a day in a water solution.

A logical way to investigate the reactivity of open heteroborane clusters is to increase the number of vertices by the incorporation of a new boron atom or a heteroatom. Therefore, we treated the carboranes ***o*-1** and ***o*-2**, ***m-*2** and ***p*-2** with phosphorus trichloride (Fig. 1). Both ***o*-1** and ***o*-2** were able to accommodate the phosphorus atom forming the heteroboranes ***o*-1a** and ***o*-2b**, whereas ***m*-2** and ***p*-2** gave only mixtures of various products, predominantly their hydrochlorides ***m*-2a** and ***p*-2a**. Several isomers and its derivatives of phosphadicarbaborane PC_2_B_8_H_11_ have already been published in the literature^47,48^, but there is no evidence of a cluster bearing the phosphorus atom inside the cage (positions 1–6). It is usually located in the upper five-membered ring. This might also be one of the reasons for the reactivity of ***o*-1a** with oxygen, because it reacts even with traces of air to the oxidised form ***o*-1b** with the phosphorus atom already situated in the position 2, which is in sharp contrast to the air-stable analogues published^47,48^.

Moreover, ***o*-1b** is a unique example of structurally characterised neutral heteroborane bearing an exoskeletal terminal double bond on a skeletal atom. Up to date, there are two structurally characterised anionic boranes with such a kind of bond, namely [*endo*-6(=S)-*exo*-6-R-*arachno*-6,7-PCB_8_H_11_] ^−^and [1,2-O(Ph)C_2_B_10_H_10_] ^−^^49^, which again proves the high stabilisation potential of the carbene group. The ^11^B NMR spectra of ***o*-1a** and ***o*-1b** have revealed the same pattern with small differences in chemical shifts. The ^31^P NMR spectra of compounds ***o*-1a**, ***o*-1b** and ***o*-2b** always revealed one broad signal for the skeletal phosphorus atom. In the case of ***o*-1a**, the chemical shift (−82.5 ppm) lies directly in the range of its published phosphacarborane analogues^47,48,50^ with the phosphorus atom in position 7 (−67.7–(−104.0) ppm). The oxidation of the phosphorus atom by oxygen is usually tied to higher shielding and an upfield shift of the ^31^P NMR signal. On the contrary, ***o*-1b** (32.0 ppm) is strongly downfield shifted, which corresponds to the changes in the bonding pattern. Finally, ***o*-2b** (97.1 ppm) has no structural analogue (central B_2_PCl_2_ pattern), and thus it could not be compared.

**Fig. 5:**
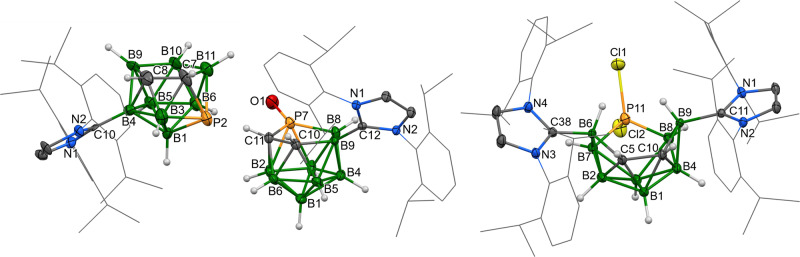
The molecular structures of *o*-1a (left), *o*-1b (centre) and *o*-2b (right). ORTEP-type plots, 40% probability level. Chloride anion and dichlormethane solvates are omitted for clarity in ***o*-2b**. The 2,6-diisopropylphenyl groups are displayed as wireframes for clarity.

The molecular structures (Fig. 5) of ***o*-1a** and ***o*-1b** are comparable in terms of C–C, C–B, P–B and P–C separations, but the placement of the phosphorus atom and carbene ligand in the lower pentagon together with four boron atoms in ***o*-1a** is quite different from the situation found in ***o*-1b**. Formally, the *nido*-type 11-vertex cluster ***o*-1b** has only three P–B bonds, and phosphorus is found in the upper open area. Moreover, the oxidation of the phosphorus atom has produced a very short P = O bond of 1.468(3) and 1.477(4) Å for two independent molecules.

Unexpectedly, the reaction of ***o*-2** with phosphorus trichloride did not proceed as a condensation, unlike in the case with ***o*-1**. At first glance, it seemed that the carborane ***o*-2** acted as a bidentate ligand coordinating the central phosphorus atom and forcing ionisation. The same side reaction has been observed as in cases of ***m*-2** and ***p*-2**, where the addition of phosphorus trichloride produces hydrogen chloride and subsequently compound ***o*-2a**. However, ***o*-2b** could still be isolated in moderate yield (38%) thanks to its poor solubility in THF. The ^11^B NMR spectrum has a pattern very similar to ***o*-2a**, with differences in chemical shifts of B1 (22 ppm upfield) and B3 (15 ppm downfield). When the structure of ***o*-2b** was inspected for the second time (Fig. 5), the PCl_2_^+^ fragment did not have a shape typical of P(III), but it could be described as a cationic structure of pentavalent phosphorus. This suggestion could be supported not only by the shape of the slightly distorted tetrahedron on the P atom, but also by the very short P–B distances [P11-B7 1.859(4) Å, P11-B8 1.856(4) Å]. In addition, the B7 and B8 atoms are bound to the rest of the boron atoms in the cage in a non-standard way, where one short [B2-B7 1.683(6) Å, B4-B8 1.687(6) Å] bond has been found for each of the B7 and B8 atoms, and the remaining separations are much longer than expected (~2 Å). To explain these parameters, one could suggest that the phosphorus atom shares the electron density of the former lone electron pair to the formation of strong connections to the B7 and B8 atoms, while these are somehow expelled from the shape of the previous ten-vertex cluster. Stabilisation of an electron-deficient group of the main-group element in ***o***-2b is a rare example of nucleophilic reactivity of a boron cluster and it would definitely not be possible without strong electron donation from the NHCs, which supports the C_2_B_6_H_8_ fragment interacting with the (BH)_2_PCl_2_^+^ part. The cationic character of the central heteroborane cage in ***o*-2b** was proved by the IBO and AIM analysis of bonding orbitals and the atomic charges, which revealed similar results when compared to ***o*/*m*/*p*-2a** (0.659 e, for more details, see Supplementary Table 12). By the same approach, the bonding pattern in the area of B6, P11 and B8 atoms was explored and estimated to be a shared interaction.

In this work, the involvement of NHCs in the chemistry of *closo*-ten-vertex carboranes leads surprisingly to the opening of *closo* types with no deprotonation or the loss of a boron vertex. Paradoxically, the introduction of adjacent-electron density into these structures by NHCs provides, after protonation by HCl, thermally robust cationic carboranes, which could potentially resolve the solubility and stability problems that often hamper the further applicability of this class of compounds. In addition, the reactivity of these NHC adducts with PCl_3_ results in uncommon cage patterns and redox activity.

## Supplementary information

Supplementary Information

## Data Availability

The crystallographic data for structural analysis have been deposited with the Cambridge Crystallographic Data Centre, CCDC nos. 2062928-2062936. Copies of this information may be obtained free of charge from The Director, CCDC, 12 Union Road, Cambridge CB2 1EY, UK (fax: +44-1223-336033; e-mail: deposit@ccdc.cam.ac.uk or www: http://www.ccdc.cam.ac.uk). The NMR data have been deposited at Figshare.com and are available at 10.6084/m9.figshare.15059430.v3.

## References

[1] Grimes RN (2016). Carboranes.

[2] Hosmane NS (2012). Boron Science: New Technologies and Applications.

[3] Hnyk D, McKee M (2015). Boron: The Fifth Element.

[4] Brown HC, Heim P (1964). Diborane as a mild reducing agent for the conversion of primary, secondary, and tertiary amides into the corresponding amines. J. Am. Chem. Soc..

[5] Douvris C, Ozerov OV (2008). Hydrodefluorination of perfluoroalkyl groups using silylium-carborane catalysts. Science.

[6] Yao SL (2020). Bis(silylene)-stabilized monovalent nitrogen complexes. Angew. Chem. Int. Ed..

[7] Yao SL, Kostenko A, Xiong Y, Růžička A, Driess M (2020). Redox noninnocent monoatomic silicon(0) complex (“silylone”): its one-electron-reduction induces an intramolecular one-electron-oxidation of silicon(0) to silicon(I). J. Am. Chem. Soc..

[8] Xiong Y, Yao S, Szilvási T, Růžička A, Driess M (2020). Homocoupling of CO and isocyanide mediated by a *C*,*C*′-bis(silylenyl)-substituted *ortho*-carborane. Chem. Commun..

[9] Xiong Y (2021). New types of Ge2 and Ge4 assemblies stabilized by a carbanionic dicarborandiyl-silylene ligand. J. Am. Chem. Soc..

[10] Yao, S. et al. Changing the reactivity of zero- and mono-valent germanium with a redox non-innocent bis(silylenyl)carborane ligand. *Angew. Chem. Int. Ed*. **60**, 14864–14868 (2021). 10.1002/anie.202103769 PMC8252802 33909944

[11] Légaré M-A (2020). One-pot, room-temperature conversion of dinitrogen to ammonium chloride at a main-group element. Nat. Chem..

[12] Légaré M-A (2019). The reductive coupling of dinitrogen. Science.

[13] Légaré, M.-A. et al. Nitrogen fixation and reduction at boron. *Science* **359**, 896–900 (2018). 10.1126/science.aaq1684 29472479

[14] Wang Y (2007). A stable neutral diborene containing a B-B double bond. J. Am. Chem. Soc..

[15] Légaré M-A, Pranckevicius C, Braunschweig H (2019). Metallomimetic chemistry of boron. Chem. Rev..

[16] Goettel, J. T. & Braunschweig, H. Recent advances in boron-centered ligands and their transition metal complexes. *Coord. Chem. Rev*. **380**, 184–200 (2019)

[17] Lu W, Do DCH, Kinjo R (2020). A flat carborane with multiple aromaticity beyond Wade-Mingos’ rules. Nat. Commun..

[18] Curran DP (2011). Synthesis and reactions of N‐heterocyclic carbene boranes. Angew. Chem. Int. Ed..

[19] Huynh HV (2017). The Organometallic Chemistry of N-Heterocyclic Carbenes.

[20] Fisher SP (2019). Nonclassical applications of *closo*-carborane anions: from main group chemistry and catalysis to energy storage. Chem. Rev..

[21] Hosmane, N. S. & Eagling, R. (eds) *Handbook of Boron Science with Applications in Organometallics, Catalysis, Materials and Medicine*: *Boron in Materials Chemistry* Vol. 3 (World Scientific Publishing Company, 2018).

[22] Brynda J (2013). Carborane-based carbonic anhydrase inhibitors. Angew. Chem. Int. Ed..

[23] Hosmane, N. S. & Eagling, R. *Handbook of Boron Science with Applications in Organometallics, Catalysis, Materials and Medicine: Boron in Medicine* Vol. 4 (World Scientific Publishing, 2018).

[24] Williams RE (1971). Carboranes and boranes; polyhedra and polyhedral fragments. Inorg. Chem..

[25] Wade, K. The structural significance of the number of skeletal bonding electron-pairs in carboranes, the higher boranes and borane anions, and various transition-metal carbonyl cluster compounds. *J. Chem. Soc. D* **15**, 792–793 (1971)

[26] Rudolph RW (1976). Boranes and heteroboranes: a paradigm for the electron requirements of clusters?. Acc. Chem. Res..

[27] Mingos, D. M. P. Polyhedral skeletal electron pair approach. A generalised principle for condensed polyhedra. *J. Chem. Soc. Chem. Commun*. **12**, 706–708 (1983)

[28] Mingos DMP (1984). Polyhedral skeletal electron pair approach. Acc. Chem. Res..

[29] Stauber JM (2020). A super-oxidized radical cationic icosahedral boron cluster. J. Am. Chem. Soc..

[30] Hamilton, E. J. M. et al. Unusual cationic tris(dimethylsulfide)-substituted closo-boranes: preparation and characterization of [1,7,9-(Me2S)3-B12H9] BF4 and [1,2,10-(Me2S)3-B10H7] BF4. *Inorg. Chem*. **51**, 2374–2380 (2012).. 10.1021/ic2023709 22309402

[31] Ioppolo JA, Clegg JK, Rendina LM (2007). Dicarba-closo-dodecaborane(12) derivatives of phosphonium salts: easy formation of nido-carborane phosphonium zwitterions. Dalton Trans..

[32] Kataki-Anastasakou. A. et. al. Carborane guests for cucurbit[7]uril facilitate strong binding and on-demand removal. *J. Am. Chem. Soc*. **142**, 20513–20518 (2020). 10.1021/jacs.0c09361 PMC8147132 33253553

[33] Willans CE, Kilner CA, Fox MA (2010). Deboronation and deprotonation of *ortho*‐carborane with N‐heterocyclic carbenes. Chem. Eur. J..

[34] Zheng F, Xie Z (2012). Reaction of *o*-carboranes with sterically demanding *N*-heterocyclic carbene: synthesis and structural characterization of 1:1 adducts. Dalton Trans..

[35] Vrána J (2019). Investigation of thiaborane *closo*–*nido* conversion pathways promoted by *N*-heterocyclic carbenes. Inorg. Chem..

[36] Wang H, Zhang J, Lin Z, Xie Z (2016). Synthesis and structural characterization of carbene-stabilized carborane-fused azaborolyl radical cation and dicarbollyl-fused azaborole. Organometallics.

[37] Wang H, Chan TL, Xie Z (2018). Cyclic amino(carboranyl) silylene: synthesis, structure and reactivity. Chem. Commun..

[38] Wang H, Zhang J, Xie Z (2017). Reversible photothermal isomerization of carborane-fused azaborole to borirane: synthesis and reactivity of carbene-stabilized carborane-fused borirane. Angew. Chem. Int. Ed..

[39] Zheng F, Xie Z (2015). Reaction of N-heterocyclic carbenes with 13-vertex *closo*-carboranes: synthesis and structural characterization of zwitterionic salts of 13-vertex *nido*-carboranes. Org. Chem. Front..

[40] Zhang J, Xie Z (2014). Synthesis, structure, and reactivity of 13- and 14-vertex carboranes. Acc. Chem. Res..

[41] Adams RD, Kiprotich J, Peryshkov DV, Wong YO (2016). Cage opening of a carborane ligand by metal cluster complexes. Chem. Eur. J..

[42] Tok, O. L. et al. Click dehydrogenation of carbon-substituted *nido*-5,6-C_2_B_8_H_12_ carboranes: a general route to *closo*-1,2-C_2_B_8_H_10_ derivatives. *Inorg. Chem*. **55**, 8839–8843 (2016). 10.1021/acs.inorgchem.6b01386 27551885

[43] Bakardjiev, M. Transformation of various multicenter bondings within bicapped-square antiprismatic motifs: Z-rearrangement. *Dalton Trans*. 10.1039/D0DT04225K (2021). 10.1039/d0dt04225k 33656022

[44] Bakardjiev M, Stibr B, Holub J, Padělková Z, Růžička A (2015). Simple synthesis, halogenation, and rearrangement of *closo*-1,6-C_2_B_8_H_10_. Organometallics.

[45] Plesek J, Stibr B, Hermanek S (1966). Chemistry of boranes. VI. The reaction of bis-dialkylsulphido-dodecahydrodecaboranes with hydrohalogens. General preparation of 6- (or 5-)halogentridecahydrodecaboranes. Collect Czech. Chem. Commun..

[46] Henderson W. & McIndoe J. S. *Mass Spectrometry of Inorganic, Coordination and Organometallic Compounds: Tools - Techniques – Tips* (Wiley, 2005).

[47] Štíbr, B. et al. Phosphacarborane chemistry: the synthesis of the parent phosphadicarbaboranes nido-7,8,9-PC_2_B_8_H_11_ and [*nido-*7,8,9-PC_2_B_8_H_10_]^−^, and their 10-Cl derivatives–analogs of the cyclopentadienide anion. *Eur. J. Inorg. Chem*. **9**, 2320–2326 (2002)

[48] Kudinov, A. R. et al. Synthesis, structure, electrochemistry, and Mössbauer effect studies of the ferraphosphadicarbollides [(C_5_R_5_)Fe(PC_2_B_8_H_10_)] (R = H, Me). *Eur. J. Inorg. Chem*. **13**, 4190–4196 (2007).

[49] Brown, D. A. et al. A Pentuply-bridging carbonyl group: crystal and molecular structure of a salt of the l-Oxo-2-phenyl-1,2-dicarbadodecaborate(12) anion, [H] ^+^[O(Ph)C_2_B_10_H_10_]^-^(L = 1,8-*N*,*N*,*N’*,*N’*-tetramethylnaphthalenediamine). *J. Chem. Soc. Chem. Commun*. **12**, 889–891 (1987).

[50] Holub J, Ormsby DL, Kennedy JD, Greatrex R, Štíbr B (2000). Phosphacarborane chemistry. New cluster isomers in the eleven-vertex nido-phosphadicarbaborane series: synthesis of the nido phosphadicarbaboranes 7,8,11-PC_2_B_8_H_11_, [7,8,11-PC_2_B_8_H_10_]^−^ and 7-Ph-7,8,10-PC_2_B_8_H_10_. Inorg. Chem. Commun..

