# Supplementary material for: Access to cationic polyhedral carboranes via dynamic cage surgery with N-heterocyclic carbenes
Source: Nat Commun. 2021 Aug 17;12:4971. doi: 10.1038/s41467-021-25277-0 (PMC8371172; doi:10.1038/s41467-021-25277-0)
Supplement: Supplementary file 1 — Supplementary Information [file 41467_2021_25277_MOESM1_ESM.pdf]

Access to Cationic Polyhedral Carboranes via dynamic cage surgery with *N*-heterocyclic carbenes

Jan Vrána,[1] Josef Holub,[2] Maksim A. Samsonov,[1] Zdeňka Růžičková,[1] Josef Cvačka,[3] Michael L. McKee,[4] Jindřich Fanfrlík,[3] Drahomír Hnyk[2] and Aleš Růžička[1]*

* e-mail: ales.ruzicka@upce.cz

[1] Department of General and Inorganic Chemistry, Faculty of Chemical Technology, University of Pardubice, Studentská 573, 532 10 Pardubice, Czech Republic, e-mail: ales.ruzicka@upce.cz

[2] Institute of Inorganic Chemistry, Czech Academy of Sciences, 250 68 Řež, Czech Republic

[3] Institute of Organic Chemistry and Biochemistry of the Czech Academy of Sciences, Flemingovo náměstí 542/2, 166 10 Praha 6, Czech Republic

[4] Department of Chemistry and Biochemistry, Auburn University, Auburn, AL, 36849, USA.

Table of Contents

Supplementary Methods......S3

Synthesis of 10-NHC^{Dip}-5,6-$C_2B_8H_{10}$ (***o*-1**)...... S5

Synthesis of 6,9-NHC$^{Dip}{}_2$-5,10-$C_2B_8H_{10}$(***o*-2**)......S8

Synthesis of 6,9-NHC$^{Dip}{}_2$-5,7-$C_2B_8H_{10}$ (***m*-2**)......S11

Synthesis of 6,9-NHC$^{Dip}{}_2$-5,8-$C_2B_8H_{10}$ (***p*-2**)......S15

Synthesis of [6,9-NHC$^{Dip}{}_2$-5,10-$C_2B_8H_{11}$]$^+$Cl$^-$ (***o*-2a**)...... S19

Synthesis of [6,9-NHC$^{Dip}{}_2$-5,7-$C_2B_8H_{11}$]$^+$[HCl_2]$^-$ (***m*-2a**)......S23

Synthesis of [2-NHC^{Dip}-1,10-$C_2B_8H_9$]$^+$[HCl_2]$^-$ (***p*-2a**)......S27

Synthesis of 4-NHC^{Dip}-2,7,8-$PC_2B_8H_9$ (***o*-1a**)...... S31

Synthesis of 10-NHC^{Dip}-7-(=O)-7,8,9-$PC_2B_8H_9$ (***o*-1b**)...... S35

Synthesis of [11,11-Cl_2-5,10-NHC$^{Dip}{}_2$-11,5,10-$PC_2B_8H_{10}$]$^+$Cl$^-$ (***o*-2b**)......S39

Supplementary Discussion......S43

Position of the hydrogen bridge in ***o*-1**, ***o*-2** and ***o*-2a**...... S43

Thermal decomposition of ***o*-2**, ***m*-2** and ***p*-2**......S44

Synthesis of ***o*-1a** and ***o*-1b**......S46

Supramolecular architecture of ***o*-2a**, ***m*-2a** and ***p*-2a**...... S47

Supplementary Computational Data......S48

NMR shifts......S48

Reactions of ***o***, ***m*** and ***p*** with :IPr S49

QTAIM...... S49

ESP...... S52

IBO...... S52

Supplementary References...... S54

Supplementary Methods

NMR spectroscopy

^{1}H, ^{11}B and ^{13}C NMR spectra were recorded on Bruker Avance 500 MHz spectrometer or Bruker Ultrashield 400 MHz, using 5 mm tuneable broad-band probe. Appropriate chemical shifts in ^{1}H and ^{13}C NMR spectra were related to the residual signals of the solvents ($CDCl_3$: $\delta(^{1}H)$ = 7.24 ppm and $\delta(^{13}C)$ = 77.23 ppm; acetone-d6: $\delta(^{1}H)$ = 2.05 ppm and $\delta(^{13}C)$ = 29.92 ppm; CD_2Cl_2: $\delta(^{1}H)$ = 5.33 ppm and $\delta(^{13}C)$ = 54.24 ppm; THF-d_8: $\delta(^{1}H)$ = 1.73 and 3.58 ppm and $\delta(^{13}C)$ = 25.37 and 67.57 ppm). ^{11}B chemical shifts were related to external standard BF_3•OEt_2 ($\delta(^{11}B)$ = 0.0 ppm). Remarkably, ^{11}B-^{11}B NMR has revealed no crosspeaks at all, even in a broad range of temperatures (−50–+50 °C), most probably due to some dynamic process in solution. The assignment of the spectra was based on correlation with other compounds prepared in this paper or in the literature, and it was supported by a computational study using the GIAO method (see the Quantum-chemical section), which was in very good agreement with the experimental data. ^{31}P chemical shifts were related to external standard 85% H_3PO_4 in water ($\delta(^{31}P)$ = 0.0 ppm).

Mass spectrometry

Mass spectra were recorded using the LTQ Orbitrap XL hybrid mass spectrometer (Thermo Fisher Scientific, Waltham, MA, USA) at a resolution of 100,000. The electrospray ion source was operated in the positive ion mode with the spray voltage, capillary voltage, tube lens voltage, and capillary temperature set at 4.9 kV, 35 V, 145 V, and 275 °C, respectively. The sample was dissolved in acetonitrile and injected using a 5-µL loop into methanol/water (4:1; 100 µL/min) mobile phase. The mass spectra were internally calibrated using protonated phthalic anhydride lock mass. Mass spectrometry measurements of the D_2O solutions were performed on a Thermo-Finnigan LCQ-Fleet Ion Trap instrument using electrospray ionization (ESI) for ionic species with detection of positive ions. For ESI, samples dissolved in acetonitrile (concentrations approximately 100 ng/mL) were introduced to the ion source by infusion. Molecular ions $[M]^+$ were detected for both univalent cations as base peaks in the spectra.

sc-XRD

Full-sets of diffraction data for ***o*-1**, ***o*-1a**, ***o*-1b**, ***o*-2a**, ***o*-2b**, ***m*-2**, ***m*-2a**, ***p*-2** and ***p*-2a** were collected at 150(2)K with a Bruker D8-Venture diffractometer equipped with Cu (Cu/Kα radiation; λ = 1.54178 Å for ***o*-1** and ***p*-2**) or Mo (Mo/Kα radiation; λ = 0.71073 Å rest of the compounds) microfocus X-ray (IµS) sources, Photon CMOS detector and Oxford Cryosystems cooling device was used for data collection.

The frames were integrated with the Bruker SAINT software package using a narrow frame algorithm. Data were corrected for absorption effects using the Multi-Scan method (SADABS). Obtained data were treated by XT-version 2014/5 and SHELXL-2017/1 software implemented in APEX3 v2016.5-0 (Bruker AXS) system.[5]

Hydrogen atoms were mostly localized on a difference Fourier map, however to ensure uniformity of treatment of crystal, all hydrogen were recalculated into idealized positions (riding model) and assigned temperature factors Hiso(H) = 1.2 Ueq (pivot atom) or of 1.5 Ueq (methyl). H atoms in methyl, methylene, methine, and hydrogen atoms in aromatic rings were placed with C-H distances of 0.96, 0.97, 0.98, and 0.93 Å and 1.1 Å for terminal B-H (but most of them were refined freely) bonds.

Minor disorders of parts of Dipp groups or solvent molecules in ***o*-1b**, ***o*-2a**, ***o*-2b** and ***p*-2** were treated by standard methods. In ***m*-2a** and ***o*-1a**, the principal disorders of the carborane cages was solved by splitting them to two positions. In ***o*-1a**, which was refined as a 2-component twin, the R^2 parameter value produces an A-alert during checkcif procedure, but the uniformity of the structure is clear enough.

$R_{int} = \sum|F_o^2 - F_{o,mean}^2|/\sum F_o^2$, GOF = $[\sum(w(F_o^2 - F_c^2)^2)/(N_{diffrs} - N_{params})]^{½}$ for all data, $R(F) = \sum||F_o| - |F_c||/\sum|F_o|$ for observed data, $wR(F^2) = [\sum(w(F_o^2 - F_c^2)^2)/(\sum w(F_o^2)^2)]^{½}$ for all data.

Crystallographic data for structural analysis have been deposited with the Cambridge Crystallographic Data Centre, CCDC nos. 2062928-2062936. Copies of this information may be obtained free of charge from The Director, CCDC, 12 Union Road, Cambridge CB2 1EY, UK (fax: +44-1223-336033; e-mail: deposit@ccdc.cam.ac.uk or www: http://www.ccdc.cam.ac.uk).

Synthesis

All manipulations were carried out under an argon atmosphere using standard Schlenk tube technique. Solvents were dried using a Pure Solv–Innovative Technology equipment under argon gas atmosphere. Starting compounds *closo*-*o*-$C_2B_8H_{10}$,[1] *closo*-*m*-$C_2B_8H_{10}$,[2] *closo*-*p*-$C_2B_8H_{10}$,[3] and NHC^{Dip} [4] were prepared according to the published procedures, however, these compounds are all commercially available. Elemental analyses were performed on an LECO-CHNS-932 analyzer.

Synthesis of 10-NHC^{Dip}-5,6-$C_2B_8H_{10}$ (*o*-1)

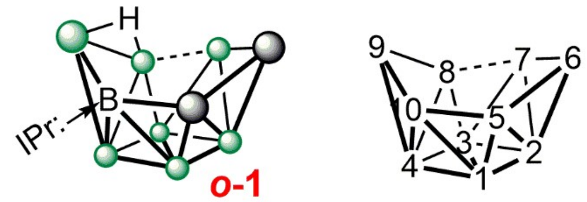

Solution of NHC^{Dip} (254 mg, 0.65 mmol) in diethyl ether (5 mL) was added dropwise to a stirred solution of *closo-o*-$C_2B_8H_{10}$ (79 mg, 0.65 mmol) in diethyl ether (5 mL) at room temperature. The stirring bar was removed and the solution was left for crystallization overnight. Colorless single crystals of ***o*-1** were decanted and dried in vacuo. Yield 226 mg, 68 %. **Dp** 127 °C. **Anal. Calc**. for $C_{29}H_{46}B_8N_2$ (509.18): C 68.4, H 9.1, N 5.5; found C 68.5, H 9.1, N 5.4. **^{1}H NMR** (25 °C, thf-d_8, 500 MHz): δ = 1.11 (d, $^3J(^1H-^1H)$ = 6.8 Hz, 6H, CH(CH_3)$_2$), 1.12 (d, $^3J(^1H-^1H)$ = 6.8 Hz, 6H, CH(CH_3)$_2$), 1.27 (d, $^3J(^1H-^1H)$ = 6.7 Hz, 6H, CH(CH_3)$_2$), 1.36 (d, $^3J(^1H-^1H)$ = 6.7 Hz, 6H, CH(CH_3)$_2$), 2.62 (s broad, 1H, BC*H*), 2.79 (sept, $^3J(^1H-^1H)$ = 6.8 Hz, 2H, C*H*(CH$_3$)$_2$), 2.91 (sept, $^3J(^1H-^1H)$ = 6.7 Hz, 2H, CH(CH_3)$_2$), 4.02 (s broad, 1H, BC*H*), 7.34 (d, $^3J(^1H-^1H)$ = 7.8 Hz, 2H, *m*-Ar*H*), 7.42 (d, $^3J(^1H-^1H)$ = 7.8 Hz, 2H, *m*-Ar*H*), 7.55 (t, $^3J(^1H-^1H)$ = 7.8 Hz, 2H, *p*-Ar*H*), 7.86 (s, 2H, C*H*=C*H*) ppm. **^{11}B NMR** (25 °C, thf-d_8, 160.42 MHz): δ = -32.1 (d, $^1J(^1H-^{11}B)$ = 168 Hz, 1B, B4), -28.0 (d, $^1J(^1H-^{11}B)$ = 142 Hz, 1B, B2), -15.8 (s, 1B, B10), -14.0 (d, $^1J(^1H-^{11}B)$ = 142 Hz, 1B, B3), -3.4 (d, $^1J(^1H-^{11}B)$ = 142 Hz, 1B, B8), -1.2 (s broad, 1B, B1), 10.3 (s broad, 1B, B7), 18.6 (s broad, 1B, B9) ppm. **^{13}C{^{1}H} NMR** (25 °C, thf-d_8, 125.76 Hz): δ = 22.2, 22.9, 25.8, 26.0 (s, CH(CH$_3$)$_2$), 29.6, 29.7 (s, *C*H(CH$_3$)$_2$), 56.4 (s broad, B*C*H), 105.4 (s broad, B*C*H), 124.9, 125.0 (s, *m*-Ar*C*), 125.9 (s, *C*H=*C*H), 131.8 (s, *p*-Ar*C*), 134.7 (s, *ipso*-Ar*C*), 146.71, 146.74 (s, *o*-Ar*C*), 164.7 (s very broad, N*C*N) ppm.

Spectroscopic characterization of 7-NHC^{Dip}-5,6-$C_2B_8H_{10}$ (*o*-1)

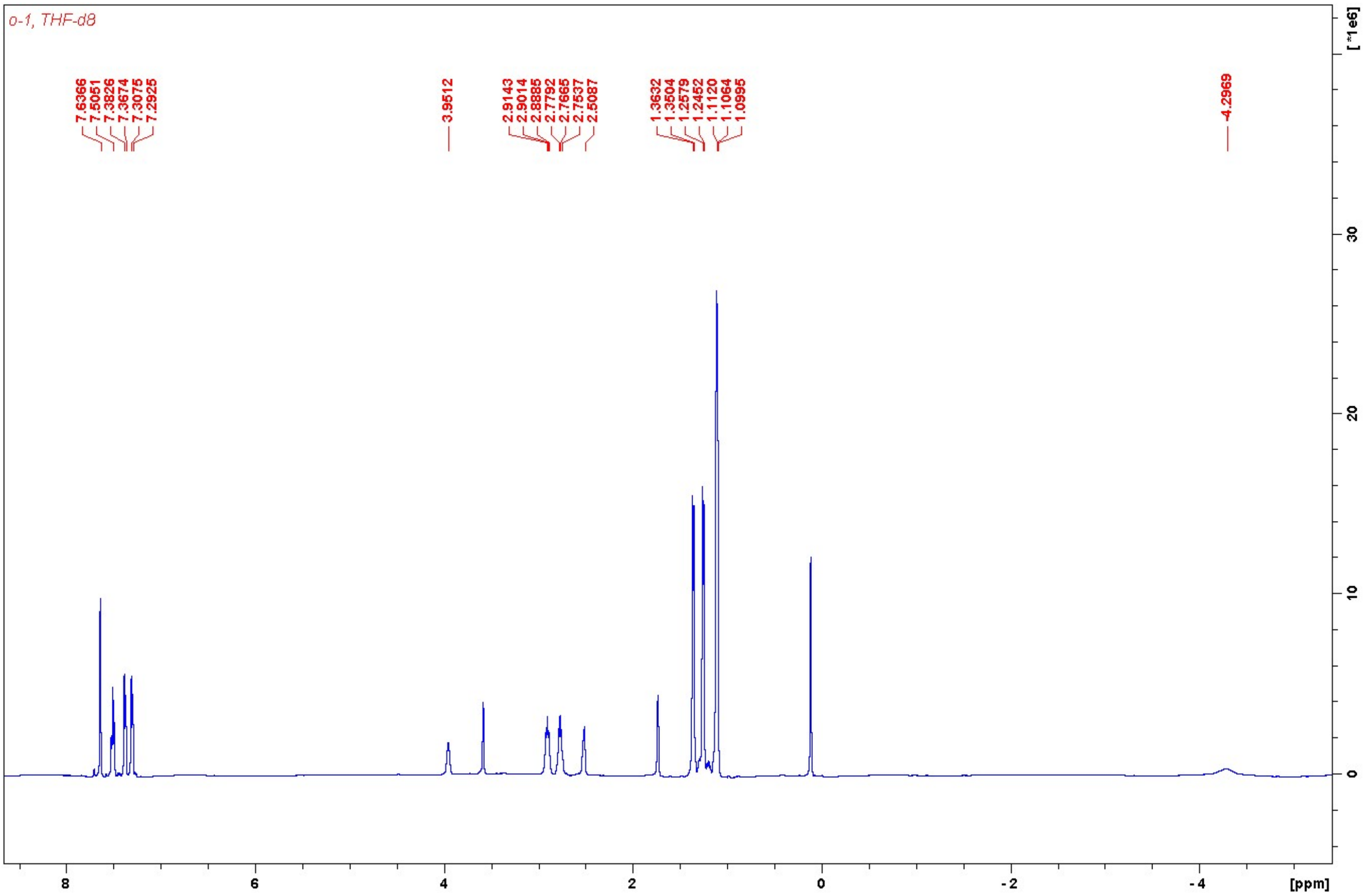

Supplementary Figure 1. ^{1}H NMR spectrum of *o*-1.

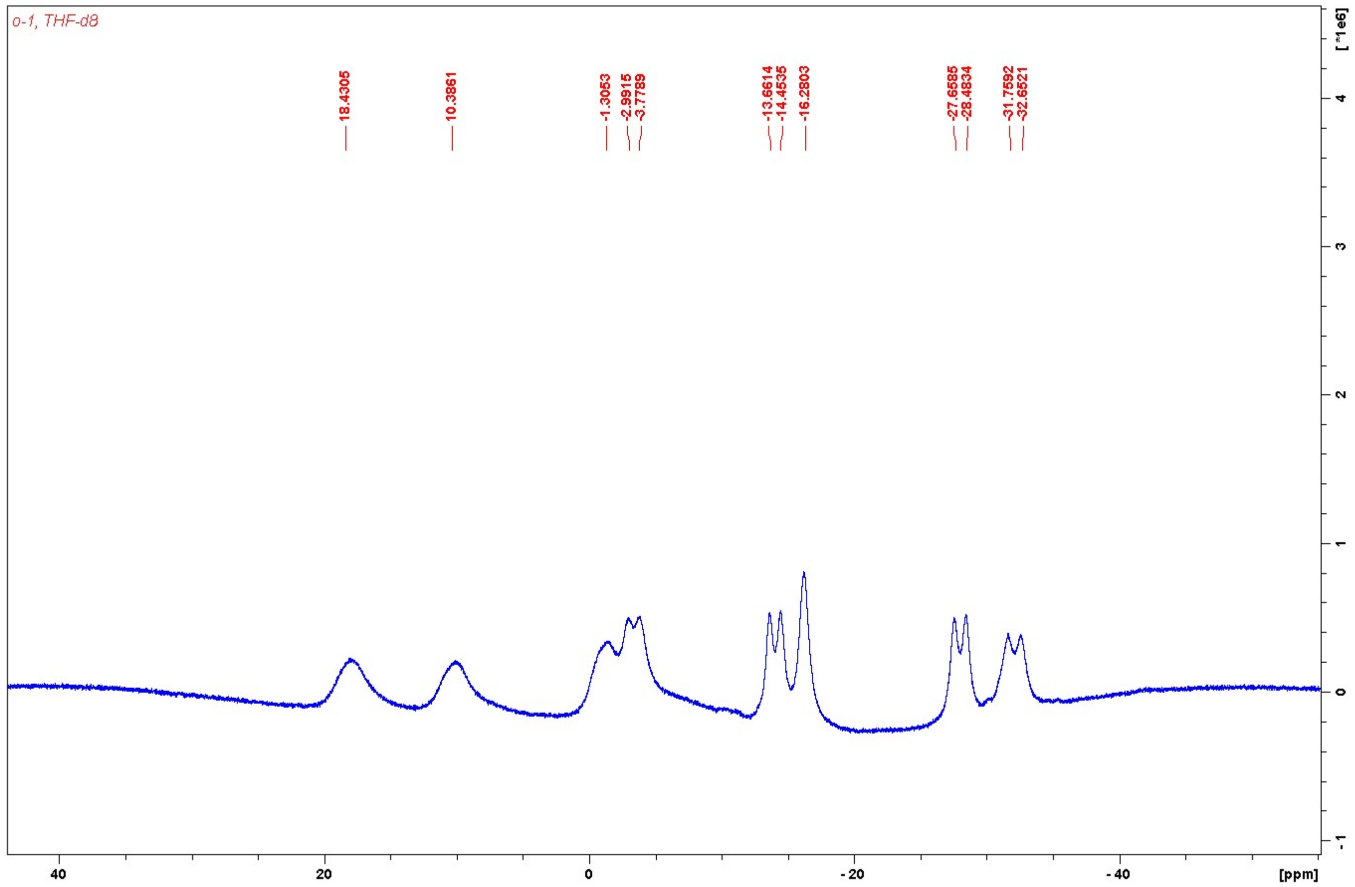

Supplementary Figure 2. ^{11}**B NMR spectrum of** ***o*****-1.**

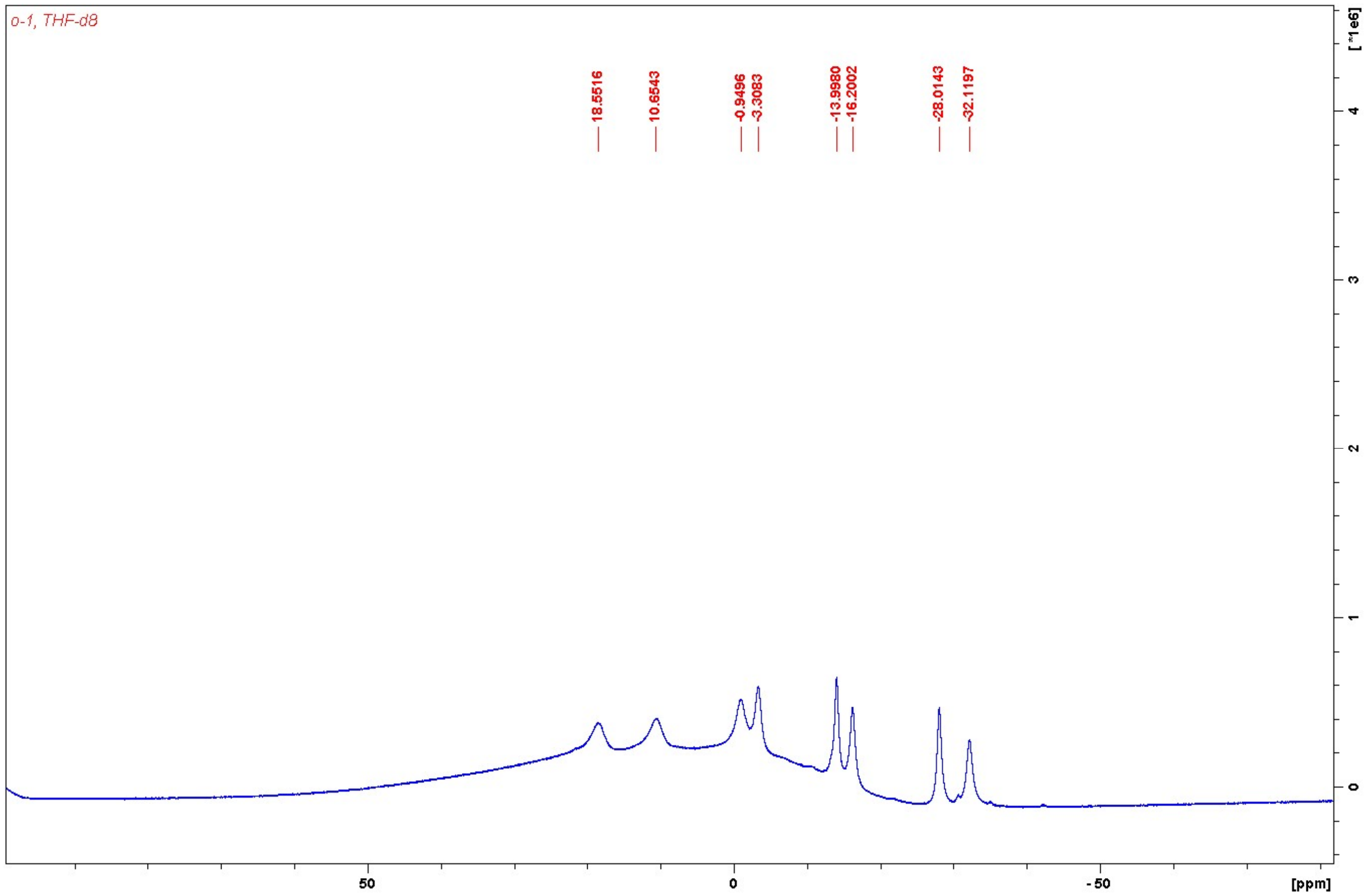

Supplementary Figure 3. ^{11}**B{**^{1}**H} NMR spectrum of** ***o*****-1.**

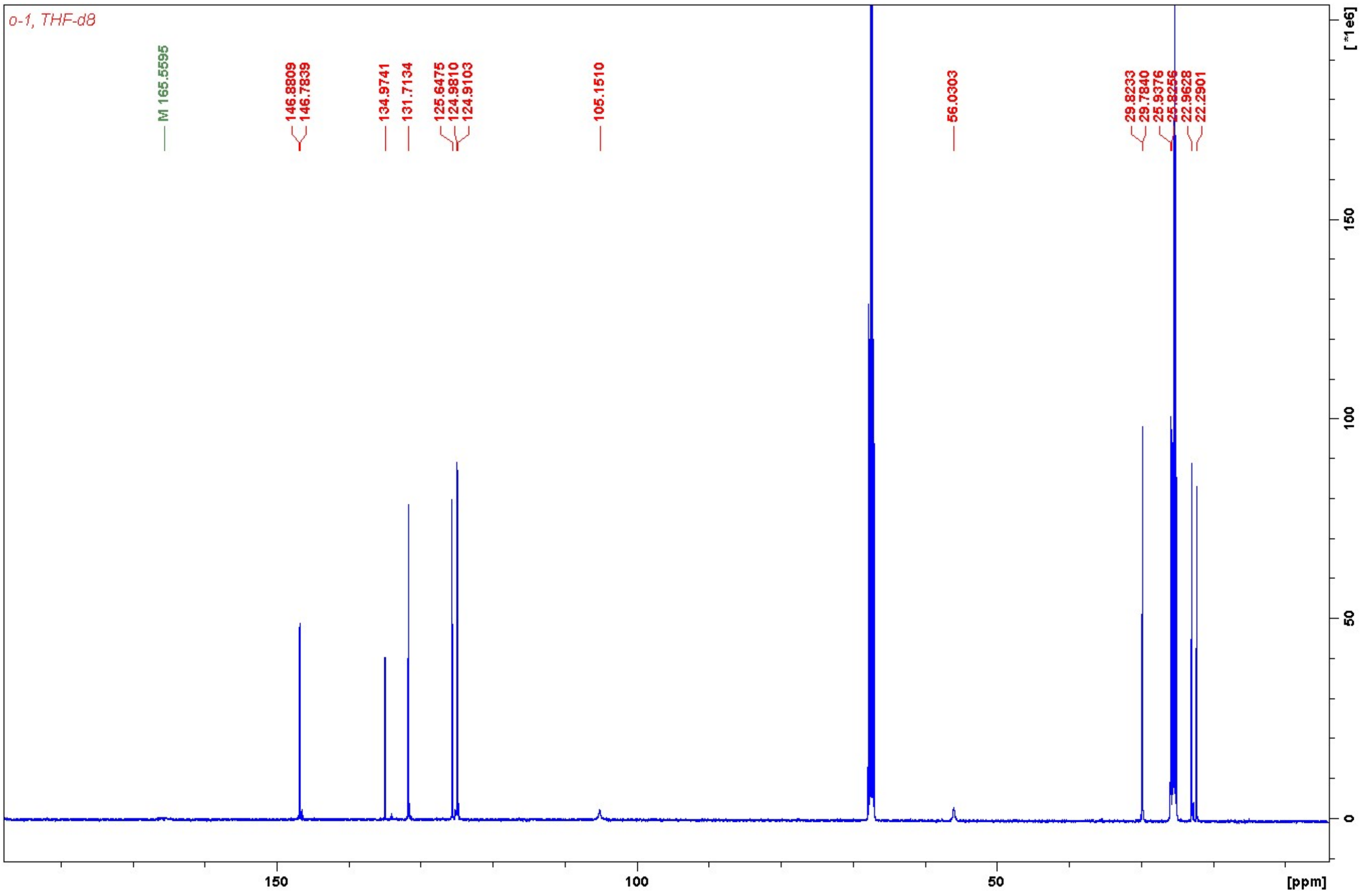

Supplementary Figure 4. $^{13}C\{^1H\}$ **NMR spectrum of *o*-1.**

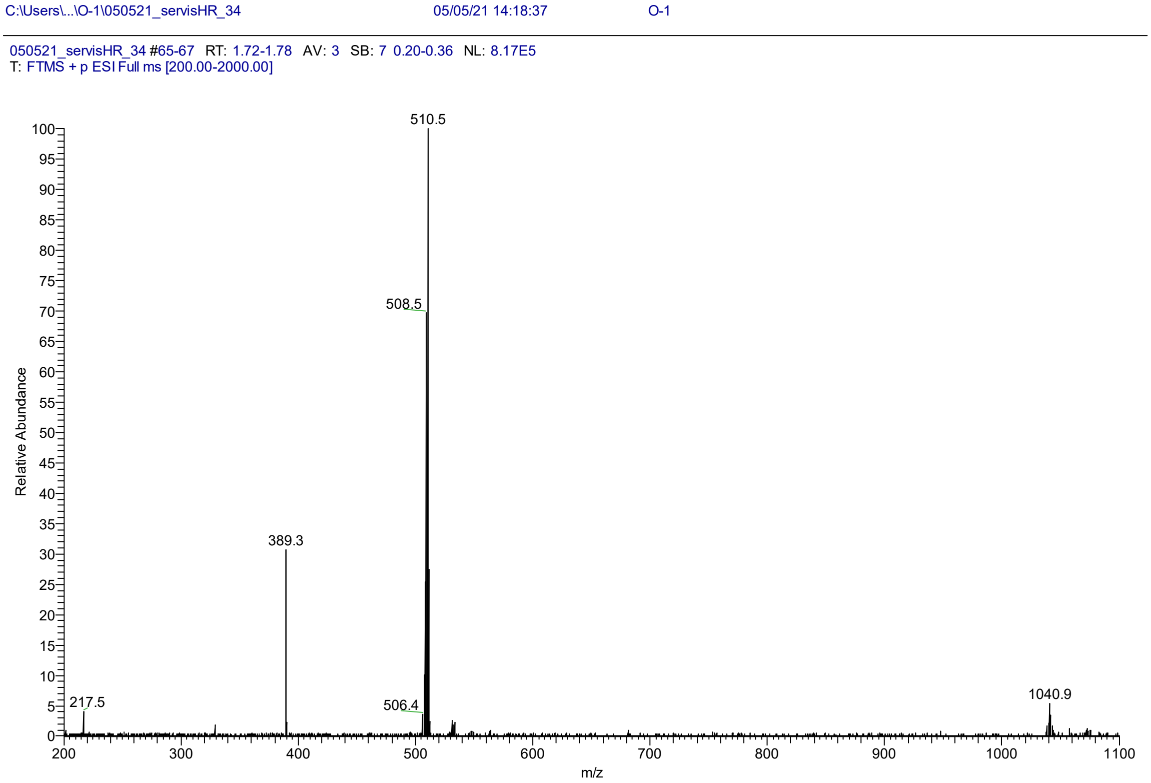

Supplementary Figure 5. MS spectrum (ESI+) of *o*-1: [:IPr + H]$^+$, *m/z* 389.3; [M + H]$^+$, *m/z* 511.4; [2M + Na]$^+$, *m/z* 1043.9. HRMS (ESI+): *m/z* calculated for $C_{29}H_{47}N_2B_8^+$ 511.44782, found 511.44805 (0.45 ppm).

Supplementary Table 1. Crystal data and structure refinement for *o*-1.

Chemical formula	$C_{29}H_{46}B_8N_2$
M_r	509.16
Crystal system, space group	Tetragonal, $I4_1cd$
Temperature (K)	150
a, *c* (Å)	25.2090 (4), 20.3708 (4)
V (Å^3)	12945.5 (5)
Z	16
Radiation type	Cu *K*α
μ (mm^{-1})	0.41
Crystal size (mm)	0.36 × 0.26 × 0.13
Data collection	
Diffractometer	Bruker D8 - Venture
Absorption correction	Multi-scan<br>*SADABS2016*/2 - Bruker AXS area detector scaling and absorption correction
T_{min}, T_{max}	0.658, 0.754
No. of measured, independent and<br>observed [$I > 2\sigma(I)$] reflections	83136, 6945, 6386
R_{int}	0.063
$(\sin \theta/\lambda)_{max}$ (Å^{-1})	0.637
Refinement	
$R[F^2 > 2\sigma(F^2)]$, $wR(F^2)$, S	0.043, 0.106, 1.06
No. of reflections	6945
No. of parameters	400
No. of restraints	370
H-atom treatment	H atoms treated by a mixture of independent and constrained refinement
$\Delta\rho_{max}$, $\Delta\rho_{min}$ (e Å^{-3})	0.20, −0.21
Absolute structure	Flack x determined using 2778 quotients [(I+)-(I-)]/[(I+)+(I-)] (Parsons, Flack and Wagner, Acta Cryst. B69 (2013) 249-259). Refined as a 2-component twin.
Absolute structure parameter	−0.12 (11) – this value is meaningless as the crystal is weak anomalous scatterer

Synthesis of 6,9-NHC^{Dip}$_2$-5,10-$C_2B_8H_{10}$ (*o*-2)

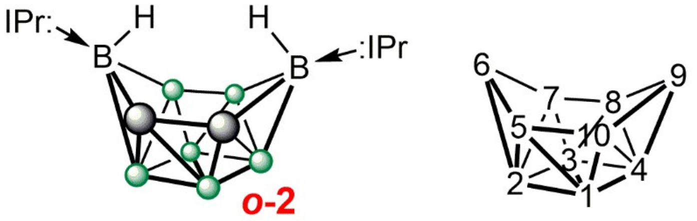

Solution of *closo*-*o*-$C_2B_8H_{10}$ (89 mg, 0.74 mmol) in diethyl ether (5 mL) was added dropwise to a stirred solution of NHC^{Dip} (688 mg, 1.77 mmol) in diethyl ether (15 mL) at room temperature. The slightly yellow reaction mixture was stirred for one hour and then the volatiles were removed in vacuo. The crude product was washed with hexane (15 mL) to remove the excess NHC^{Dip}. The slightly yellow powder of ***o*-2** was dried in vacuo. Yield 629 mg, 95 %. **Dp** 145 °C. **Anal. Calc.** for $C_{56}H_{82}B_8N_4$ (897.77): C 74.9, H 9.2, N 6.2; found C 75.0, H 9.3, N 6.2**.** **^{1}H NMR** (25 °C, thf-d$_8$, 500 MHz): δ = 0.09 (s broad, 2H, BC*H*), 1.04 (d, $^3J(^1H-^1H)$ = 6.7 Hz, 12H, CH(C*H*$_3$)$_2$), 1.06 (d, $^3J(^1H-^1H)$ = 6.7 Hz, 12H, CH(C*H*$_3$)$_2$), 1.23 (d, $^3J(^1H-^1H)$ = 6.8 Hz, 24H, CH(C*H*$_3$)$_2$), 2.53 (sept, $^3J(^1H-^1H)$ = 6.7 Hz, 4H, C*H*(CH$_3$)$_2$), 2.91 (sept, $^3J(^1H-^1H)$ = 6.7 Hz, 4H, CH(C*H*$_3$)$_2$), 7.08 (s, 4H, C*H*=C*H*), 7.14 (d, $^3J(^1H-^1H)$ = 7.8 Hz, 4H, *m*-Ar*H*), 7.15 (d, $^3J(^1H-^1H)$ = 7.8 Hz, 4H, *m*-Ar*H*), 7.34 (t, $^3J(^1H-^1H)$ = 7.8 Hz, 4H, *p*-Ar*H*) ppm. **^{11}B NMR** (25 °C, thf-d$_8$, 160.42 MHz): δ = -52.3 (d, $^1J(^1H-^{11}B)$ = 173 Hz, 1B, B3), -37.1 (d, $^1J(^1H-^{11}B)$ = 149 Hz, 3B, B1,6,9), -8.2 (s broad, 2B, B7,8), -4.8 (s broad, 3B, B2,4) ppm. **^{13}C{^{1}H} NMR** (25 °C, thf-d$_8$, 125.76 Hz): δ = 20.3 (s broad, B*C*H), 23.4, 24.1, 25.9, 26.0 (s, CH(*C*H$_3$)$_2$), 29.7, 29.8 (s, *C*H(CH$_3$)$_2$), 124.0 (s, *C*H=*C*H), 124.3, 124.6 (s, *m*-Ar*C*), 130.5 (s, *p*-Ar*C*), 136.1 (s, *ipso*-Ar*C*), 146.5, 147.0 (s, *o*-Ar*C*), 174.0 (s very broad, N*C*N) ppm.

Spectroscopic characterization of 6,9-NHC$^{Dip}{}_2$-5,10-$C_2B_8H_{10}$ (*o*-2)

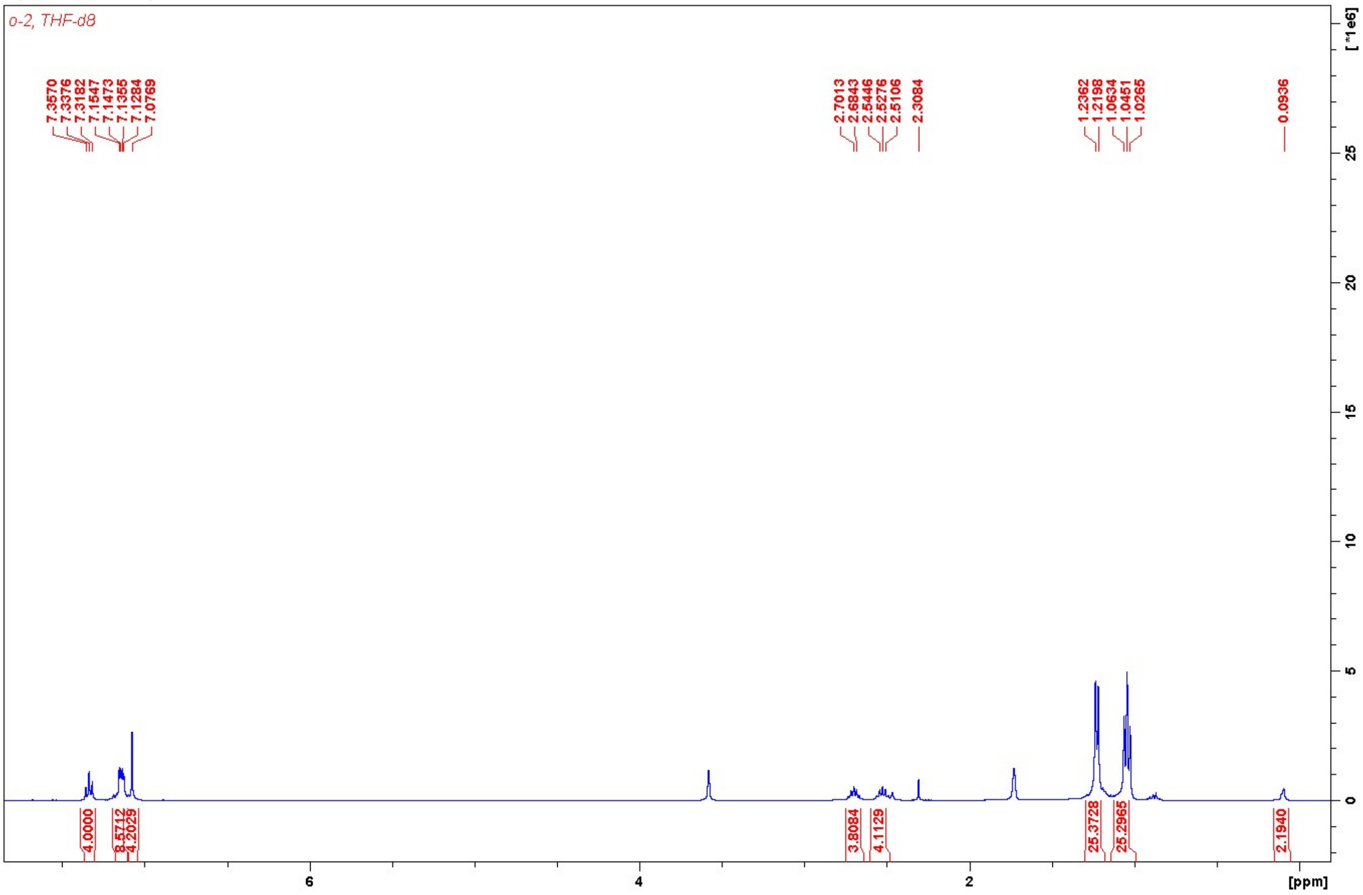

Supplementary Figure 6. ^{1}H NMR spectrum of *o*-2.

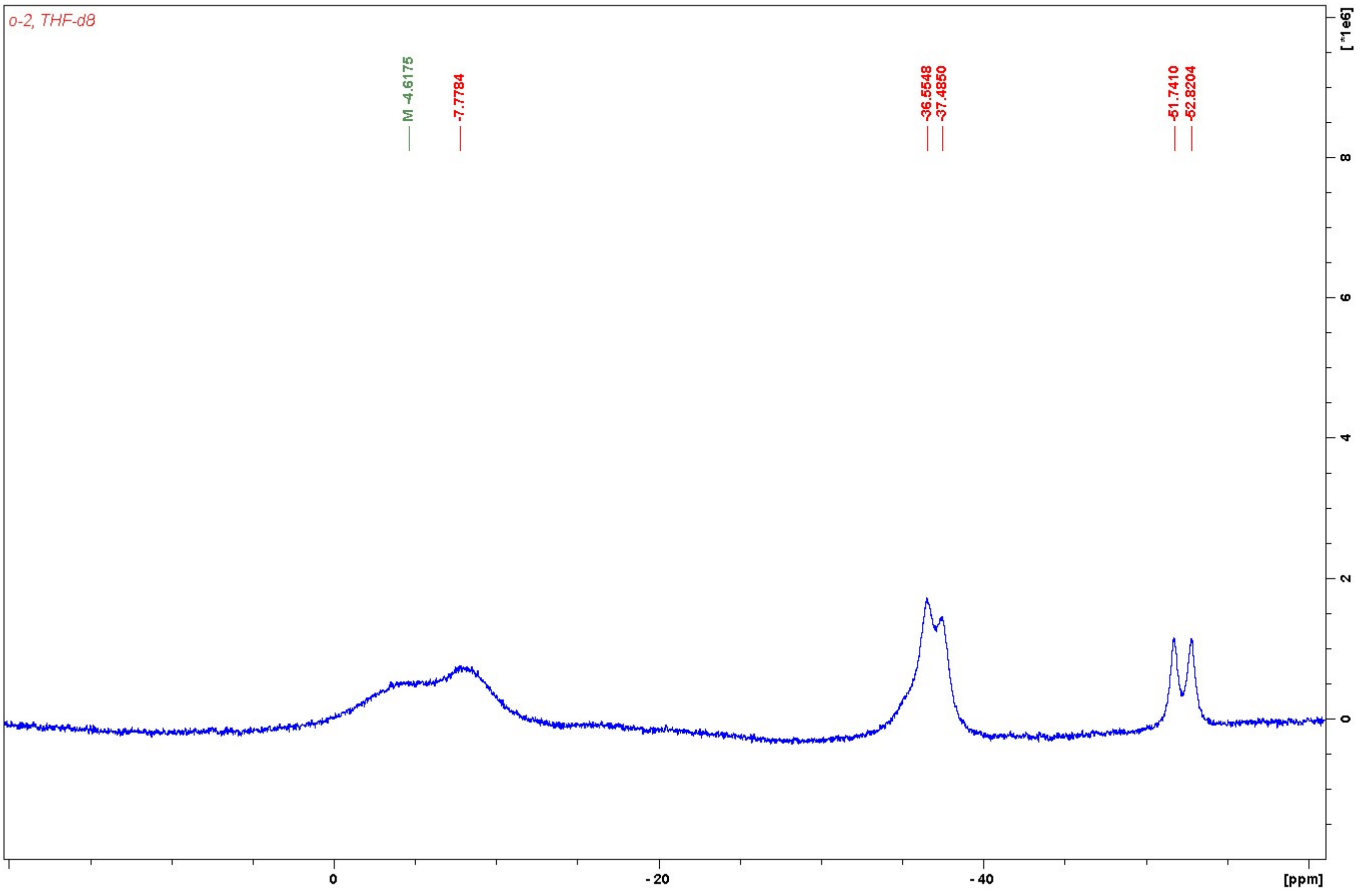

Supplementary Figure 7. ^{11}B NMR spectrum of *o*-2.

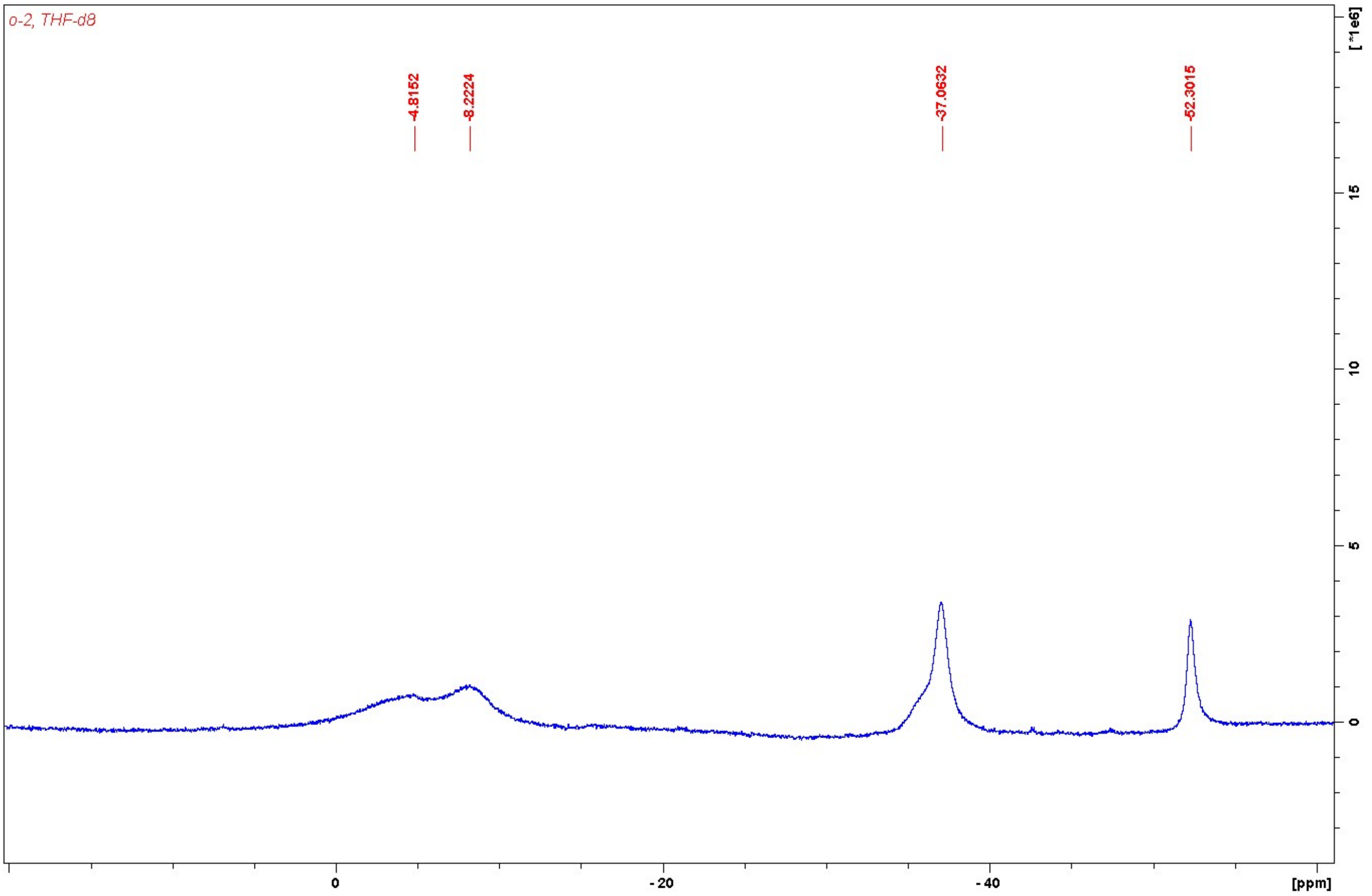

Supplementary Figure 8. $^{11}B\{^1H\}$ **NMR spectrum of *o*-2.**

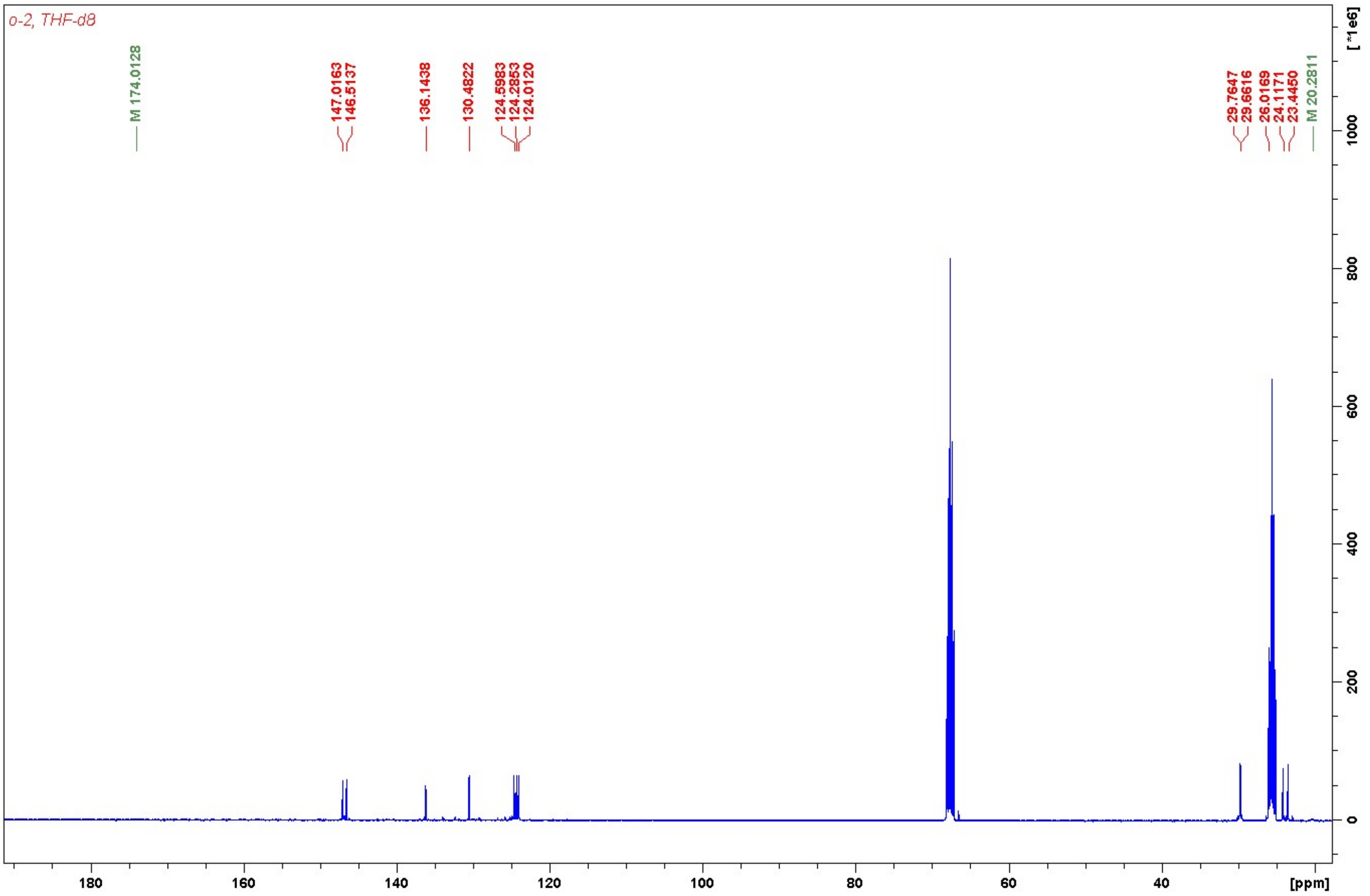

Supplementary Figure 9. $^{13}C\{^1H\}$ **NMR spectrum of *o*-2.**

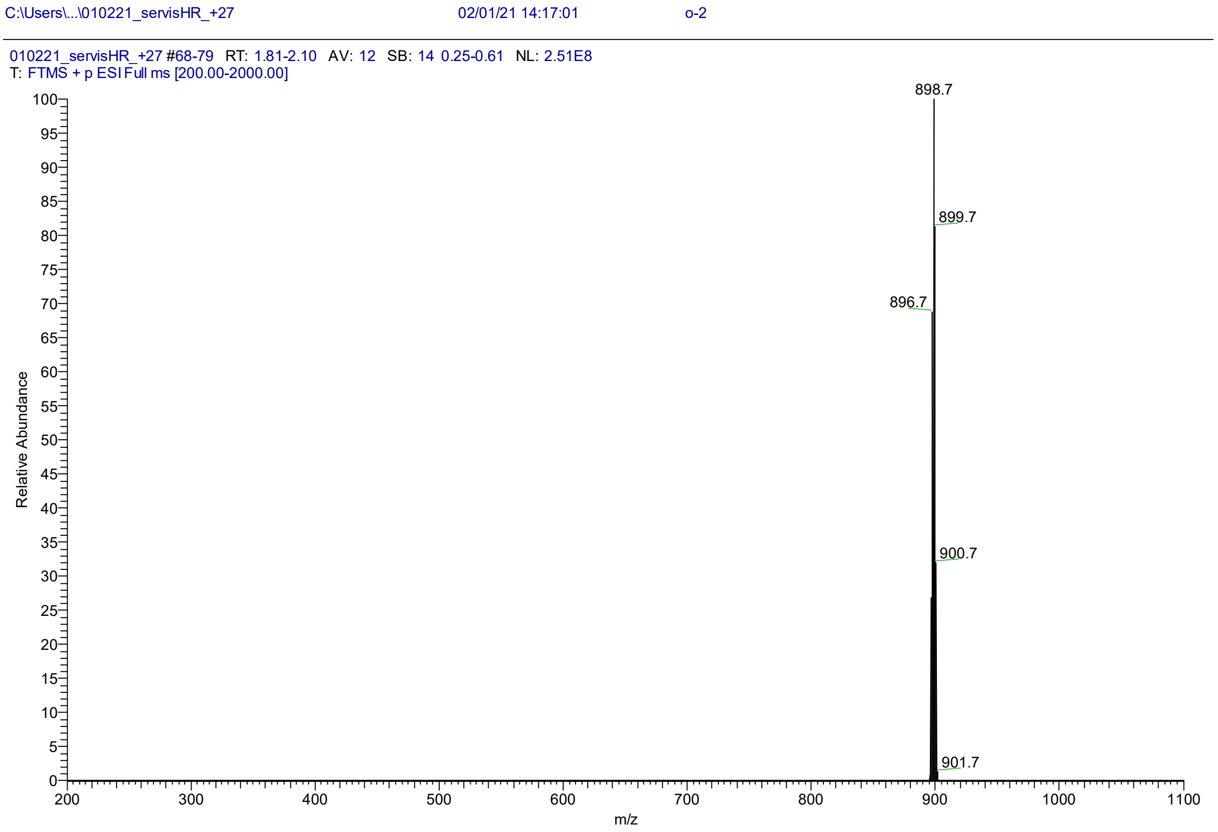

Supplementary Figure 10. MS spectrum (ESI+) of o-2: $[M + H]^+$, *m/z* 899.7. HRMS (ESI+): *m/z* calculated for $C_{56}H_{83}N_4B_8^+$ 899.73567, found 899.73535 (-0.36 ppm).

Synthesis of 6,9-NHC^{Dip}$_2$-5,7-$C_2B_8H_{10}$ (*m*-2)

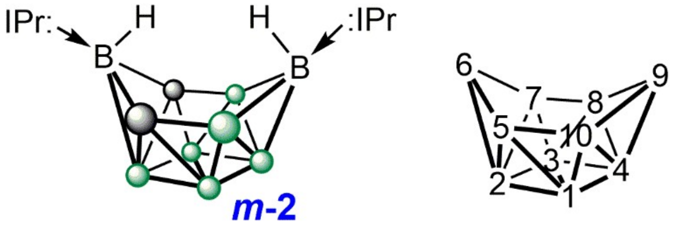

Solution of NHC^{Dip} (425 mg, 1.09 mmol) in diethyl ether (10 mL) was added dropwise to a stirred solution of *closo-m*-$C_2B_8H_{10}$ (66 mg, 0.55 mmol) in diethyl ether (5 mL) at room temperature. The turbid reaction mixture was filtrated and the slightly yellow solution was left for crystallization for 5 hours. The crude product was washed with hexane (15 mL) to remove the excess NHC^{Dip}. The slightly yellow powder of ***m*-2** was dried in vacuo. Colorless single crystals of ***m*-2** were decanted and dried in vacuo. Yield 256 mg, 52 %. **Dp** 144 °C. **Anal. Calc**. for $C_{56}H_{82}B_8N_4$ (897.77): C 74.9, H 9.2, N 6.2; found C 74.9, H 9.3, N 6.3. ^{1}**H NMR** (25 °C, thf-d$_8$, 500 MHz): δ = -0.58 (s broad, 2H, BC*H*), 1.04 (d, $^3J(^1H\text{-}^1H)$ = 6.7 Hz, 12H, CH(C*H*$_3$)$_2$), 1.05 (d, $^3J(^1H\text{-}^1H)$ = 6.7 Hz, 12H, CH(C*H*$_3$)$_2$), 1.18 (d, $^3J(^1H\text{-}^1H)$ = 6.8 Hz, 12H, CH(C*H*$_3$)$_2$), 1.20 (d, $^3J(^1H\text{-}^1H)$ = 6.8 Hz, 12H, CH(C*H*$_3$)$_2$), 2.53 (sept, $^3J(^1H\text{-}^1H)$ = 6.7 Hz, 4H, C*H*(CH$_3$)$_2$), 2.80 (sept, $^3J(^1H\text{-}^1H)$ = 6.7 Hz, 4H, CH(C*H*$_3$)$_2$), 6.89 (s, 2H, C*H*=C*H*), 7.11 (d, $^3J(^1H\text{-}^1H)$ = 7.8 Hz, 4H, *m*-Ar*H*), 7.16 (s, 2H, C*H*=C*H*), 7.19 (d, $^3J(^1H\text{-}^1H)$ = 7.8 Hz, 4H, *m*-Ar*H*), 7.25 (t, $^3J(^1H\text{-}^1H)$ = 7.8 Hz, 2H, *p*-Ar*H*), 7.36 (t, $^3J(^1H\text{-}^1H)$ = 7.8 Hz, 2H, *p*-Ar*H*) ppm. ^{11}**B NMR** (25 °C, thf-d$_8$, 160.42 MHz): δ = -39.8 (s broad, 2B, B1,3), -37.3 (s broad, 1B, B9), -28.9 (s broad, 1B, B6), -15.1 (s broad, 1B, B4), -4.8 (s broad, 2B, B8,10), 0.3 (s broad, 1B, B2) ppm. ^{13}**C{**^{1}**H} NMR** (25 °C, thf-d$_8$, 125.76 Hz): δ = 18.2 (s broad, B*C*H), 24.0, 24.1, 26.0 (s, CH(*C*H$_3$)$_2$), 29.5, 29.7 (s, *C*H(CH$_3$)$_2$), 123.6, 124.2 (s, *C*H=*C*H), 124.7, 124.7 (s, *m*-Ar*C*), 129.7, 130.9 (s, *p*-Ar*C*), 135.6, 137.7 (s, *ipso*-Ar*C*), 146.62, 147.65 (s, *o*-Ar*C*), 171.4, 175.4 (s very broad, N*C*N) ppm.

Spectroscopic characterization of 6,9-NHC$^{Dip}_2$-5,7-$C_2B_8H_{10}$ (*m*-2)

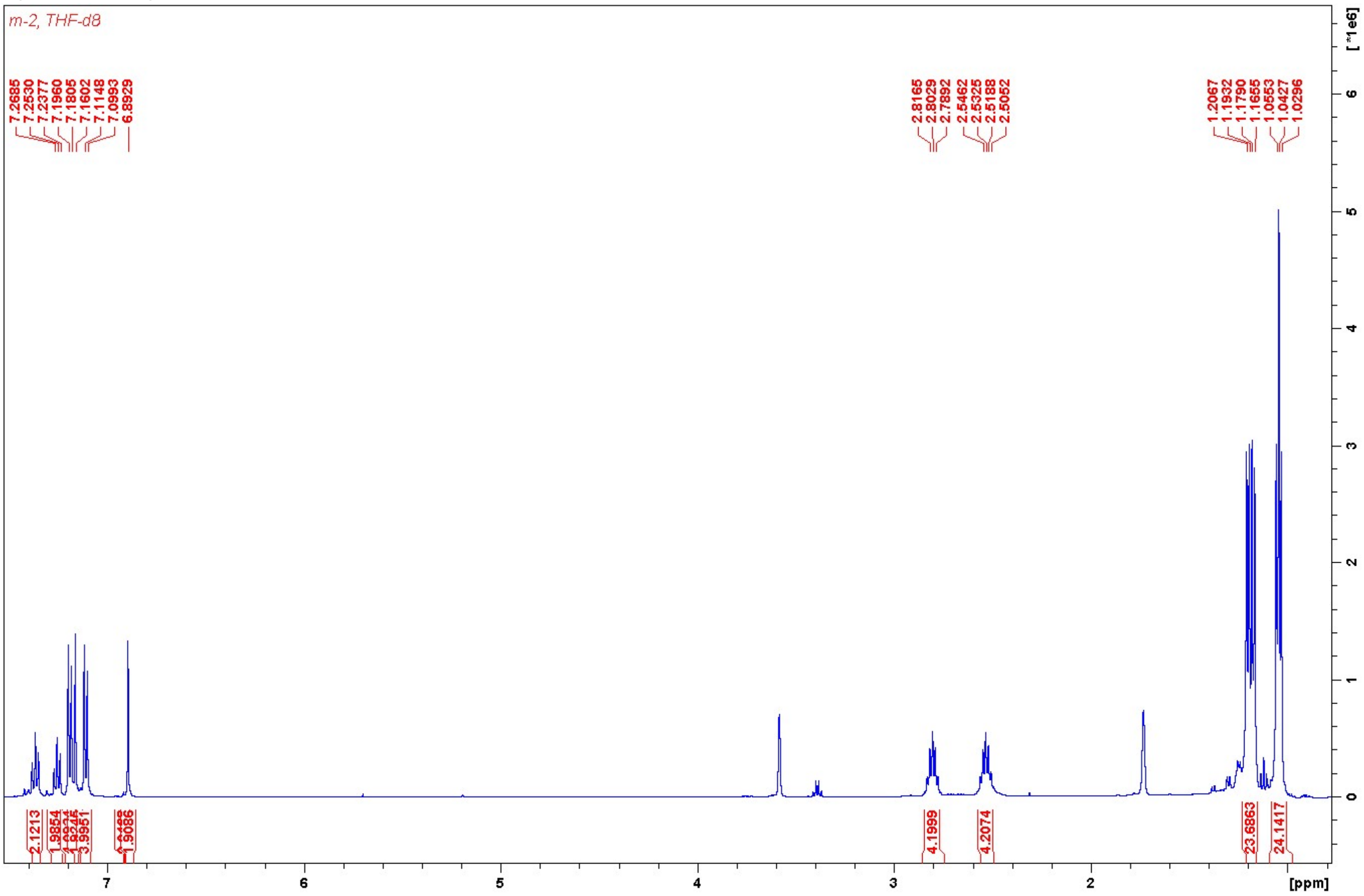

Supplementary Figure 11. ^{1}**H NMR spectrum of *m*-2.**

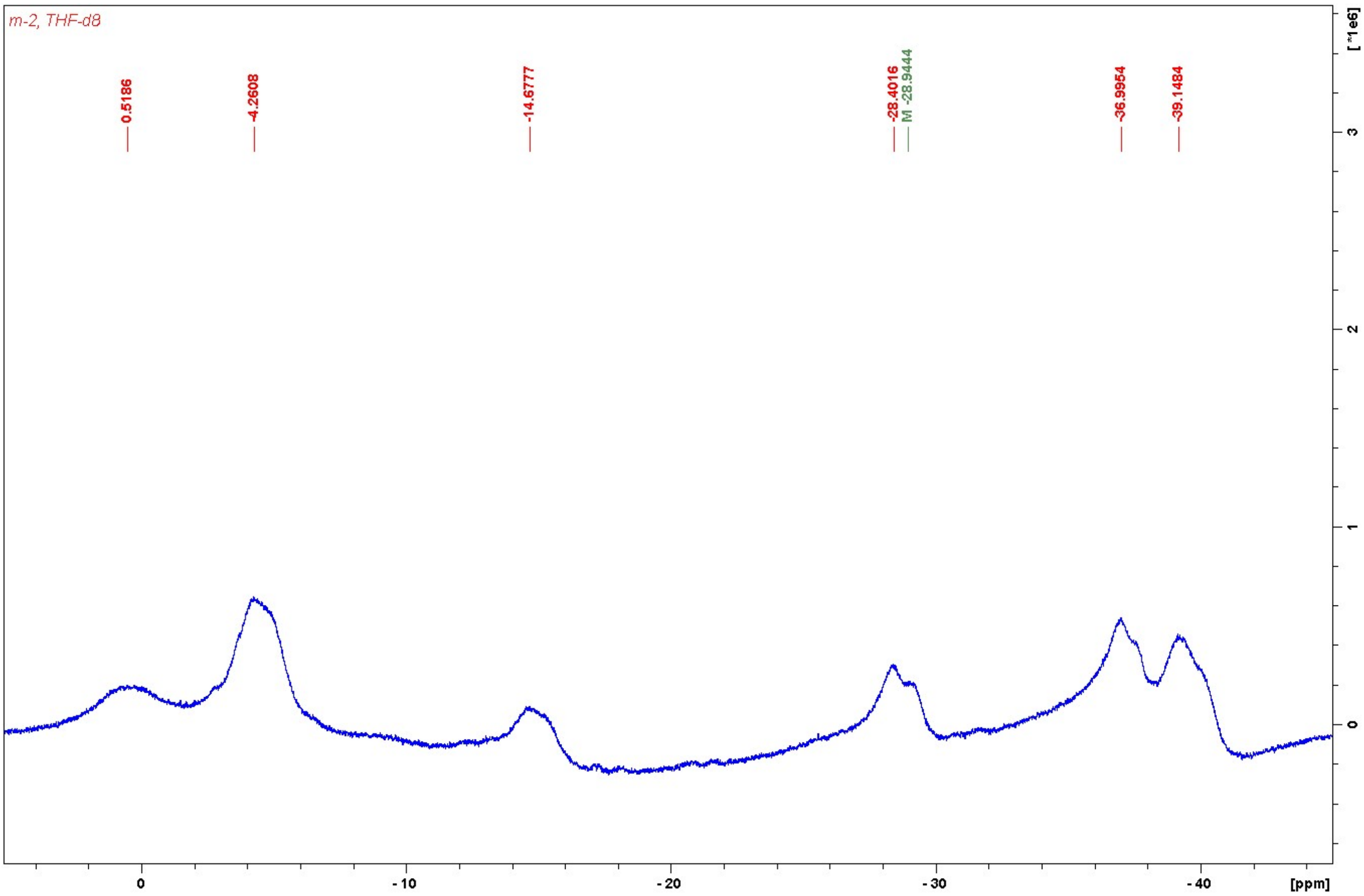

Supplementary Figure 12. ^{11}**B NMR spectrum of *m*-2.**

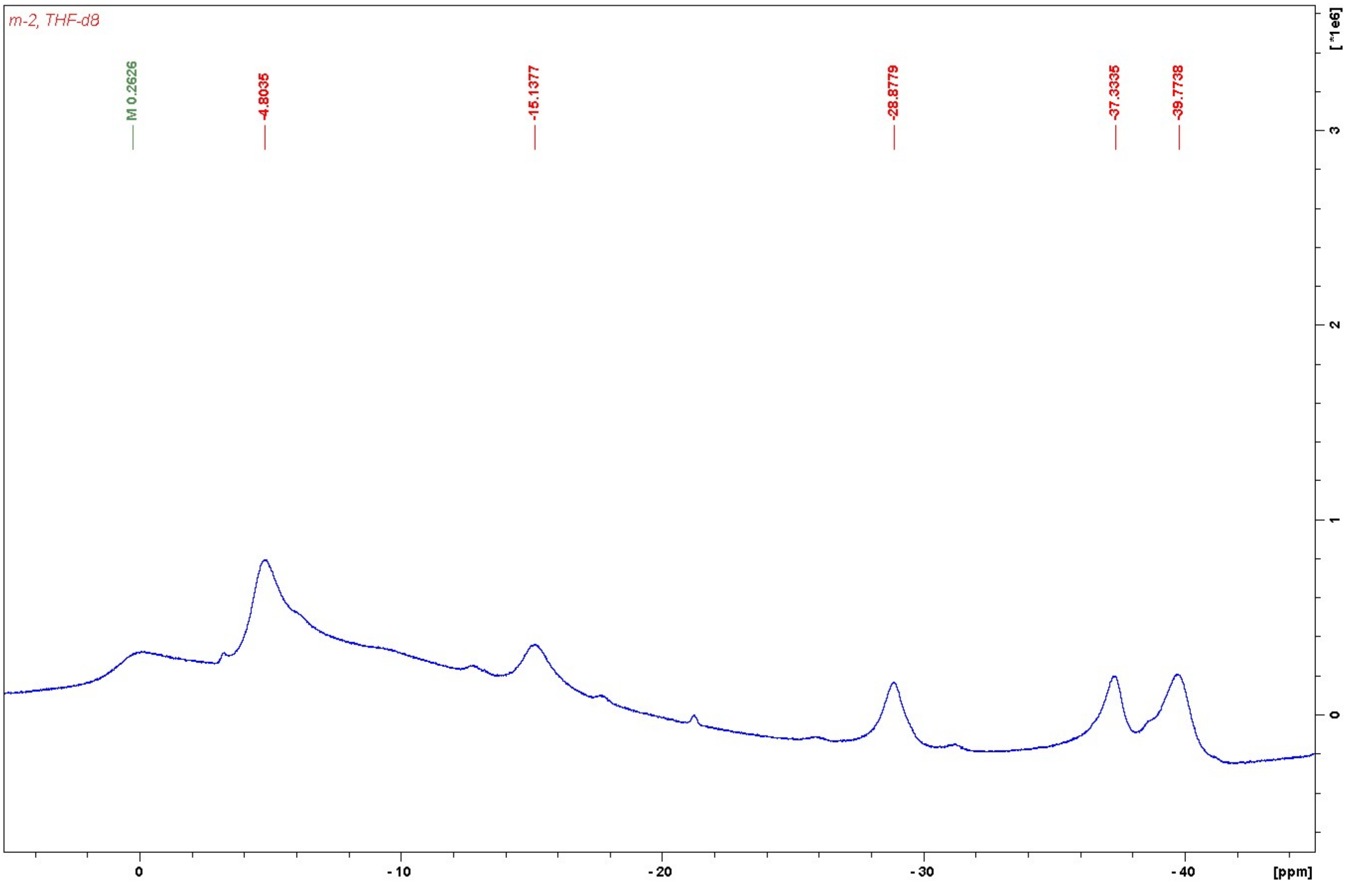

Supplementary Figure 13. $^{11}B\{^1H\}$ **NMR spectrum of *m*-2.**

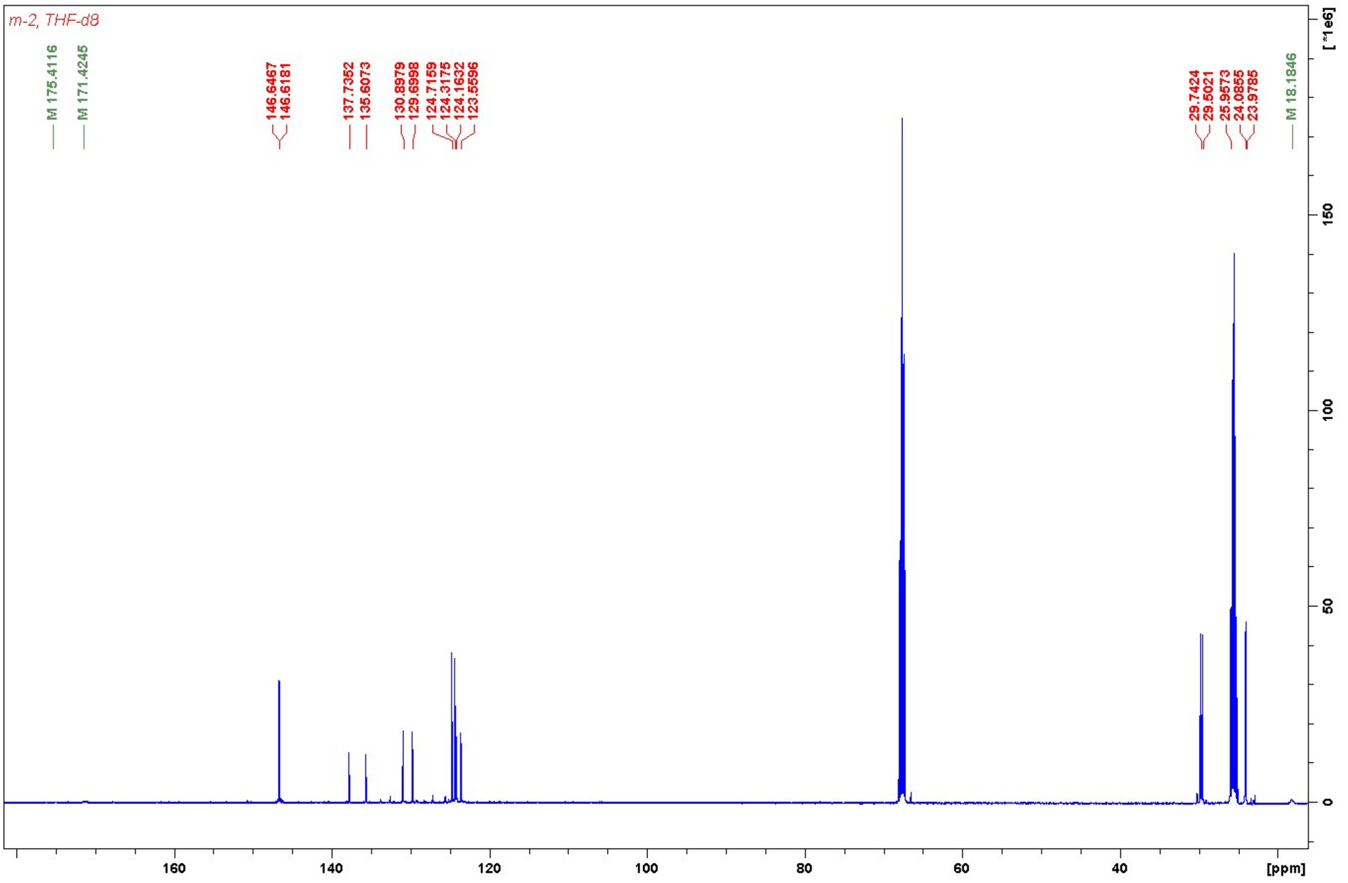

Supplementary Figure 14. $^{13}C\{^1H\}$ **NMR spectrum of *m*-2.**

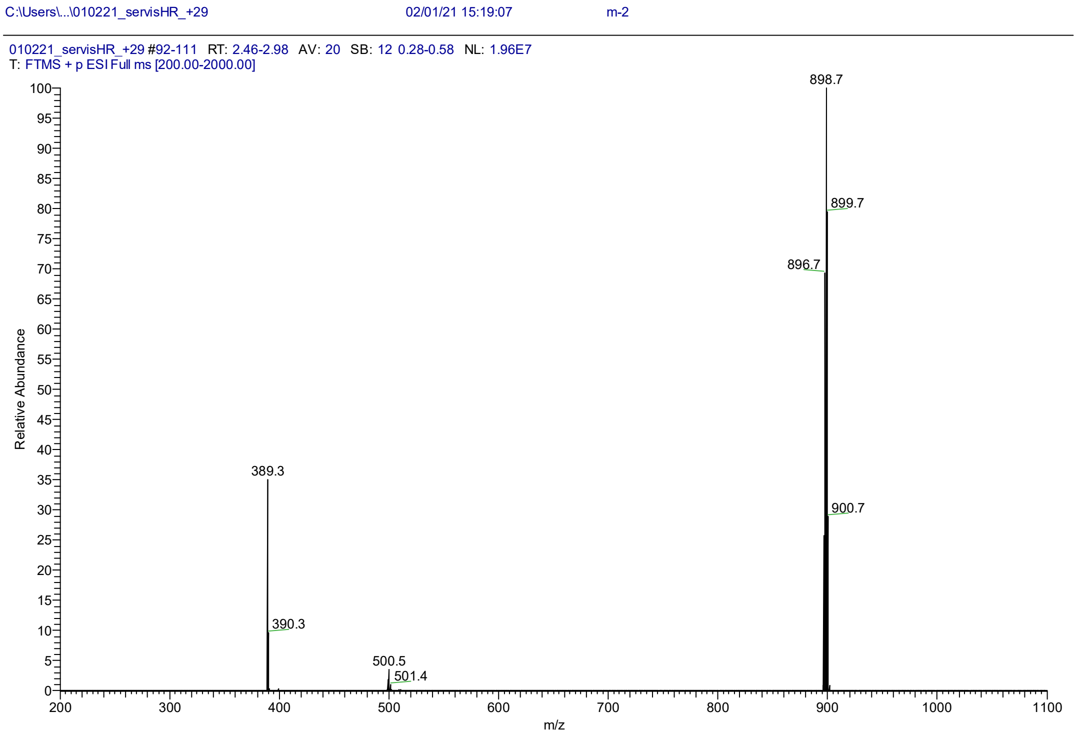

Supplementary Figure 15. MS spectrum (ESI+) of *m*-2: $[:IPr + H]^+$, *m/z* 389.3; $[M + H]^+$, *m/z* 899.7. HRMS (ESI+): *m/z* calculated for $C_{56}H_{83}N_4B_8^+$ 899.73567, found 899.73667 (1.11 ppm).

Supplementary Table 2. Crystal data and structure refinement for *m*-2.

Chemical formula	$C_{56}H_{82}B_8N_4$
M_r	897.73
Crystal system, space group	Monoclinic, $P2_1/n$
Temperature (K)	150
a, *b*, *c* (Å)	10.6749 (5), 19.6096 (11), 26.8512 (13)
β (°)	93.258 (2)
V (Å^3)	5611.7 (5)
Z	4
Radiation type	Mo *K*α
μ (mm^{-1})	0.06
Crystal size (mm)	0.59 × 0.36 × 0.21
Data collection	
Diffractometer	Bruker D8 - Venture
Absorption correction	Multi-scan<br>*SADABS2016*/2 - Bruker AXS area detector scaling and absorption correction
T_{min}, T_{max}	0.680, 0.746
No. of measured, independent and observed [$I > 2\sigma(I)$] reflections	141173, 12938, 9763
R_{int}	0.075
$(\sin \theta/\lambda)_{max}$ (Å^{-1})	0.652
Refinement	
$R[F^2 > 2\sigma(F^2)]$, $wR(F^2)$, S	0.070, 0.186, 1.07
No. of reflections	12938
No. of parameters	646
No. of restraints	627
H-atom treatment	H atoms treated by a mixture of independent and constrained refinement
$\Delta\rho_{max}$, $\Delta\rho_{min}$ (e Å^{-3})	0.63, −0.59

Synthesis of 6,9-NHC$^{Dip}_{2}$-5,8-$C_2B_8H_{10}$ (*p*-2)

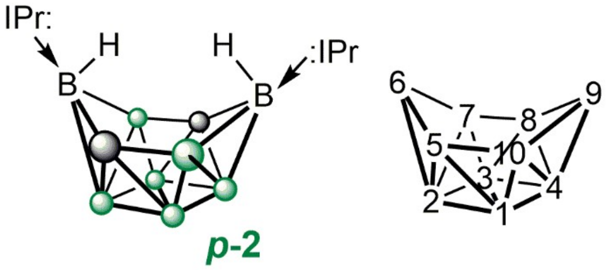

***p*-2**

Solution of NHC^{Dip} (509 mg, 1.31 mmol) in diethyl ether (10 mL) was added at once to solution of *closo*-*p*-$C_2B_8H_{10}$ (79 mg, 0.66 mmol) in diethyl ether (5 mL) at room temperature. The reaction mixture was left for crystallization for four days. Slightly yellow single crystals of ***p*-2** were decanted and dried in vacuo. Yield 500 mg, 85 %. **Dp** 170 °C. **Anal. Calc**. for $C_{56}H_{82}B_8N_4$ (897.77): C 74.9, H 9.2, N 6.2; found C 75.0, H 9.3, N 6.1. **^{1}H NMR** (25 °C, thf-d$_8$, 500 MHz): δ = -0.75 (s broad, 2H, BC*H*), 1.02 (d, $^{3}J(^{1}H-^{1}H)$ = 6.9 Hz, 12H, CH(C*H*$_3$)$_2$), 1.07 (d, $^{3}J(^{1}H-^{1}H)$ = 6.9 Hz, 12H, CH(C*H*$_3$)$_2$), 1.19 (d, $^{3}J(^{1}H-^{1}H)$ = 6.8 Hz, 12H, CH(C*H*$_3$)$_2$), 1.28 (d, $^{3}J(^{1}H-^{1}H)$ = 6.8 Hz, 12H, CH(C*H*$_3$)$_2$), 2.63 (sept, $^{3}J(^{1}H-^{1}H)$ = 6.7 Hz, 4H, C*H*(CH$_3$)$_2$), 2.71 (sept, $^{3}J(^{1}H-^{1}H)$ = 6.7 Hz, 4H, CH(C*H*$_3$)$_2$), 7.04 (s, 4H, C*H*=C*H*), 7.12 (d, $^{3}J(^{1}H-^{1}H)$ = 7.8 Hz, 4H, *m*-Ar*H*), 7.14 (d, $^{3}J(^{1}H-^{1}H)$ = 8.0 Hz, 4H, *m*-Ar*H*), 7.31 (t, $^{3}J(^{1}H-^{1}H)$ = 8.0 Hz, 4H, *p*-Ar*H*) ppm. **^{11}B NMR** (25 °C, thf-d$_8$, 160.42 MHz): δ = -44.7 (d, $^{1}J(^{1}H-^{11}B)$ = 129 Hz, 2B, B1,3), -36.4 (d, $^{1}J(^{1}H-^{11}B)$ = 141 Hz, 2B, B6,9), -9.9 (s broad, 2B, B7,10), -7.0 (s broad, 2B, B2,4) ppm. **^{13}C{^{1}H} NMR** (25 °C, thf-d$_8$, 125.76 Hz): δ = 22.2 (s broad, B*C*H), 23.6, 24.1, 25.8, 25.9 (s, CH(*C*H$_3$)$_2$), 29.6, 29.8 (s, *C*H(CH$_3$)$_2$), 123.9 (s, *C*H=*C*H), 124.3, 124.5 (s, *m*-Ar*C*), 130.4 (s, *p*-Ar*C*), 136.2 (s, *ipso*-Ar*C*), 146.2, 147.2 (s, *o*-Ar*C*), 173.8 (s very broad, N*C*N) ppm.

Spectroscopic characterization of 6,9-NHC$^{Dip}_{2}$-5,8-$C_2B_8H_{10}$ (*p*-2)

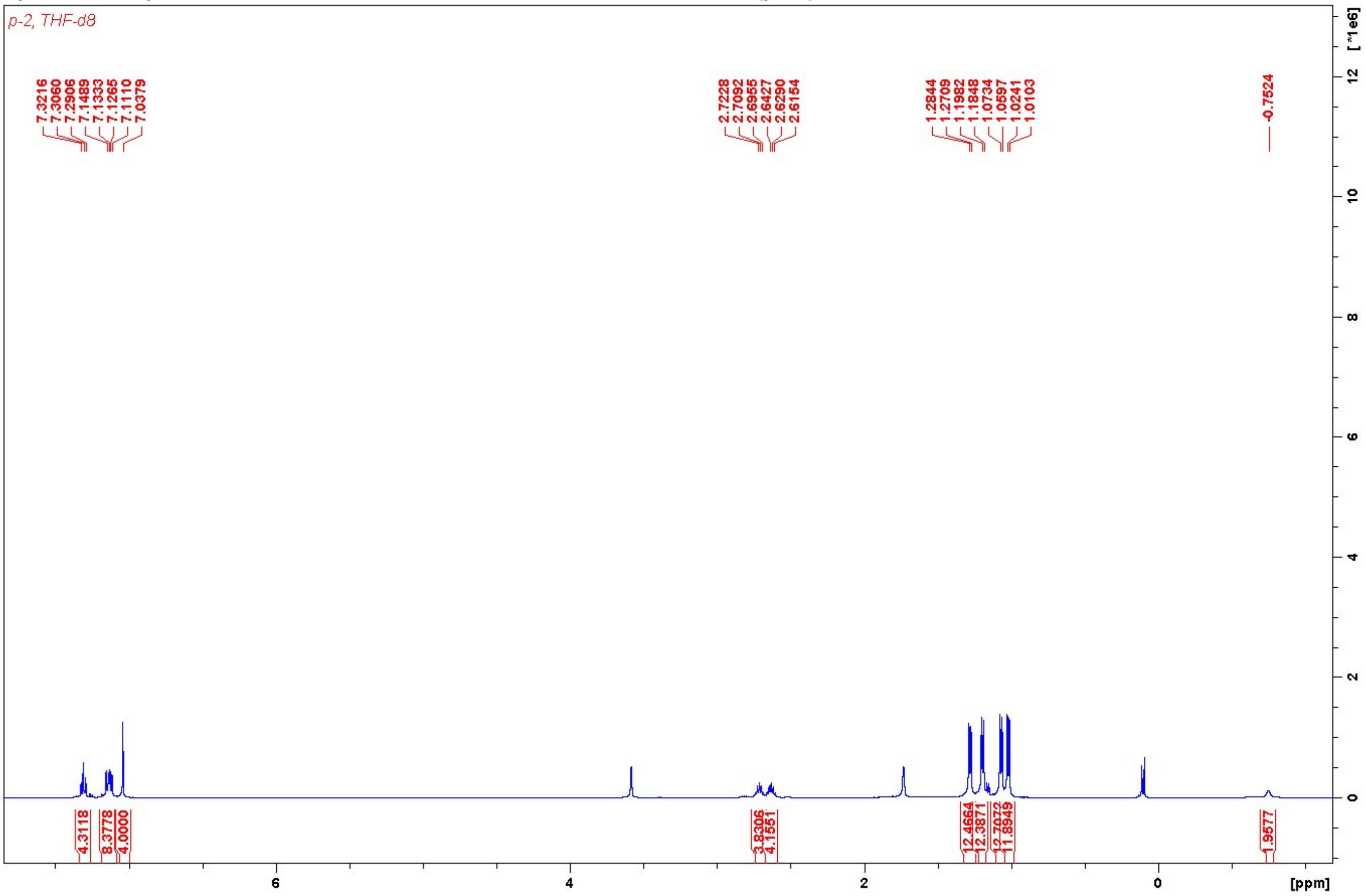

Supplementary Figure 16. ^{1}H NMR spectrum of *p*-2.

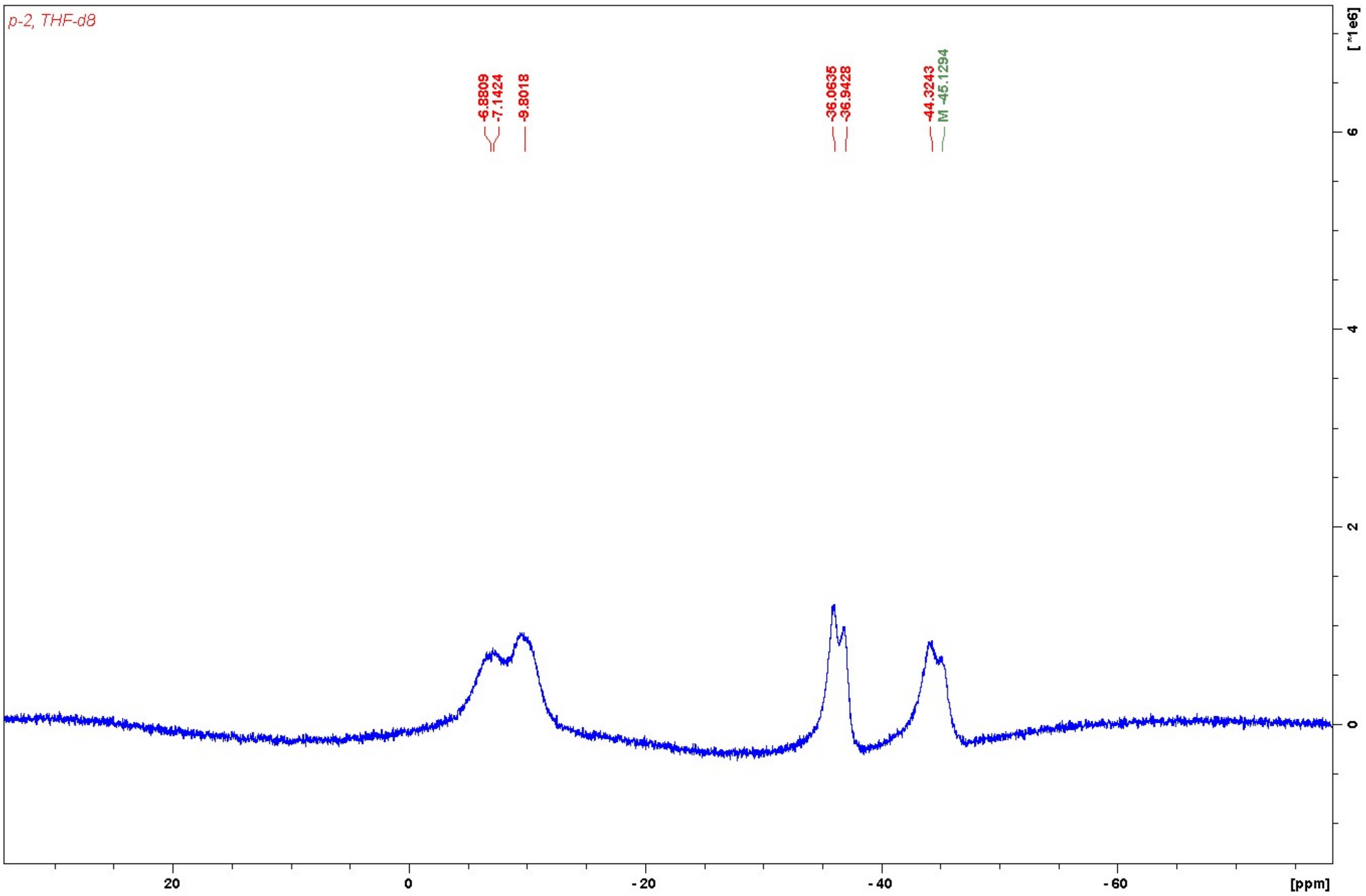

Supplementary Figure 17. ^{11}**B NMR spectrum of *p*-2.**

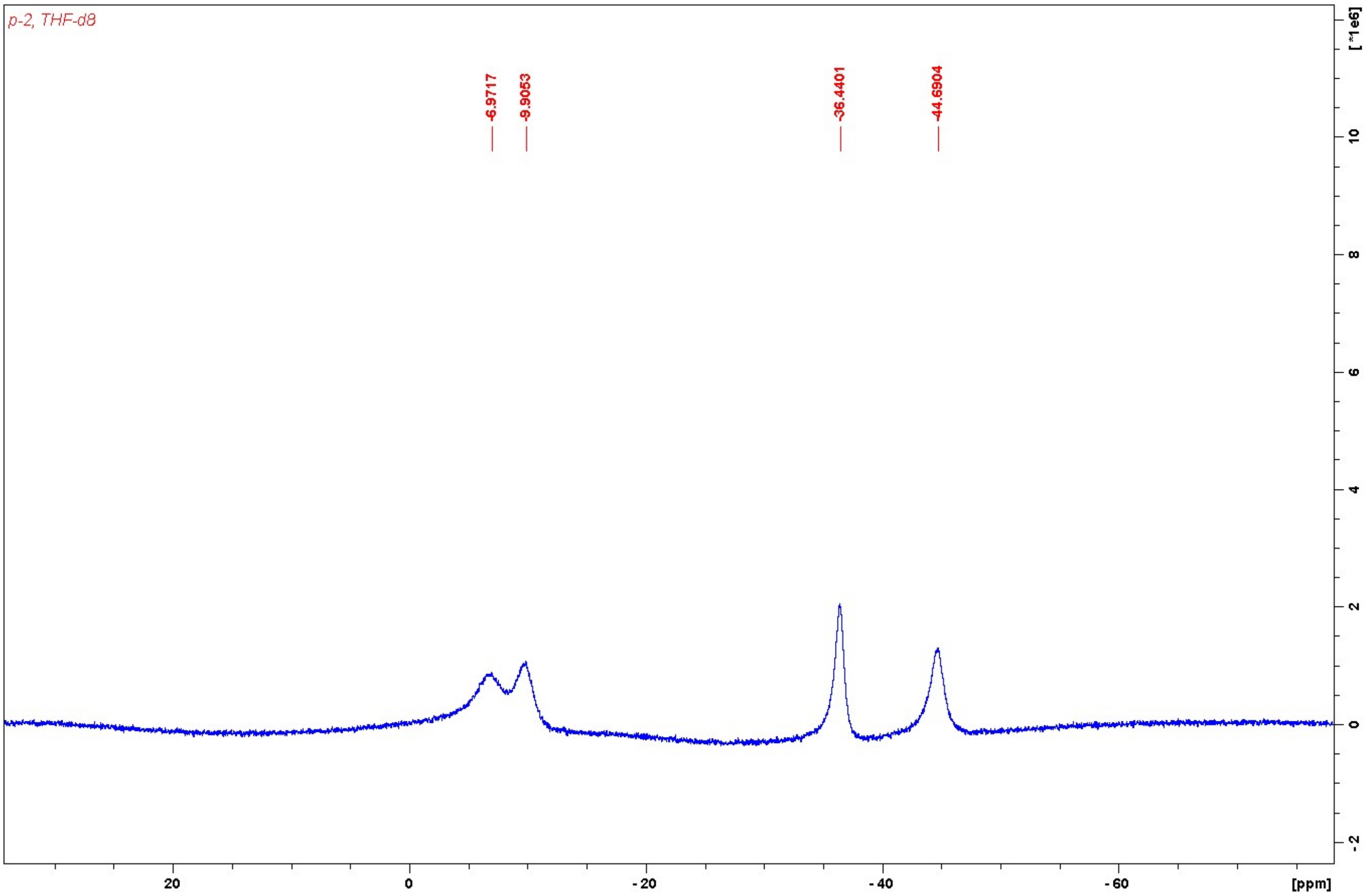

Supplementary Figure 18. ^{11}**B{**^{1}**H} NMR spectrum of *p*-2.**

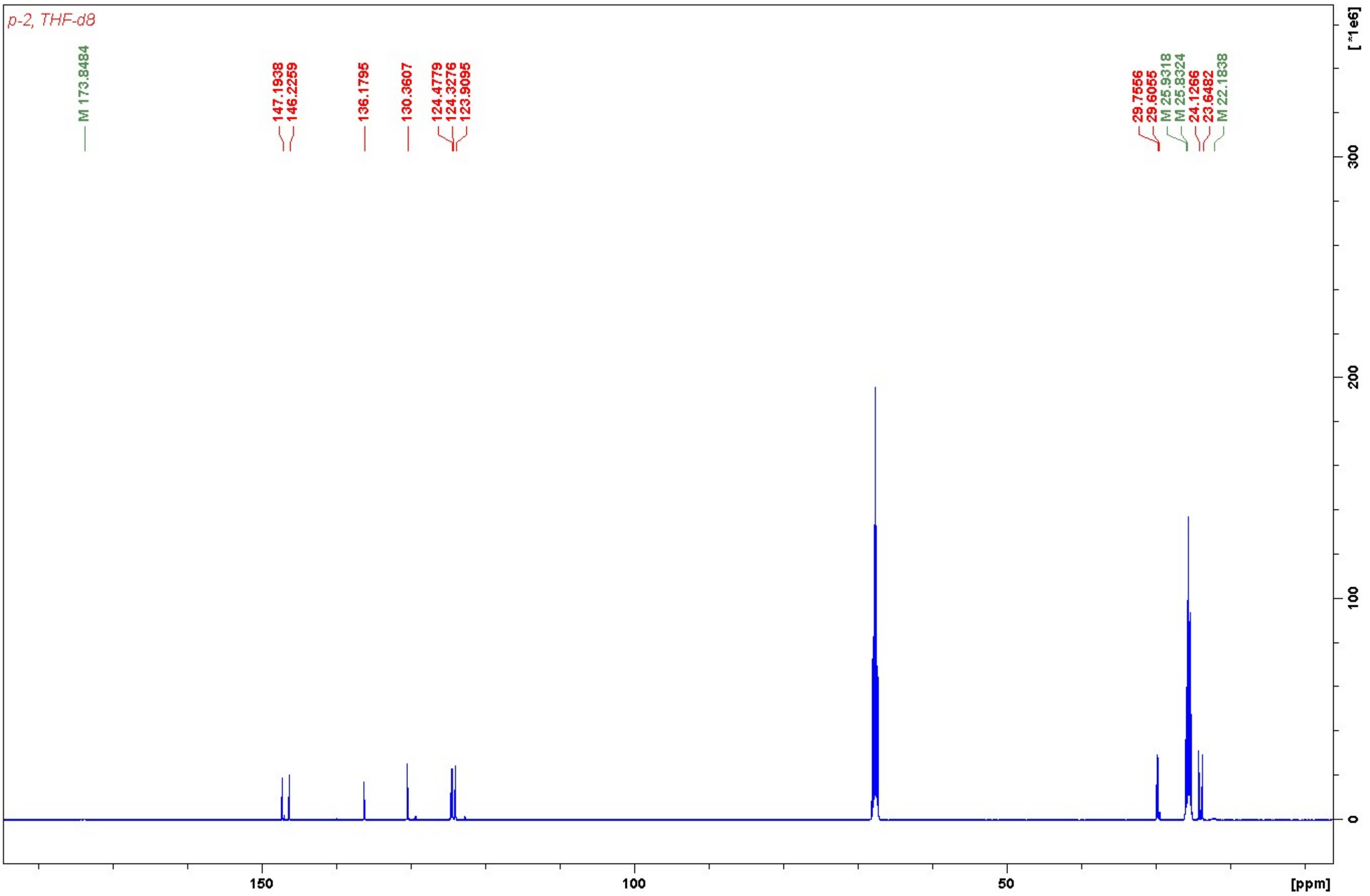

Supplementary Figure 19. **$^{13}C\{^{1}H\}$ NMR spectrum of *p*-2.**

Supplementary Table 3. Crystal data and structure refinement for *p*-2.

Chemical formula	$C_{60}H_{92}B_8N_4O$
M_r	971.85
Crystal system, space group	Monoclinic, *Cc*
Temperature (K)	150
a, *b*, *c* (Å)	54.586 (2), 10.3337 (4), 21.6874 (8)
β (°)	93.154 (3)
V (Å^3)	12214.8 (8)
Z	8
Radiation type	Cu *K*α
μ (mm^{-1})	0.44
Crystal size (mm)	0.73 × 0.26 × 0.25
Data collection	
Diffractometer	Bruker D8 - Venture
Absorption correction	Multi-scan<br>*SADABS* 2016/2: Krause, L., Herbst-Irmer, R., Sheldrick G.M. & Stalke D., J. Appl. Cryst. 48 (2015) 3-10
T_{min}, T_{max}	0.748, 0.845
No. of measured, independent and observed [$I > 2\sigma(I)$] reflections	79215, 17993, 15986
R_{int}	0.098
$(\sin \theta/\lambda)_{max}$ (Å^{-1})	0.638
Refinement	
$R[F^2 > 2\sigma(F^2)]$, $wR(F^2)$, S	0.079, 0.224, 1.03
No. of reflections	17993
No. of parameters	1422
No. of restraints	16
H-atom treatment	H atoms treated by a mixture of independent and constrained refinement
$\Delta\rho_{max}$, $\Delta\rho_{min}$ (e Å^{-3})	0.34, −0.34
Absolute structure	Flack x determined using 3339 quotients [(I+)-(I-)]/[(I+)+(I-)] (Parsons, Flack and Wagner, Acta Cryst. B69 (2013) 249-259). Refined as a 2-component twin.
Absolute structure parameter	−0.3 (3) – this value is meaningless as the crystal is weak anomalous scatterer

Synthesis of [6,9-NHC$^{Dip}_2$-5,10-$C_2B_8H_{11}$]$^+$Cl$^-$ (*o*-2a)

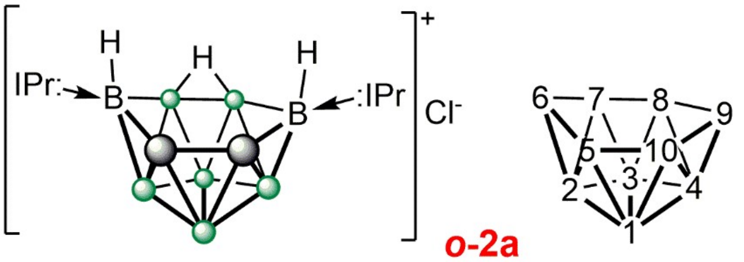

Solution of hydrogen chloride (0.235 mL, 3M solution in CPME, 0.71 mmol) was added to a stirred solution of ***o*-2** (211 mg, 0.24 mmol) in toluene (10 mL) at 0 °C. The resulting suspension was warmed to room temperature and filtrated. The colourless powder of ***o*-2a** was dried in vacuo. Yield 204 mg, 93 %. **Dp** 228 °C. **Anal. Calc**. for $C_{56}H_{83}B_8ClN_4$ (934.23): C 72.0, H 9.0, N 6.0; found C 72.0, H 9.2, N 5.9. **^{1}H NMR** (25 °C, dcm-d$_2$, 500 MHz): δ = 0.59 (s broad, 2H, BC*H*), 1.08 (d, $^3J(^1H-^1H)$ = 6.6 Hz, 12H, CH(C*H*$_3$)$_2$), 1.12 (d, $^3J(^1H-^1H)$ = 6.7 Hz, 12H, CH(C*H*$_3$)$_2$), 1.14 (d, $^3J(^1H-^1H)$ = 6.8 Hz, 12H, CH(C*H*$_3$)$_2$), 1.19 (d, $^3J(^1H-^1H)$ = 6.8 Hz, 12H, CH(C*H*$_3$)$_2$), 2.18 (sept, $^3J(^1H-^1H)$ = 6.8 Hz, 4H, C*H*(CH$_3$)$_2$), 2.38 (sept, $^3J(^1H-^1H)$ = 6.7 Hz, 4H, CH(C*H*$_3$)$_2$), 7.18 (s, 4H, C*H*=C*H*), 7.27 (d, $^3J(^1H-^1H)$ = 7.8 Hz, 8H, *m*-Ar*H*), 7.55 (t, $^3J(^1H-^1H)$ = 7.8 Hz, 4H, *p*-Ar*H*) ppm. **^{11}B NMR** (25 °C, dcm-d$_2$, 160.42 MHz): δ = -54.7 (d, $^1J(^1H-^{11}B)$ = 148 Hz, 1B, B3), -31.7 (d, $^1J(^1H-^{11}B)$ = 109 Hz, 2B, B6,9), -22.6 (s broad, 1B, B1), -7.4 (s broad, 2B, B2,4), -4.7 (s broad, 2B, B7,8) ppm. **^{13}C{^{1}H} NMR** (25 °C, dcm-d$_2$, 125.76 Hz): δ = 22.6, 23.3, 25.8, 26.1 (s, CH(*C*H$_3$)$_2$), 26.4 (s broad, B*C*H), 29.4 (s, *C*H(CH$_3$)$_2$), 124.8 (s, *C*H=*C*H), 125.0, 125.2 (s, *m*-Ar*C*), 131.9 (s, *p*-Ar*C*), 132.7 (s, *ipso*-Ar*C*), 145.5, 146.0 (s, *o*-Ar*C*), 160.6 (s very broad, N*C*N) ppm.

Spectroscopic characterization of [6,9-NHC$^{Dip}_2$-5,10-$C_2B_8H_{11}$]$^+$Cl$^-$ (*o*-2a)

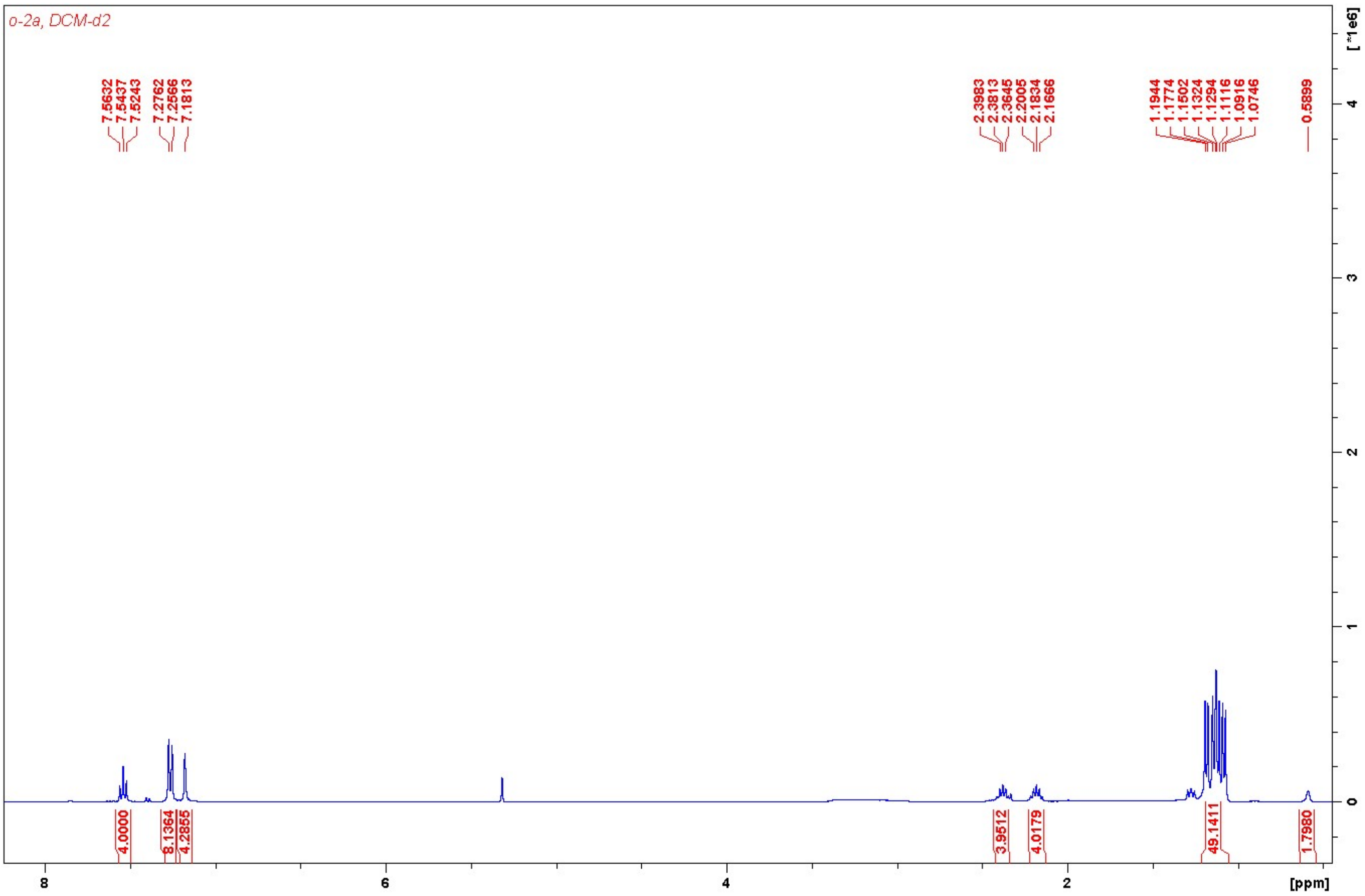

Supplementary Figure 20. ^{1}H NMR spectrum of *o*-2a.

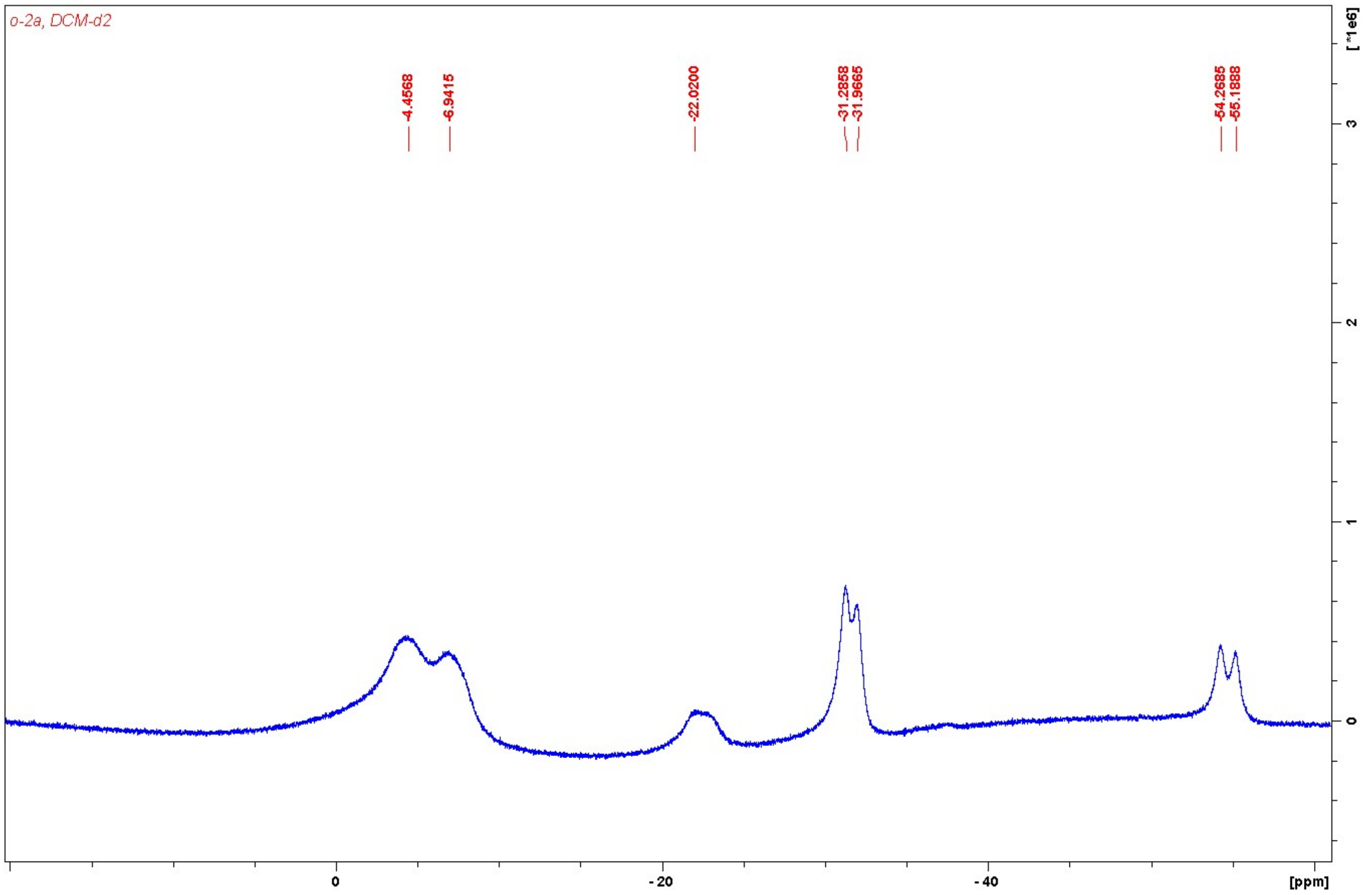

Supplementary Figure 21. ^{11}**B NMR spectrum of *o*-2a.**

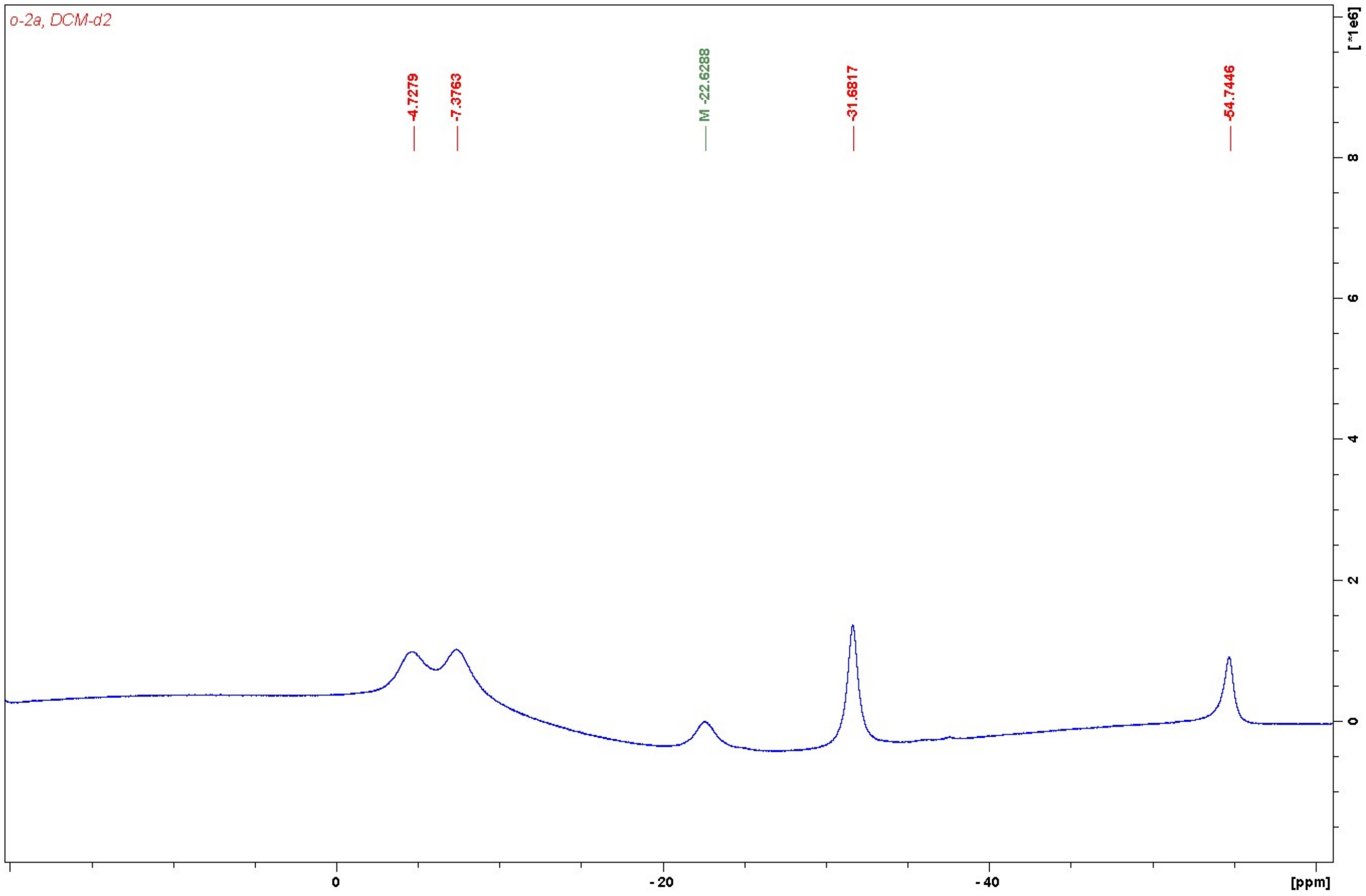

Supplementary Figure 22. ^{11}**B{**^{1}**H} NMR spectrum of *o*-2a.**

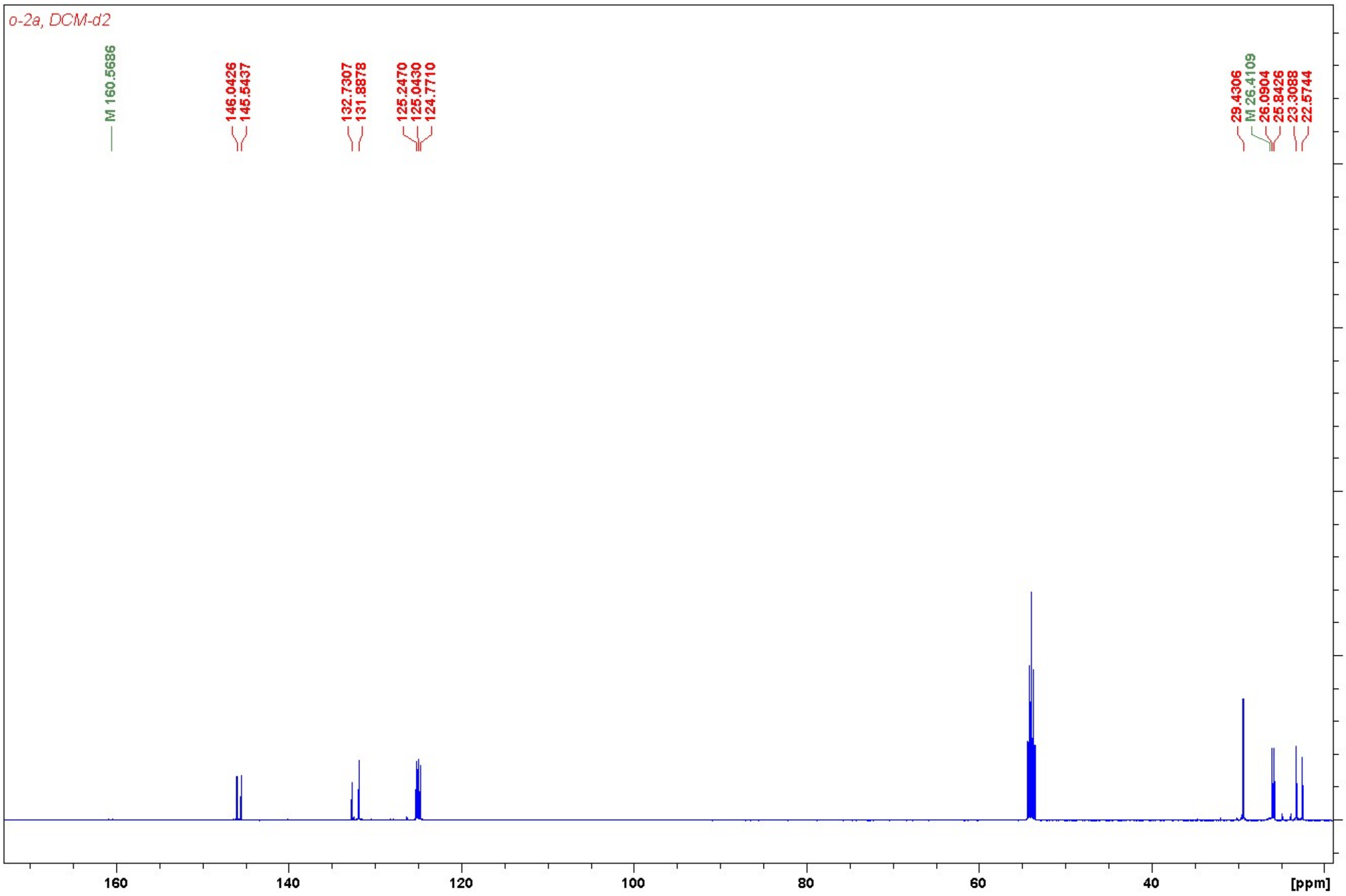

Supplementary Figure 23. $^{13}C\{^{1}H\}$ **NMR spectrum of *o*-2a.**

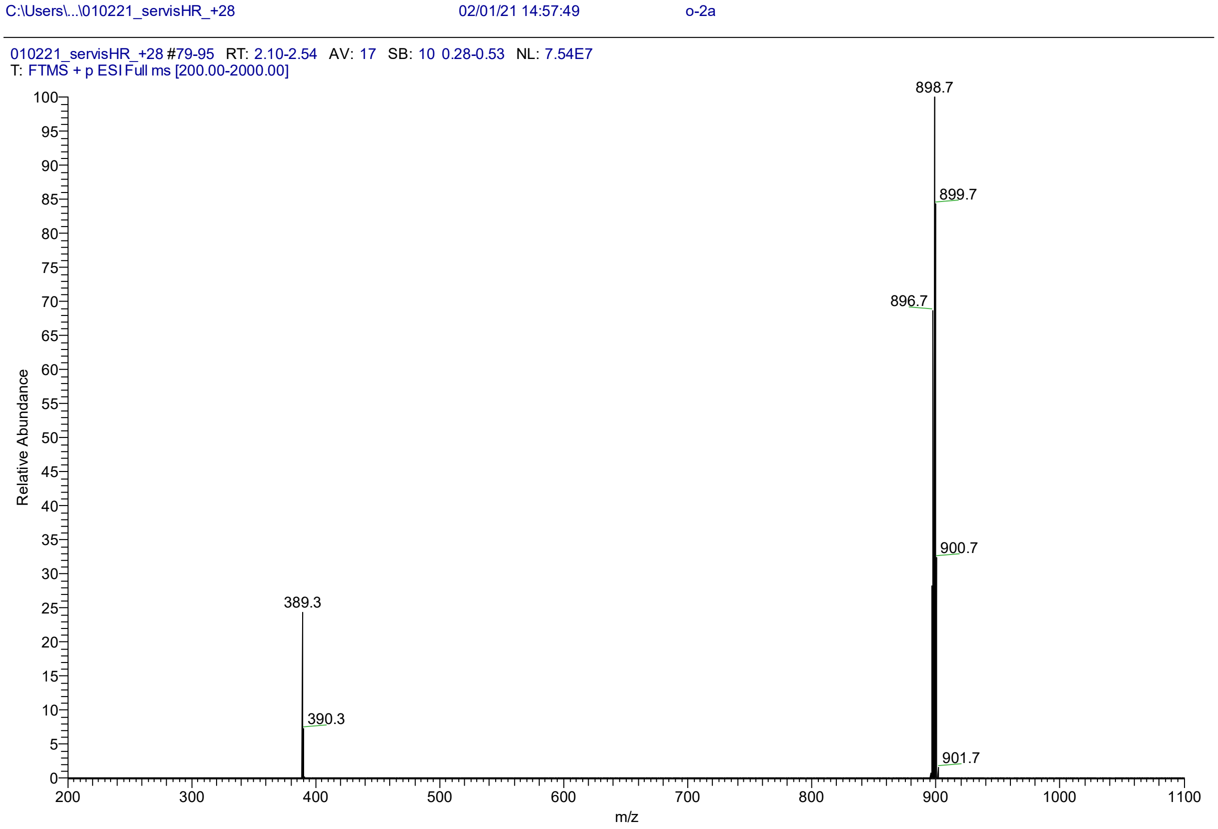

Supplementary Figure 24. MS spectrum (ESI+) of *o*-2a: [:IPr + H]$^+$, *m/z* 389.3; [M]$^+$, *m/z* 899.7. HRMS (ESI+): *m/z* calculated for $C_{56}H_{83}N_4B_8^+$ 899.73567, found 899.73695 (1.43 ppm).

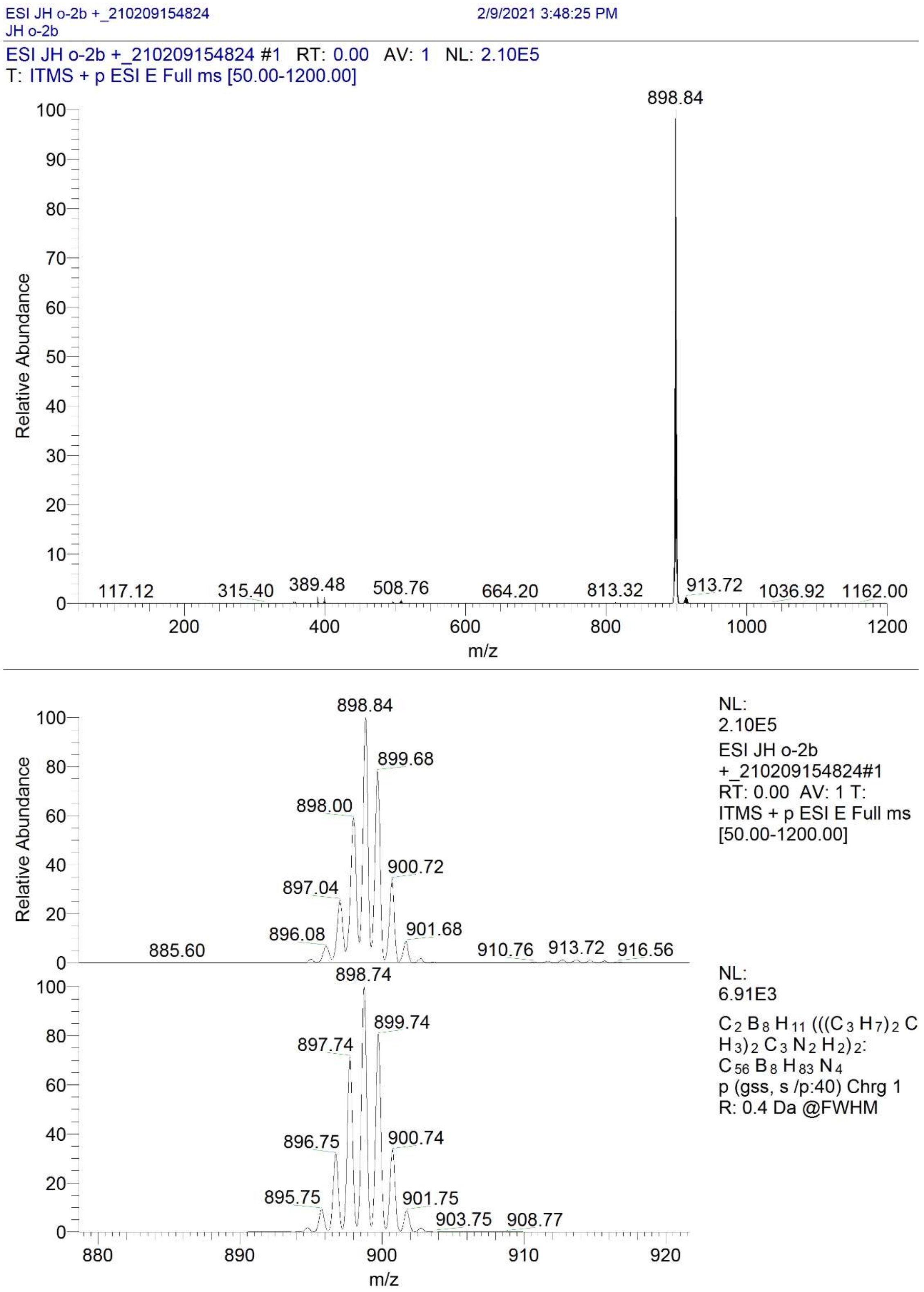

Supplementary Figure 25. MS spectrum (ESI+) of *o*-2a measured from the solution of D_2O: $[M]^+$, *m/z* 899.68.

Supplementary Table 4. Crystal data and structure refinement for *o*-2a.

Chemical formula	$C_{59}H_{89}B_8Cl_7N_4$
M_r	1188.97
Crystal system, space group	Monoclinic, $P2_1/n$
Temperature (K)	150
a, *b*, *c* (Å)	10.4219 (5), 27.8961 (12), 23.6604 (11)
β (°)	90.986 (2)
V (Å³)	6877.8 (5)
Z	4
Radiation type	Mo *K*α
μ (mm⁻¹)	0.33
Crystal size (mm)	0.20 × 0.20 × 0.20
Data collection	
Diffractometer	Bruker D8 - Venture
Absorption correction	Multi-scan<br>*SADABS2016*/2 - Bruker AXS area detector scaling and absorption correction
T_{min}, T_{max}	0.760, 0.958
No. of measured, independent and observed [$I > 2\sigma(I)$] reflections	68741, 13065, 9792
R_{int}	0.067
$(\sin \theta/\lambda)_{max}$ (Å⁻¹)	0.612
Refinement	
$R[F^2 > 2\sigma(F^2)]$, $wR(F^2)$, S	0.131, 0.346, 1.08
No. of reflections	13065
No. of parameters	812
No. of restraints	95
H-atom treatment	H atoms treated by a mixture of independent and constrained refinement
	$w = 1/[\sigma^2(F_o^2) + (0.1457P)^2 + 25.8362P]$<br>where $P = (F_o^2 + 2F_c^2)/3$
$\Delta\rho_{max}$, $\Delta\rho_{min}$ (e Å⁻³)	1.29, −0.87

Synthesis of [6,9-NHC$^{Dip}_2$-5,7-$C_2B_8H_{11}$]$^+$[HCl_2]$^-$ (*m*-2a)

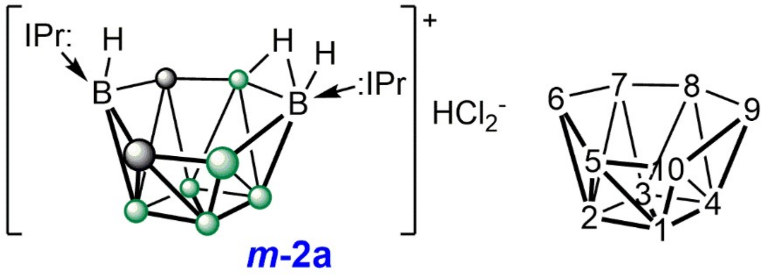

Solution of hydrogen chloride (0.134 mL, 3M solution in CPME, 0.40 mmol) was added to a stirred solution of ***m*-2** (120 mg, 0.13 mmol) in tetrahydrofuran (5 mL) at 0 °C. The resulting suspension was warmed to room temperature and filtrated. The colourless powder of ***m*-2a** was dried in vacuo. Yield 104 mg, 80 %. **Dp** 182 °C. **Anal. Calc**. for $C_{56}H_{84}B_8Cl_2N_4$ (970.69): C 69.3, H 8.7, N 5.8; found C 69.2, H 8.9, N 5.7**.** **^{1}H NMR** (25 °C, dcm-d$_2$, 500 MHz): δ = -0.29 (s broad, 2H, BC*H*), 1.08 (d, $^3J(^1H-^1H)$ = 6.8 Hz, 12H, CH(C*H*$_3$)$_2$), 1.11 (d, $^3J(^1H-^1H)$ = 6.8 Hz, 12H, CH(C*H*$_3$)$_2$), 1.12 (d, $^3J(^1H-^1H)$ = 6.8 Hz, 12H, CH(C*H*$_3$)$_2$), 1.20 (d, $^3J(^1H-^1H)$ = 6.8 Hz, 12H, CH(C*H*$_3$)$_2$), 2.30 (m, 8H, C*H*(CH$_3$)$_2$), 7.21 (s, 2H, C*H*=C*H*), 7.25 (d, $^3J(^1H-^1H)$ = 7.8 Hz, 8H, *m*-Ar*H*), 7.26 (d, $^3J(^1H-^1H)$ = 7.8 Hz, 4H, *m*-Ar*H*), 7.43 (s, 2H, C*H*=C*H*), 7.52 (m, 4H, *p*-Ar*H*) ppm. **^{11}B NMR** (25 °C, dcm-d$_2$, 160.42 MHz): δ = -37.5 (s broad, 1B, B9), -36.0 (s broad, 2B, B1,3), -33.7 (s broad, 1B, B4), -24.9 (s broad, 1B, B6), -5.0 (s broad, 2B, B8,10), -1.5 (s broad, 1B, B2) ppm. **^{13}C{^{1}H} NMR** (25 °C, dcm-d$_2$, 125.76 Hz): δ = 9.1 (s broad, B*C*H), 22.96, 23.04, 25.9, 26.0 (s, CH(*C*H$_3$)$_2$), 29.4, 29.5 (s, *C*H(CH$_3$)$_2$), 124.9 (s, *C*H=*C*H), 124.96, 125.03 (s, *m*-Ar*C*), 126.5 (s, *C*H=*C*H), 131.8, 132.1 (s, *p*-Ar*C*), 132.6, 132.8 (s, *ipso*-Ar*C*), 145.6 (s, *o*-Ar*C*), 155.5, 162.0 (s very broad, N*C*N) ppm.

Spectroscopic characterization of [6,9-NHC$^{Dip}_2$-5,7-$C_2B_8H_{11}$]$^+$[HCl_2]$^-$ (*m*-2a)

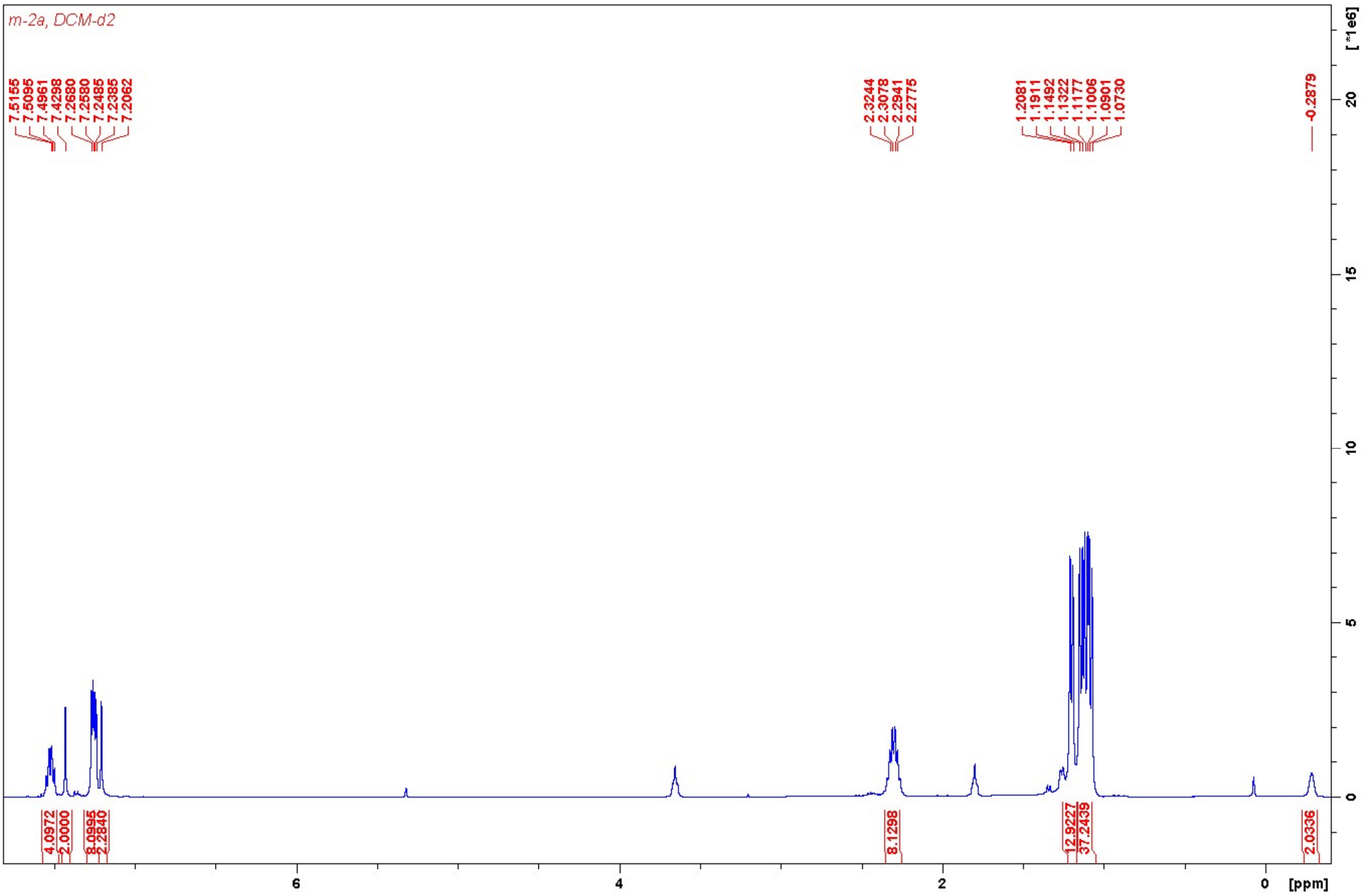

Supplementary Figure 26. ^{1}**H NMR spectrum of *m*-2a.**

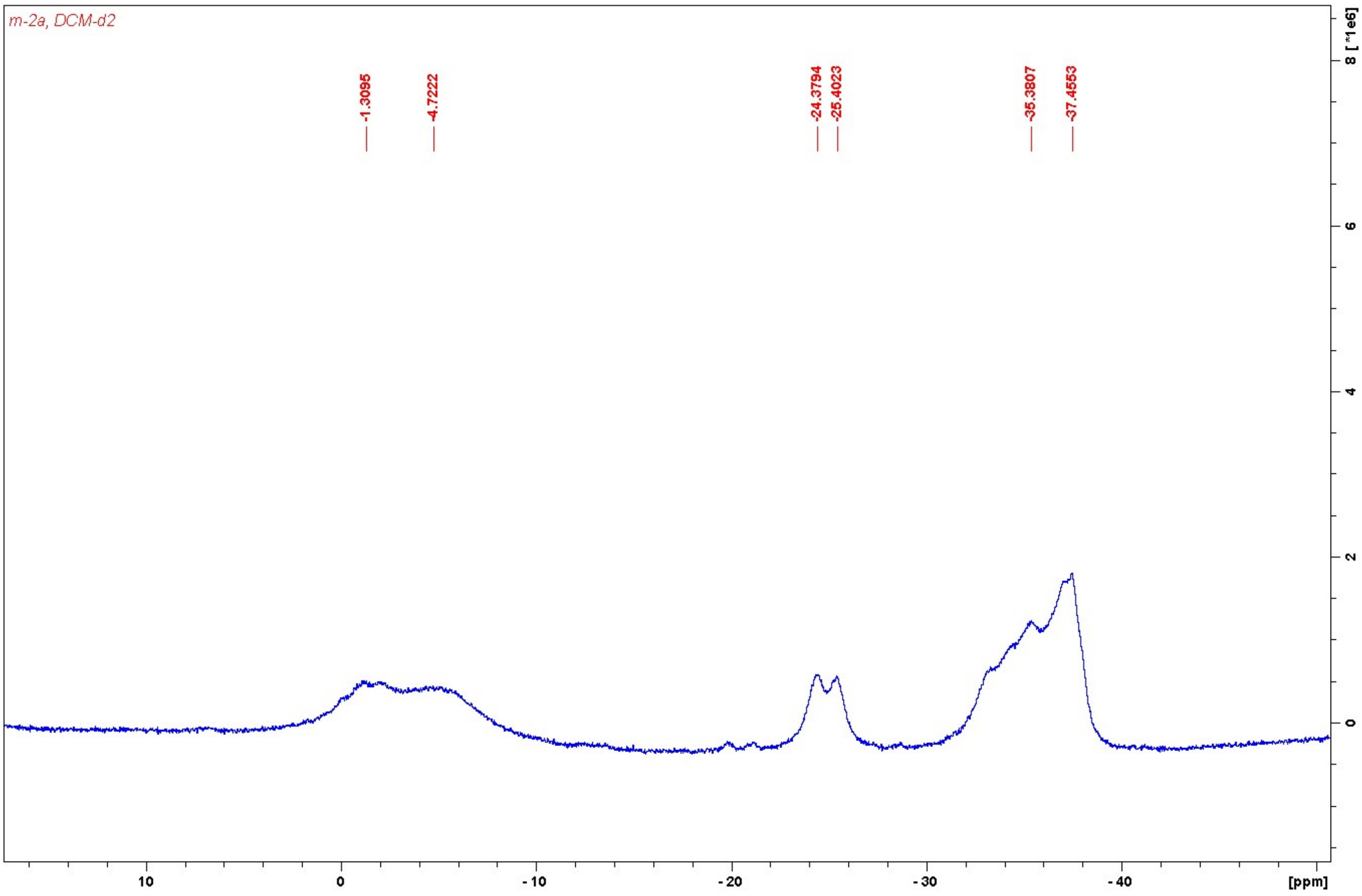

Supplementary Figure 27. ^{11}**B NMR spectrum of *m*-2a.**

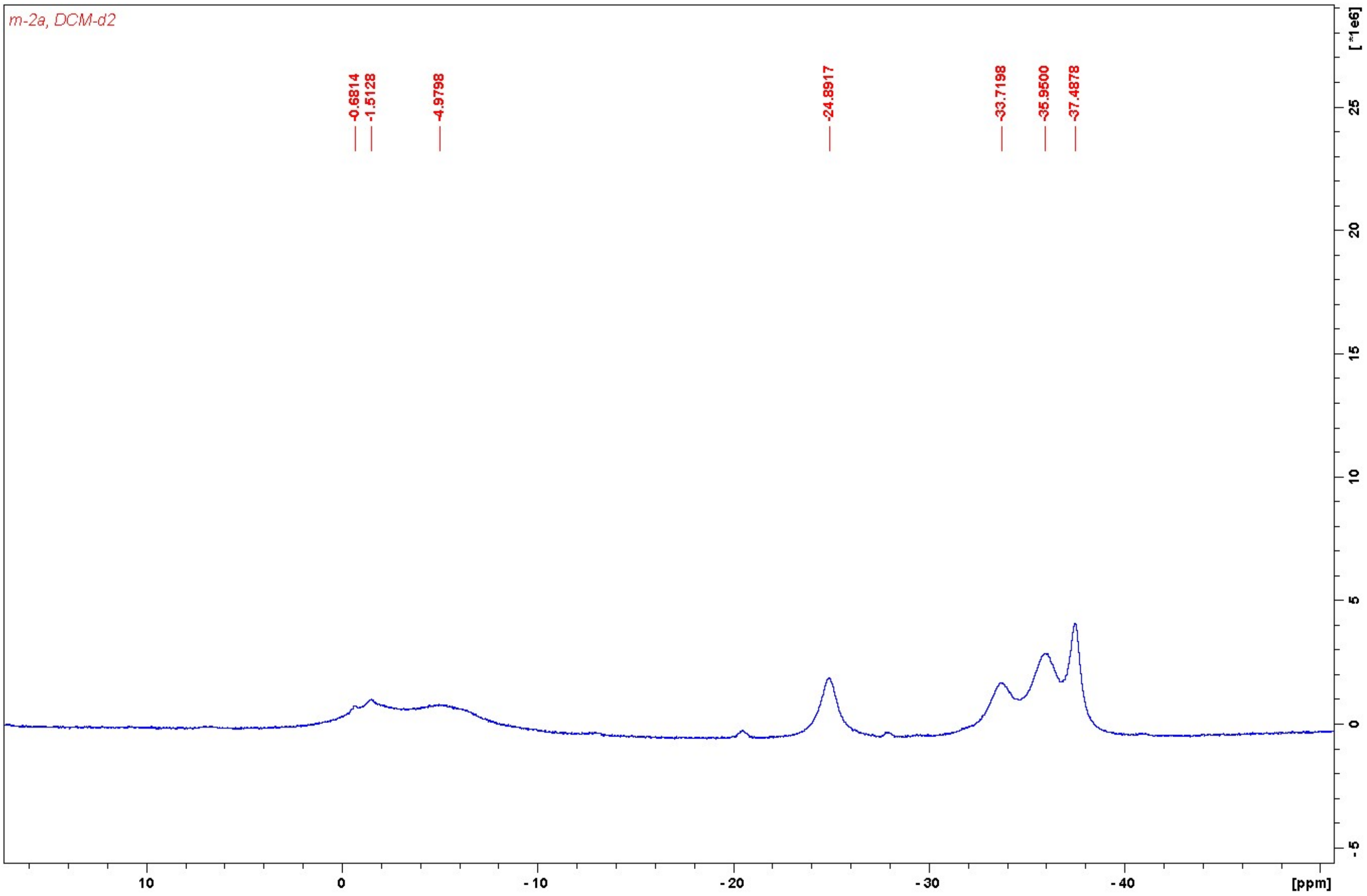

Supplementary Figure 28. ^{11}**B{**^{1}**H} NMR spectrum of *m*-2a.**

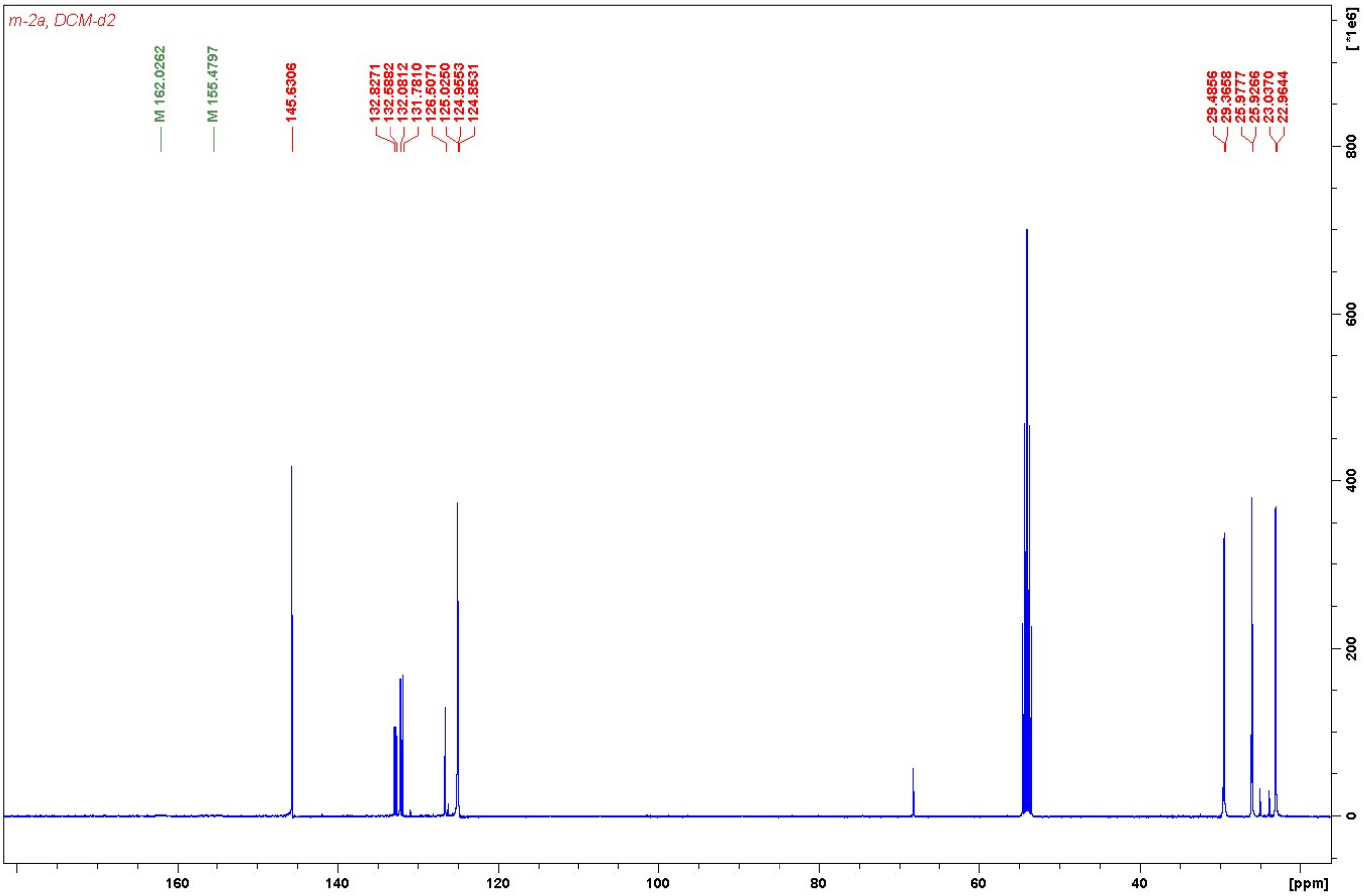

Supplementary Figure 29. ^{13}**C{**^{1}**H} NMR spectrum of *m*-2a.**

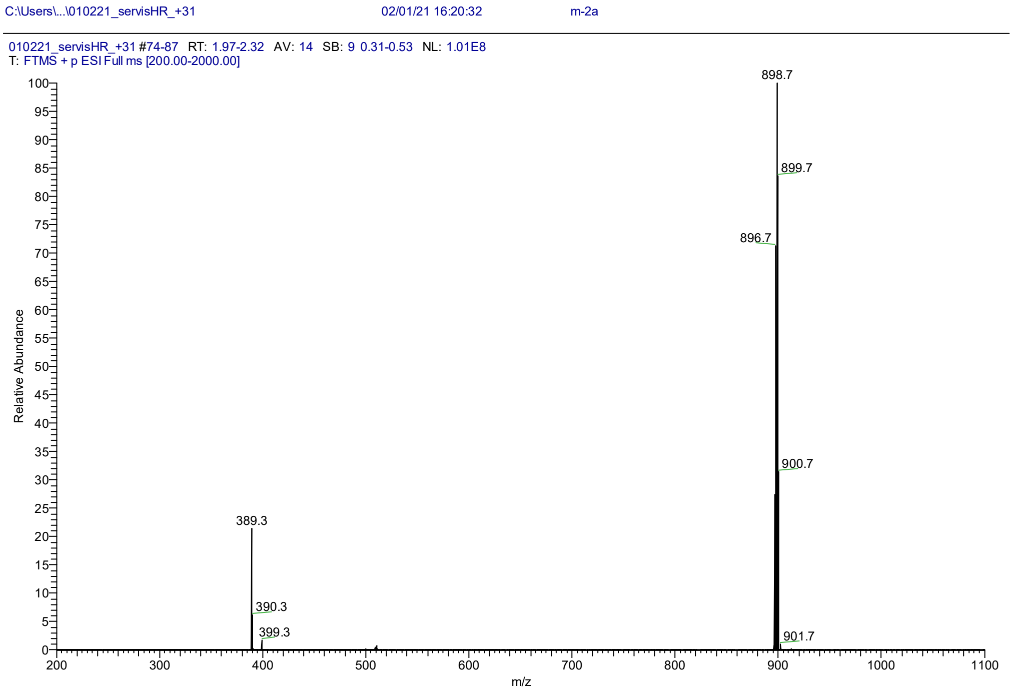

Supplementary Figure 30. MS spectrum (ESI+) of *m*-2a: [:IPr + H]$^+$, *m/z* 389.3; [M]$^+$, *m/z* 899.7. HRMS (ESI+): *m/z* calculated for $C_{56}H_{83}N_4B_8^+$ 899.73567, found 899.73586 (0.21 ppm).

Supplementary Table 5. Crystal data and structure refinement for *m*-2a.

Chemical formula	$C_{57.50}H_{86.75}B_8Cl_5N_4$
M_r	1097.78
Crystal system, space group	Orthorhombic, *Pna*2$_1$
Temperature (K)	150
a, *b*, *c* (Å)	21.8120 (9), 10.4169 (4), 55.569 (3)
V (Å^3)	12626.1 (9)
Z	8
Radiation type	Mo *K*α
μ (mm^{-1})	0.27
Crystal size (mm)	0.59 × 0.54 × 0.35
Data collection	
Diffractometer	Bruker D8 - Venture
Absorption correction	Multi-scan<br>*SADABS2016*/2 - Bruker AXS area detector scaling and absorption correction
T_{min}, T_{max}	0.851, 0.914
No. of measured, independent and observed [$I > 2σ(I)$] reflections	63853, 23406, 17033
R_{int}	0.040
(sin θ/λ)$_{max}$ (Å^{-1})	0.611
Refinement	
$R[F^2 > 2σ(F^2)]$, $wR(F^2)$, S	0.060, 0.159, 1.04
No. of reflections	23406
No. of parameters	1461
No. of restraints	202
H-atom treatment	H atoms treated by a mixture of independent and constrained refinement
$Δρ_{max}$, $Δρ_{min}$ (e Å^{-3})	0.38, −0.62
Absolute structure	Flack x determined using 2778 quotients [(I+)-(I-)]/[(I+)+(I-)] (Parsons, Flack and Wagner, Acta Cryst. B69 (2013) 249-259). Refined as a 2-component twin.
Absolute structure parameter	0.475 (15) – this value is meaningless as the crystal is weak anomalous scatterer

Synthesis of [2-NHC^{Dip}-1,10-$C_2B_8H_9$]$^+$[HCl_2]$^-$ (*p*-2a)

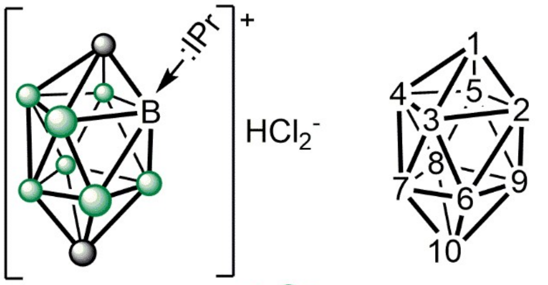

***p*-2a**

Solution of hydrogen chloride (0.224 mL, 3M solution in CPME, 0.67 mmol) was added to a stirred solution of ***p*-2** (201 mg, 0.22 mmol) in toluene (5 mL) at -70 °C. The resulting suspension was warmed to room temperature and filtrated. The crude product was recrystallized in the mixture DCM/Hexane. Yield 23 mg, 18 %. **Dp** 182 °C. **Anal. Calc**. for $C_{29}H_{46}B_8Cl_2N_2$ (580.08): C 60.0, H 8.0, N 4.8; found C 60.0, H 8.1, N 4.9**.** **^{1}H NMR** (25 °C, dcm-d$_2$, 500 MHz): δ = 1.18 (d, $^3J(^1H-^1H)$ = 6.7 Hz, 12H, CH(C*H*$_3$)$_2$), 1.36 (d, $^3J(^1H-^1H)$ = 6.8 Hz, 12H, CH(C*H*$_3$)$_2$), 2.23 (sept, $^3J(^1H-^1H)$ = 6.7 Hz, 4H, C*H*(CH$_3$)$_2$), 5.94 (s broad, 1H, *H*Cl$_2$), 6.37 (s broad, 1H, BC*H*), 6.99 (s broad, 1H, BC*H*), 7.41 (d, $^3J(^1H-^1H)$ = 7.8 Hz, 4H, *m*-Ar*H*), 7.67 (t, $^3J(^1H-^1H)$ = 7.8 Hz, 2H, *p*-Ar*H*), 8.27 (s, 2H, C*H*=C*H*) ppm. **^{11}B NMR** (25 °C, dcm-d$_2$, 160.42 MHz): δ = -13.6 (d, $^1J(^1H-^{11}B)$ = 149 Hz, 4B), -11.6 (d, $^1J(^1H-^{11}B)$ = 165 Hz, 2B), -11.1 (d, $^1J(^1H-^{11}B)$ = 163 Hz, 2B) ppm. **^{13}C{^{1}H} NMR** (25 °C, dcm-d$_2$, 125.76 Hz): δ = 22.0, 26.5 (s, CH(*C*H$_3$)$_2$), 29.8 (s, *C*H(CH$_3$)$_2$), 99.6, 104.0 (s broad, B*C*H), 125.4 (s, *m*-Ar*C*), 130.0 (s, *C*H=*C*H), 131.7 (s, *ipso*-Ar*C*), 133.2 (s, *p*-Ar*C*), 145.6 (s, *o*-Ar*C*) ppm, signal of N*C*N not observed.

Spectroscopic characterization of [2-NHC^{Dip}-1,10-$C_2B_8H_9$]$^+$[HCl_2]$^-$ (*p*-2a)

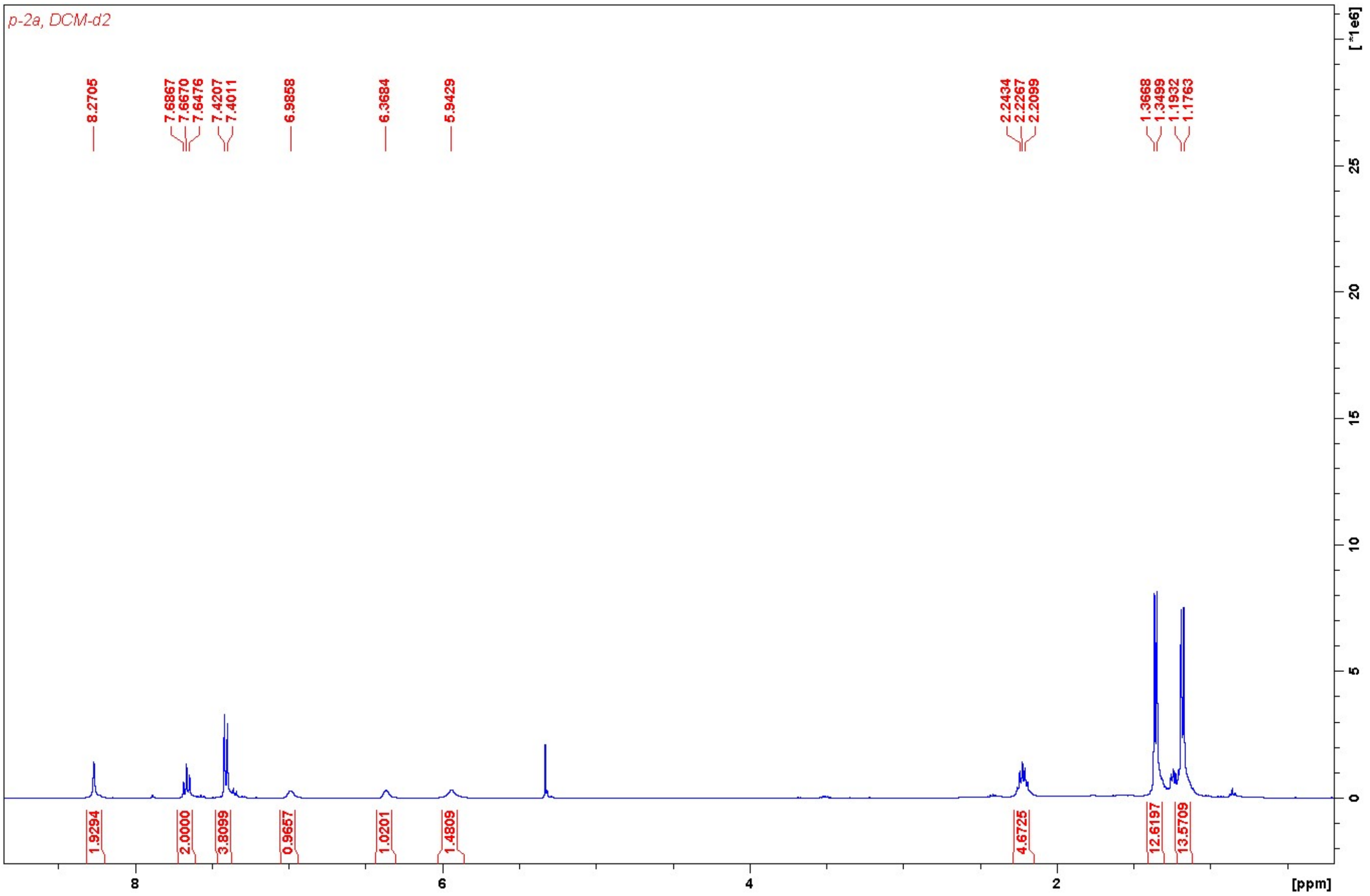

Supplementary Figure 31. ^{1}H NMR spectrum of *p*-2a.

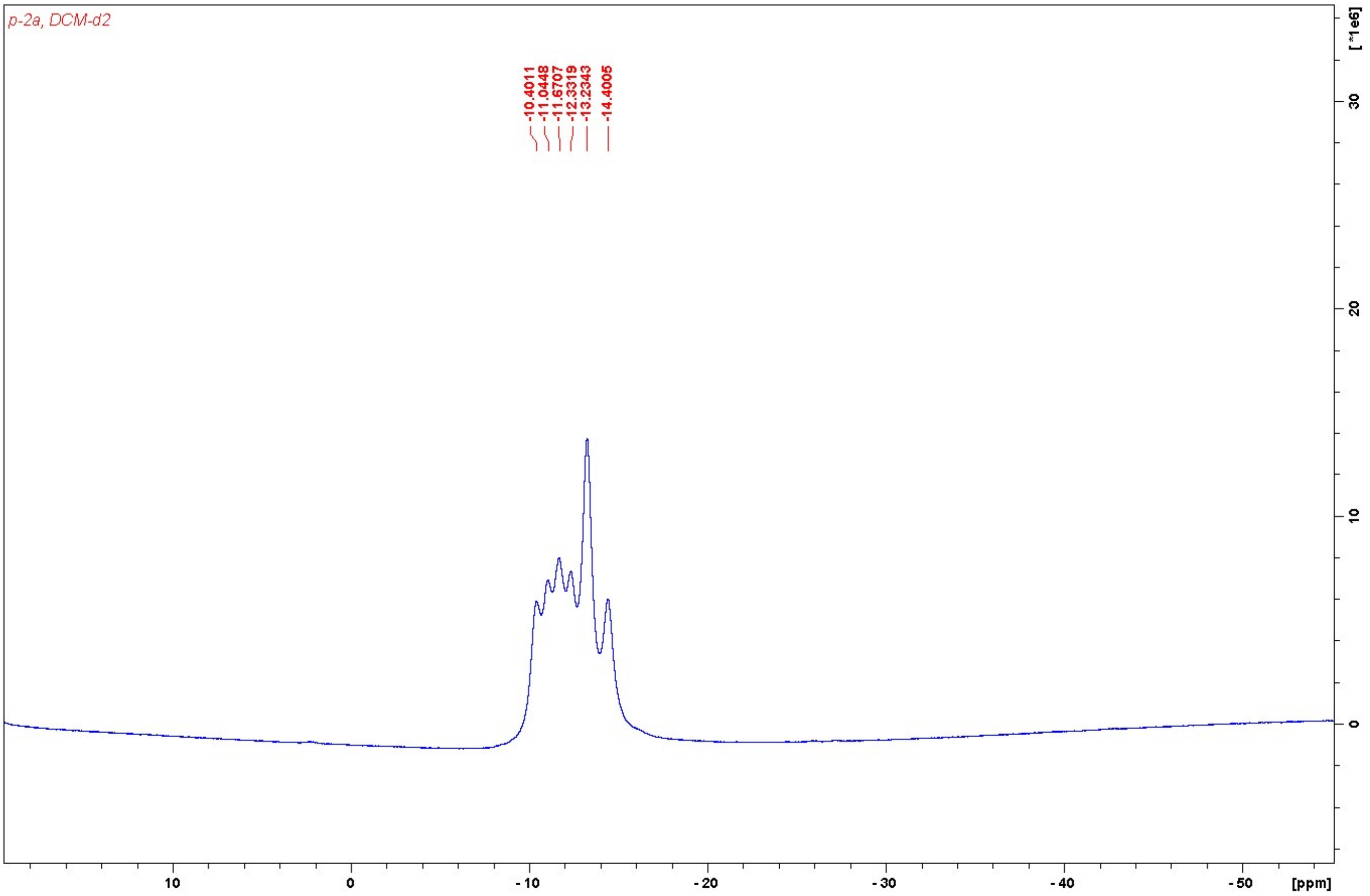

Supplementary Figure 32. ^{11}**B NMR spectrum of *p*-2a.**

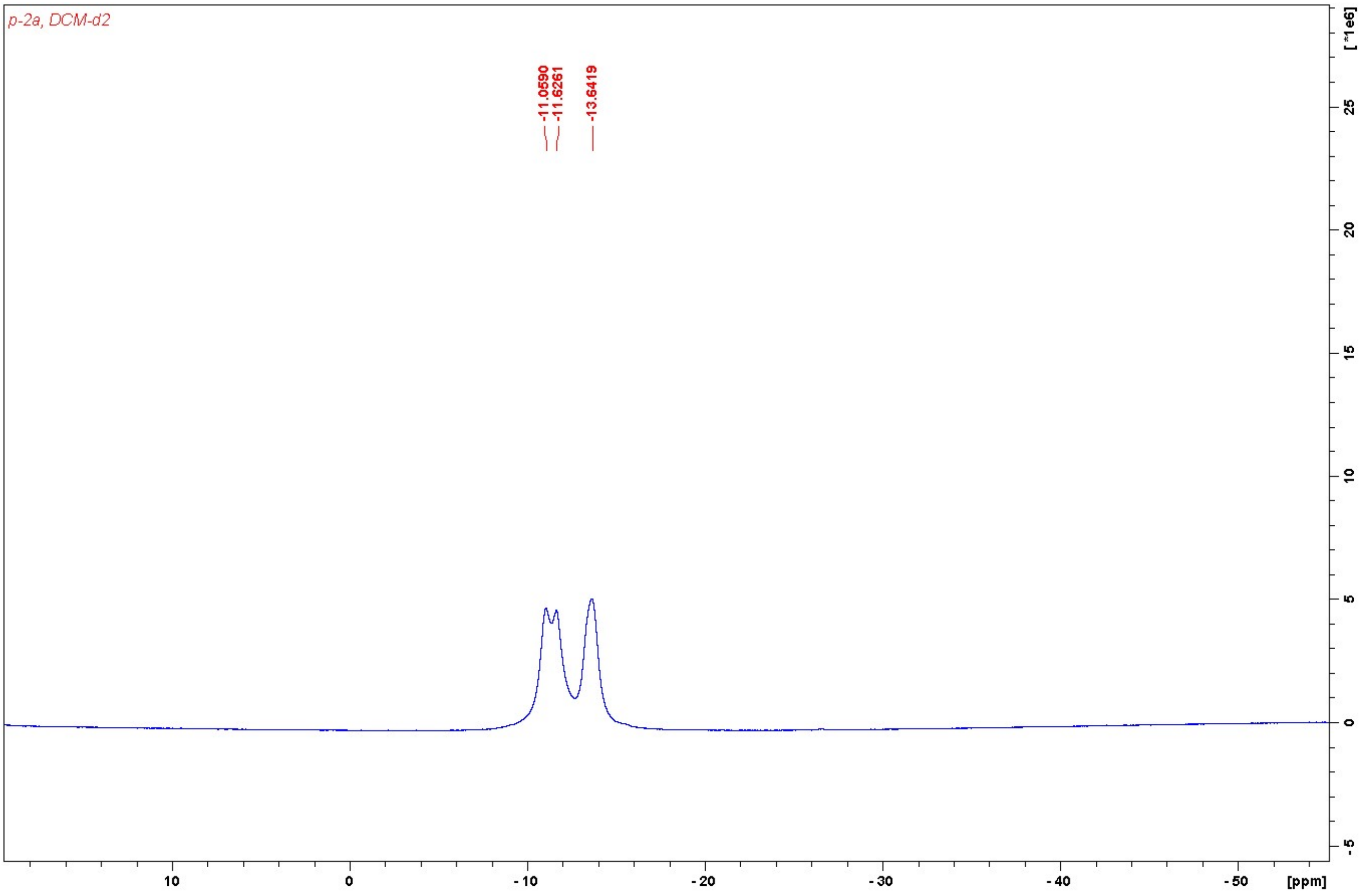

Supplementary Figure 33. ^{11}**B{**^{1}**H} NMR spectrum of *p*-2a.**

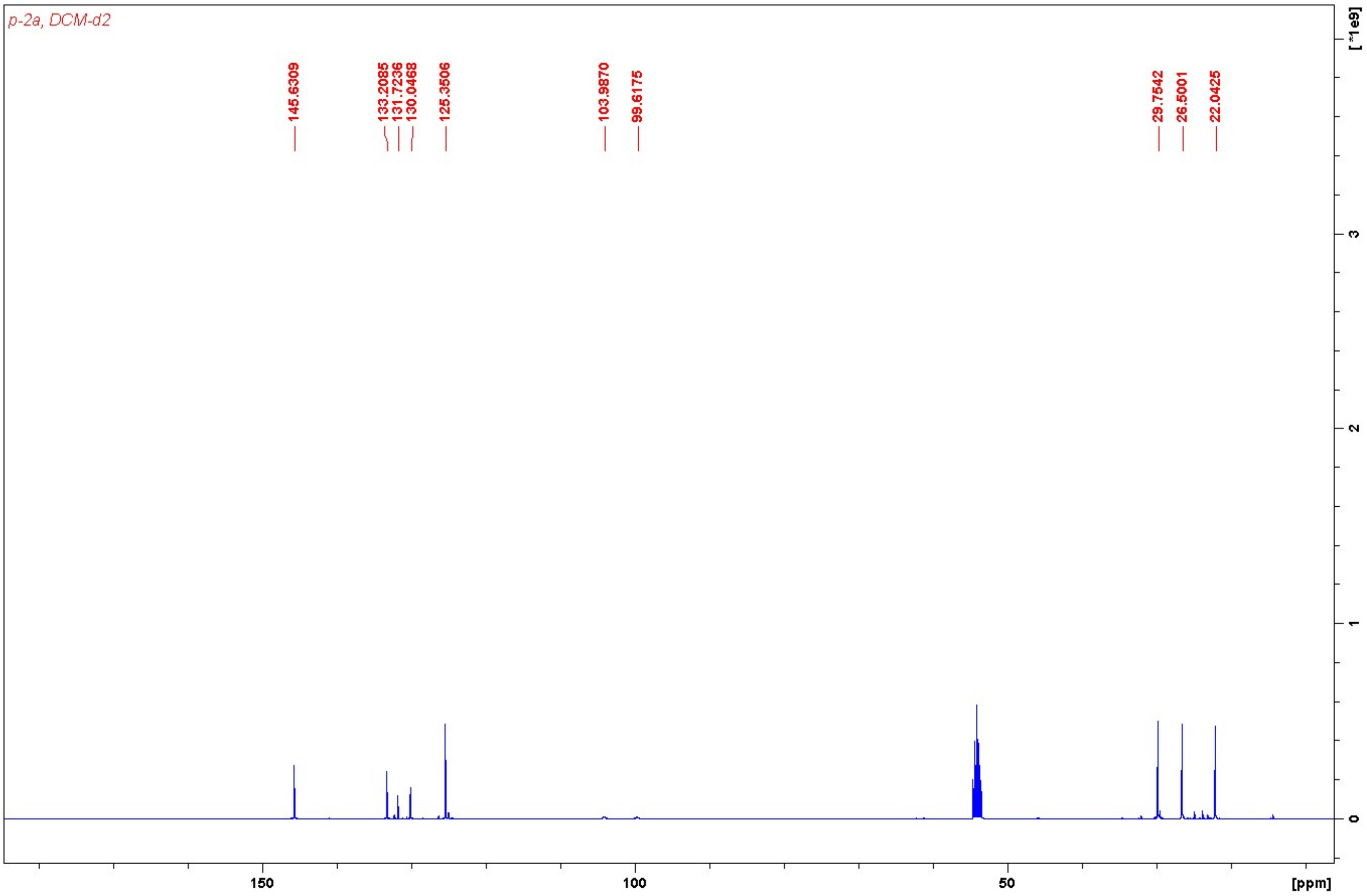

Supplementary Figure 34. $^{13}C\{^{1}H\}$ **NMR spectrum of *p*-2a.**

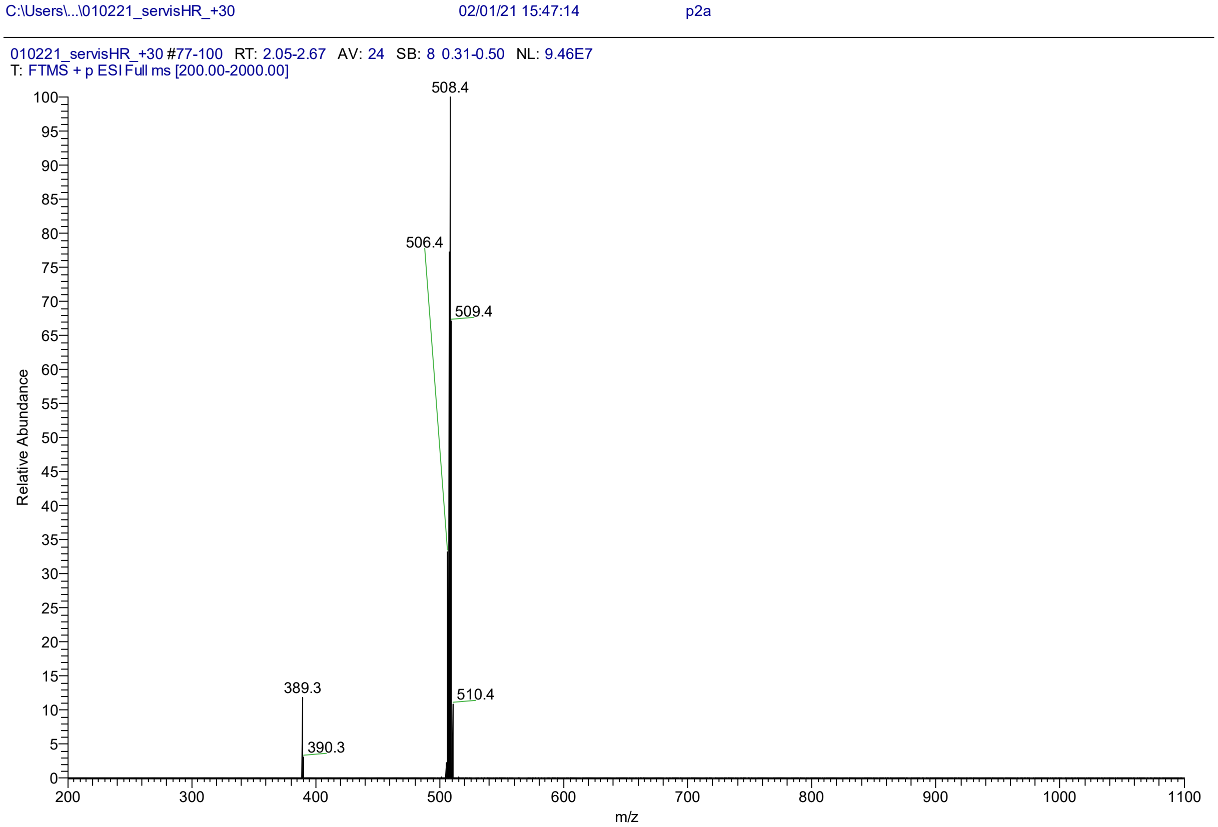

Supplementary Figure 35. MS spectrum (ESI+) of *p*-2a: [:IPr + H]$^+$, *m/z* 389.3; [M]$^+$, *m/z* 509.4. HRMS (ESI+): *m/z* calculated for $C_{29}H_{45}N_2B_8^+$ 509.43272, found 509.43254 (-0.35 ppm).

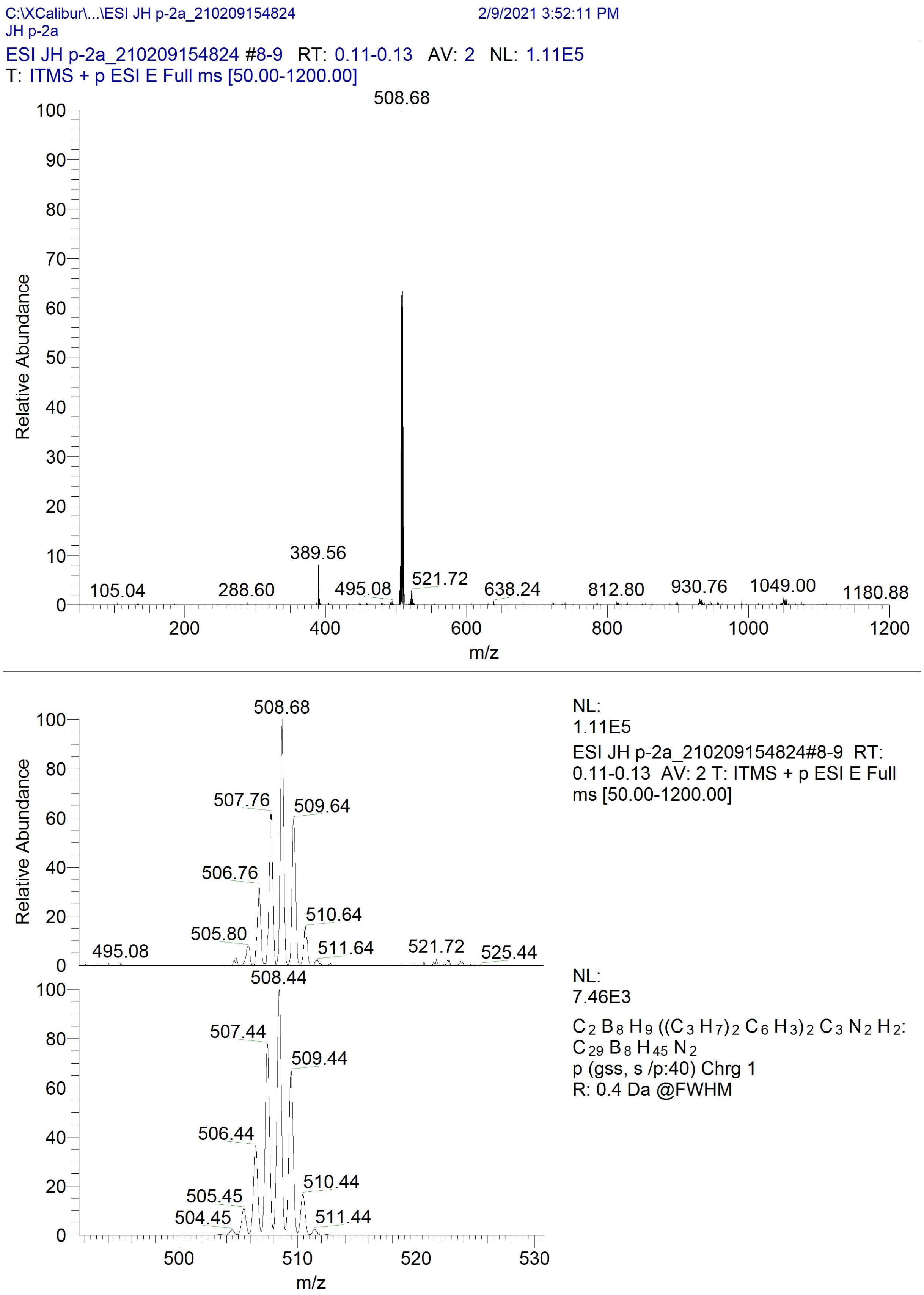

Supplementary Figure 36. MS spectrum (ESI+) of *p*-2a measured from the solution of D_2O: $[M]^+$, *m/z* 509.64.

Supplementary Table 6. Crystal data and structure refinement for *p*-2a.

Chemical formula	$C_{29}H_{46}B_8Cl_2N_2$
M_r	580.06
Crystal system, space group	Monoclinic, $P2_1/m$
Temperature (K)	150
a, *b*, *c* (Å)	9.3925 (13), 17.5494 (18), 10.739 (2)
β (°)	111.368 (8)
V (Å^3)	1648.4 (4)
Z	2
Radiation type	Mo *K*α
μ (mm^{-1})	0.22
Crystal size (mm)	0.59 × 0.55 × 0.37
Data collection	
Diffractometer	Bruker D8 - Venture
Absorption correction	Multi-scan<br>*SADABS2016*/2 - Bruker AXS area detector scaling and absorption correction
T_{min}, T_{max}	0.678, 0.746
No. of measured, independent and observed [$I > 2\sigma(I)$] reflections	40137, 3900, 3141
R_{int}	0.058
$(\sin \theta/\lambda)_{max}$ (Å^{-1})	0.650
Refinement	
$R[F^2 > 2\sigma(F^2)]$, $wR(F^2)$, S	0.039, 0.101, 1.04
No. of reflections	3900
No. of parameters	224
No. of restraints	180
H-atom treatment	H atoms treated by a mixture of independent and constrained refinement
$\Delta\rho_{max}$, $\Delta\rho_{min}$ (e Å^{-3})	0.28, −0.32

Synthesis of 4-NHC^{Dip}-2,7,8-$PC_2B_8H_9$ (*o*-1a)

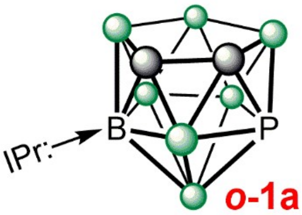

o-1a

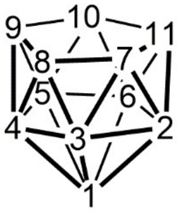

Phosphorus trichloride (0.023 mL, 0.27 mmol) was added to a stirred solution of ***o*-1** (136 mg, 0.27 mmol) and triethylamine (0.149 mL, 1.07 mmol) in tetrahydrofuran (5 mL) at -70 °C. The resulting suspension was warmed to room temperature and the volatiles were removed in vacuo. The solid residue was extracted by benzene (5 mL). The solvent was removed under reduced pressure to give ***o*-1a** in the form of colourless powder. Yield 88 mg, 80 %. **Dp** 223 °C. **Anal. Calc**. for $C_{29}H_{45}B_8N_2P$ (539.14): C 64.6, H 8.4, N 5.2; found C 64.6, H 8.3, N 5.3. **^{1}H NMR** (25 °C, C_6D_6, 500 MHz): δ = 0.96 (d, $^3J(^1H-^1H)$ = 6.9 Hz, 6H, CH(C*H*$_3$)$_2$), 0.98 (d, $^3J(^1H-^1H)$ = 6.9 Hz, 6H, CH(C*H*$_3$)$_2$), 1.35 (d, $^3J(^1H-^1H)$ = 6.8 Hz, 6H, CH(C*H*$_3$)$_2$), 1.42 (d, $^3J(^1H-^1H)$ = 6.8 Hz, 6H, CH(C*H*$_3$)$_2$), 1.54 (s broad, 1H, BC*H*), 1.71 (s broad, 1H, BC*H*), 2.84 (sept, $^3J(^1H-^1H)$ = 6.7 Hz, 2H, C*H*(CH$_3$)$_2$), 3.02 (sept, $^3J(^1H-^1H)$ = 6.7 Hz, 2H, C*H*(CH$_3$)$_2$), 6.53 (s, 2H, C*H*=C*H*), 7.05 (m, 4H, *m*-Ar*H*), 7.52 (t, $^3J(^1H-^1H)$ = 7.7 Hz, 2H, *p*-Ar*H*) ppm. **^{11}B NMR** (25 °C, C_6D_6, 160.42 MHz): δ = -39.6 (s broad, 1B, B1), -29.5 (s broad, 1B, B3), -24.9 (s broad, 1B, B11), -17.3 (s broad, 1B, B4), -10.4 – (-5.9) (m broad, 4B, B5,6,9,10) ppm. **^{13}C{^{1}H} NMR** (25 °C, C_6D_6, 125.76 Hz): δ = 22.99, 23.04 (s, CH(*C*H$_3$)$_2$), 25.1 (s broad, B*C*H), 26.2, 26.5 (s, CH(*C*H$_3$)$_2$), 28.1 (s broad, B*C*H), 29.7, 29.8 (s, *C*H(CH$_3$)$_2$), 124.7, 125.1 (s, *m*-Ar*C*), 125.8 (s, *C*H=*C*H), 132.0 (s, *p*-Ar*C*), 134.5 (s, *ipso*-Ar*C*), 146.2, 146.5 (s, *o*-Ar*C*) ppm, signal for N*C*N not observed. **^{31}P NMR** (25 °C, C_6D_6, 202.46 MHz): δ = -82.5 (s broad) ppm.

Spectroscopic characterization of 7-NHC^{Dip}-11,5,6-$PC_2B_8H_9$ (*o*-1a)

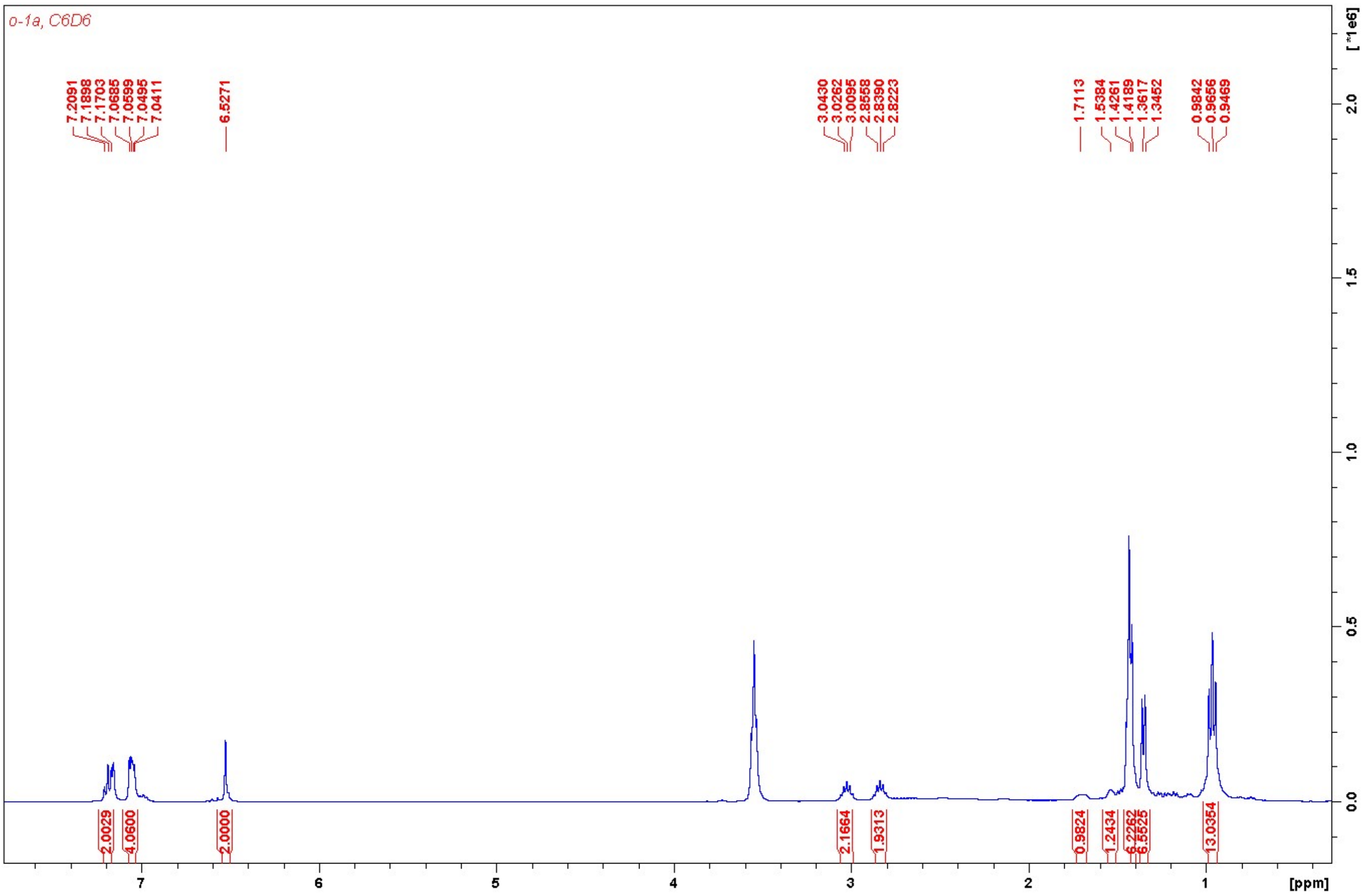

Supplementary Figure 37. ^{1}H NMR spectrum of *o*-1a.

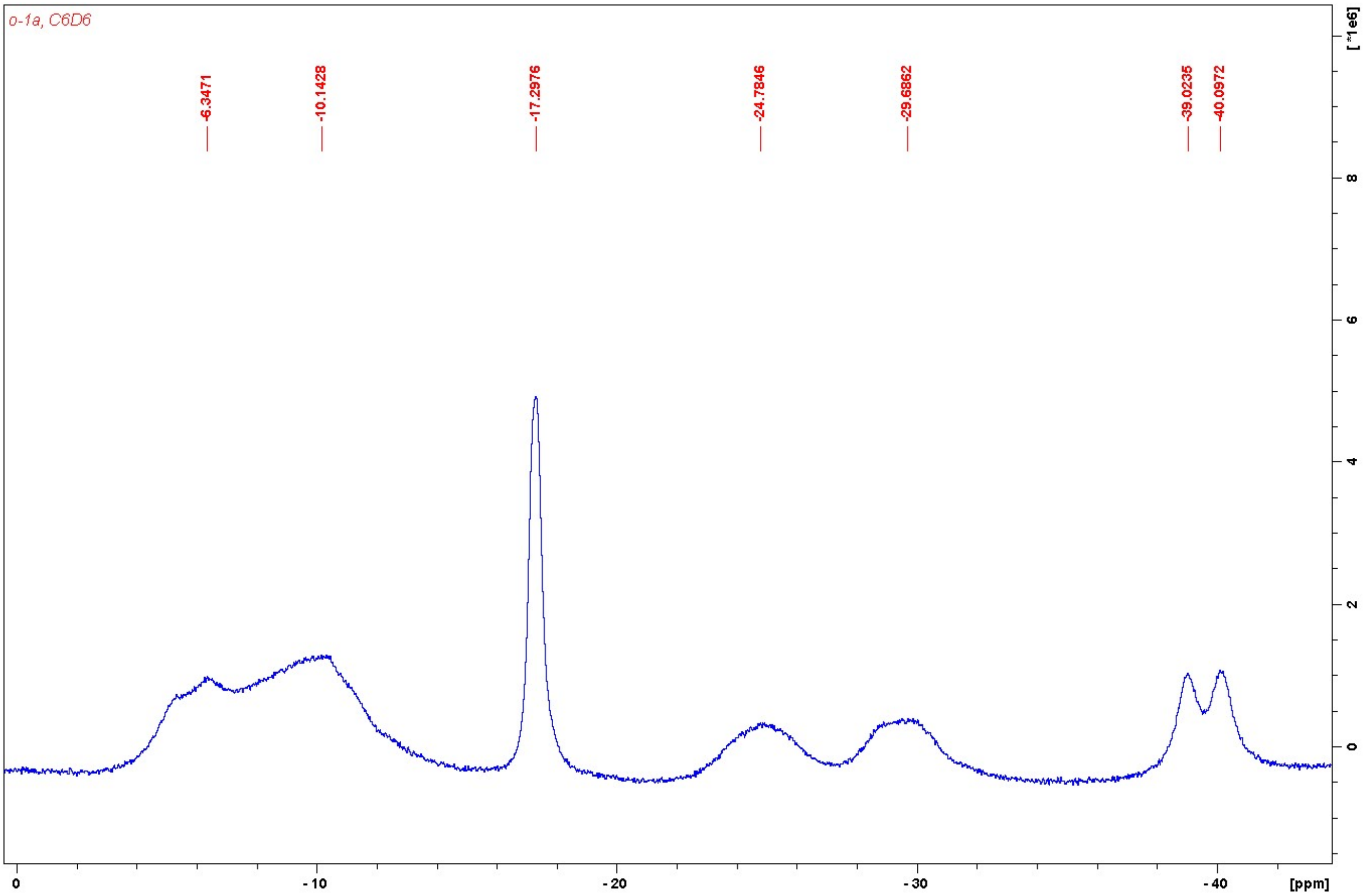

Supplementary Figure 38. ^{11}B NMR spectrum of *o*-1a.

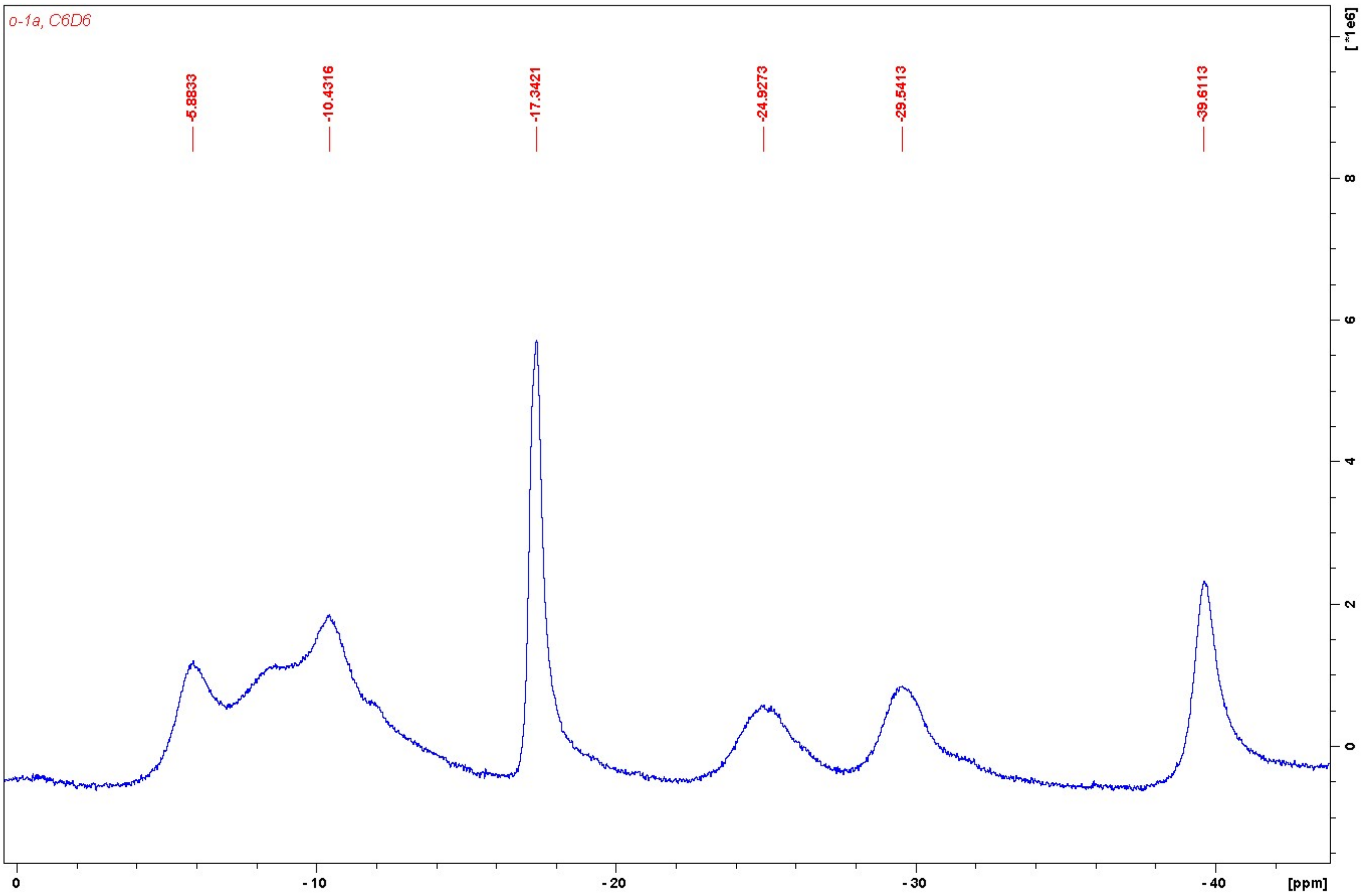

Supplementary Figure 39. $^{11}B\{^{1}H\}$ **NMR spectrum of *o*-1a.**

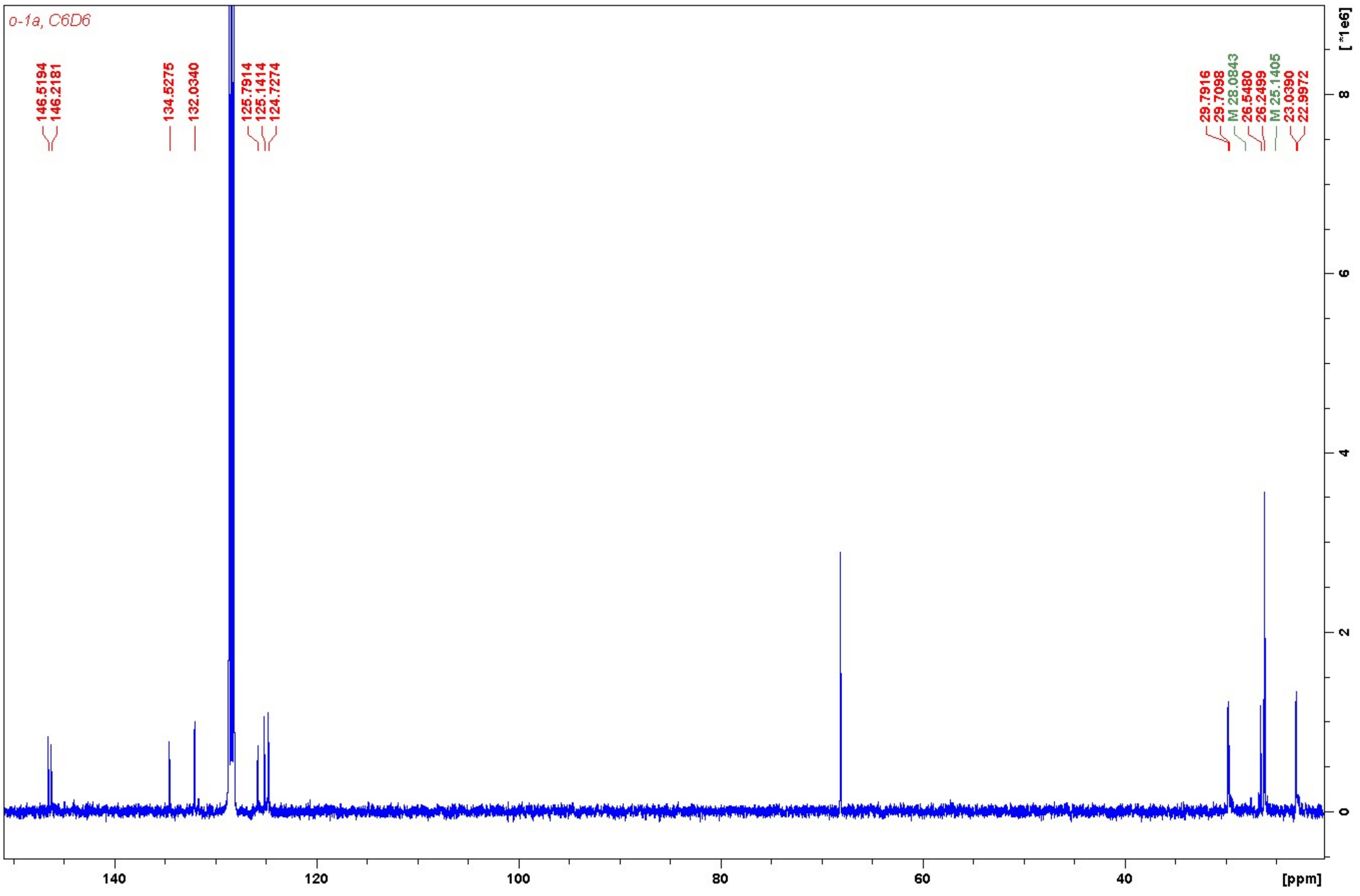

Supplementary Figure 40. $^{13}C\{^{1}H\}$ **NMR spectrum of *o*-1a.**

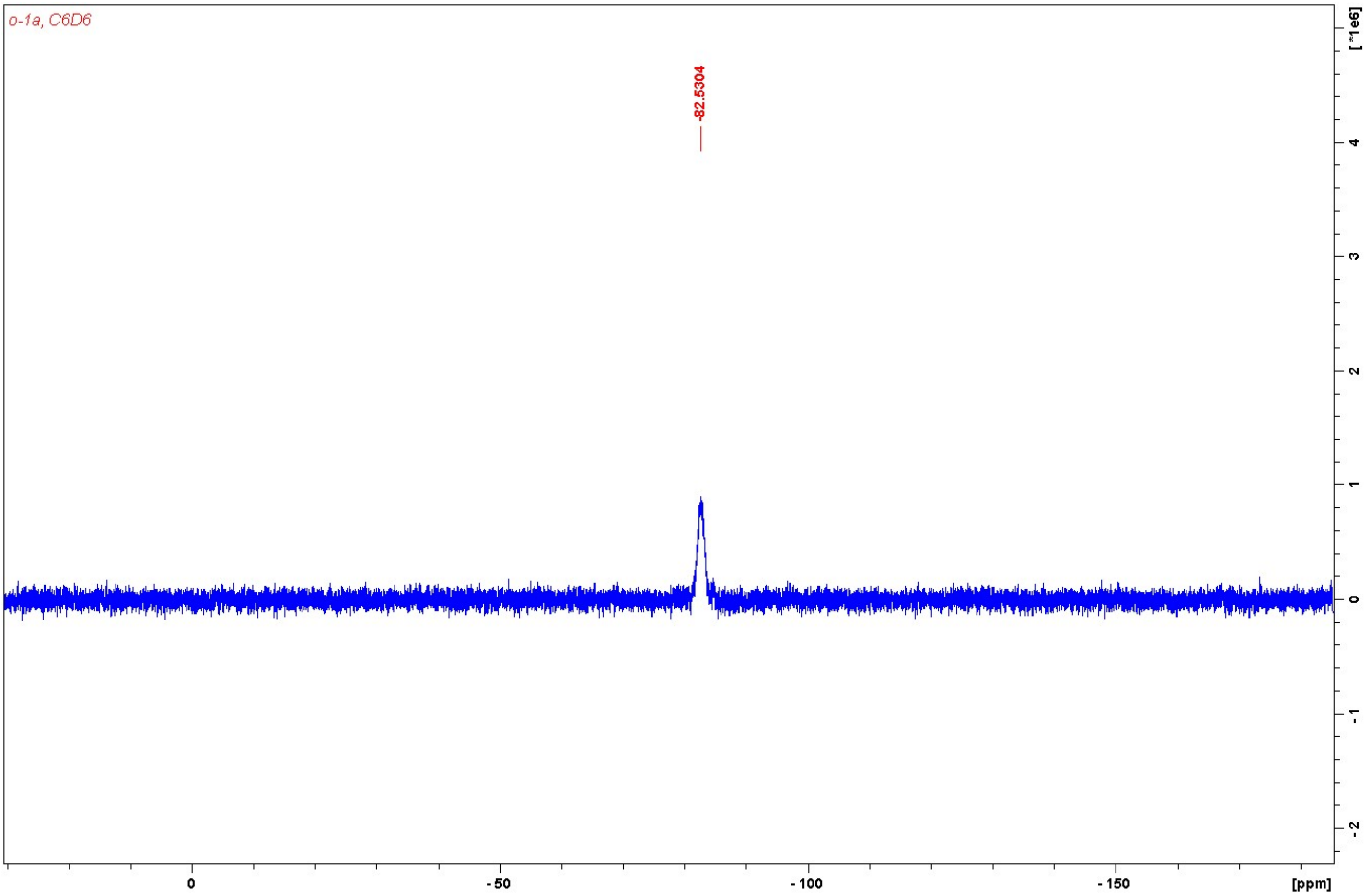

Supplementary Figure 41. ^{31}P NMR spectrum of *o*-1a.

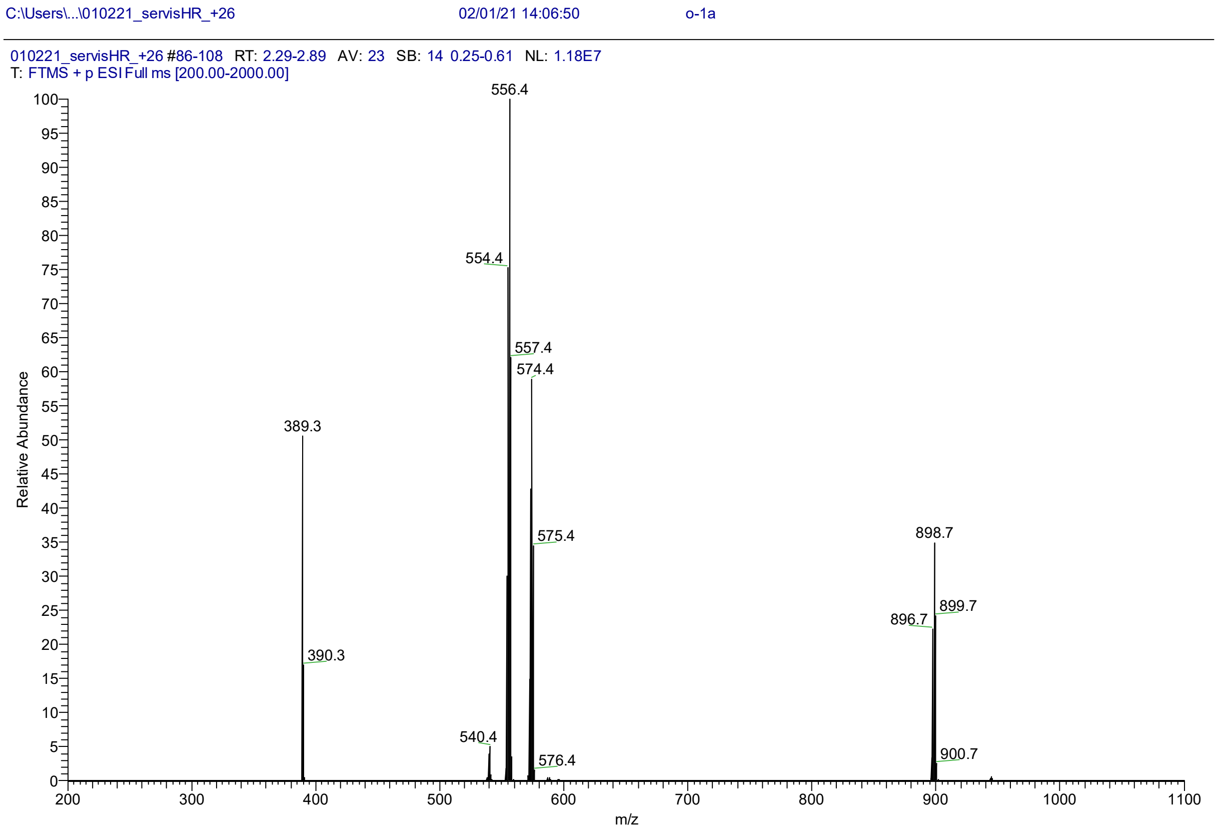

Supplementary Figure 42. MS spectrum (ESI+) of *o*-1a (reaction mixture): [:IPr + H]$^+$, *m/z* 389.3; [M + H]$^+$, *m/z* 541.4; [M + O +H]$^+$ (phosphine oxide), *m/z* 557.4; [M + O + H_2O + H]$^+$ (hydrated phosphine oxide), *m/z* 575.4; two-ligand complex, *m/z* 899.7. HRMS (ESI+): *m/z* calculated for $C_{29}H_{46}N_2B_8P^+$ 541.41376, found 541.41422 (0.86 ppm).

Supplementary Table 7. Crystal data and structure refinement for *o*-1a.

Chemical formula	$C_{29}H_{45}B_8N_2P$
M_r	539.12
Crystal system, space group	Monoclinic, *C*2/*c*
Temperature (K)	150
a, *b*, *c* (Å)	10.116 (3), 17.475 (6), 37.562 (18)
β (°)	93.328 (18)
V (Å^3)	6629 (4)
Z	8
Radiation type	Mo *K*α
μ (mm^{-1})	0.10
Crystal size (mm)	0.56 × 0.46 × 0.33
Data collection	
Diffractometer	Bruker D8 - Venture
Absorption correction	Multi-scan<br>*SADABS2016*/2 - Bruker AXS area detector scaling and absorption correction
T_{min}, T_{max}	0.675, 0.971
No. of measured, independent and observed [$I > 2\sigma(I)$] reflections	20151, 5707, 3858
R_{int}	0.104
$(\sin \theta/\lambda)_{max}$ (Å^{-1})	0.595
Refinement	
$R[F^2 > 2\sigma(F^2)]$, $wR(F^2)$, S	0.174, 0.481, 0.94
No. of reflections	5707
No. of parameters	427
No. of restraints	547
H-atom treatment	H atoms treated by a mixture of independent and constrained refinement
	$w = 1/[\sigma^2(F_o^2) + (0.2909P)^2 + 131.620P]$<br>where $P = (F_o^2 + 2F_c^2)/3$
$\Delta\rho_{max}$, $\Delta\rho_{min}$ (e Å^{-3})	0.61, −1.10

Synthesis of 10-NHC^{Dip}-7-(=O)-7,8,9-$PC_2B_8H_9$ (*o*-1b)

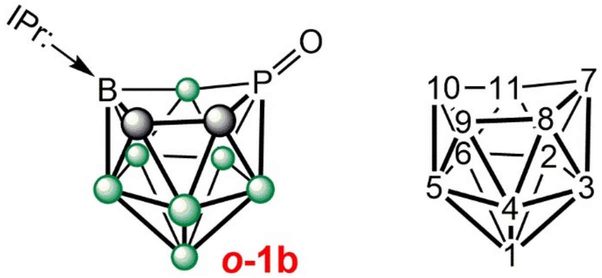

Schlenk flask was charged by the solution of compund ***o*-1a** () in benzene and than closed by a rubber septum stabbed by needle. The flask was left to stand overnight. The colourles precipitate of ***o*-1b** was filtrated and dried in vacuo. Yield 88 mg, 74 %. **Dp** 170 °C. **Anal. Calc.** for $C_{29}H_{45}B_8N_2OP$ (555.14): C 62.7, H 8.2, N 5.1; found C 62.8, H 8.3, N 5.0. **^{1}H NMR** (25 °C, C_6D_6, 500 MHz): δ = 0.79 (s broad, 1H, BC*H*), 1.13 (d, $^3J(^1H-^1H)$ = 6.8 Hz, 6H, CH(C*H*$_3$)$_2$), 1.19 (d, $^3J(^1H-^1H)$ = 6.8 Hz, 6H, CH(C*H*$_3$)$_2$), 1.31 (d, $^3J(^1H-^1H)$ = 6.7 Hz, 6H, CH(C*H*$_3$)$_2$), 1.45 (d, $^3J(^1H-^1H)$ = 6.8 Hz, 6H, CH(C*H*$_3$)$_2$), 2.32 (s broad, 1H, BC*H*), 2.53 (sept, $^3J(^1H-^1H)$ = 6.8 Hz, 2H, C*H*(CH$_3$)$_2$), 2.78 (sept, $^3J(^1H-^1H)$ = 6.8 Hz, 2H, C*H*(CH$_3$)$_2$), 7.21 (s, 2H, C*H*=C*H*), 7.33 (d, $^3J(^1H-^1H)$ = 7.8 Hz, 2H, *m*-Ar*H*), 7.37 (d, $^3J(^1H-^1H)$ = 7.8 Hz, 2H, *m*-Ar*H*), 7.57 (t, $^3J(^1H-^1H)$ = 7.7 Hz, 2H, *p*-Ar*H*) ppm. **^{11}B NMR** (25 °C, C_6D_6, 160.42 MHz): δ = -45.9 (s broad, 1B, B1), -32.1 (s broad, 1B, B5), -24.3 (s broad, 1B, B10), -20.1 (s broad, 1B, B11), -17.4 (s broad, 2B, B2,4), -14.4 (s broad, 1B, B6), -13.5 (s broad, 1B, B3) ppm. **^{13}C{^{1}H} NMR** (25 °C, C_6D_6, 125.76 Hz): δ = 22.55, 22.63, 26.0, 26.2 (s, CH(*C*H$_3$)$_2$), 29.4, 29.7 (s, *C*H(CH$_3$)$_2$), 31.7, 32.0 (s broad, B*C*H), 124.86 (s, *C*H=*C*H), 124.90, 125.3 (s, *m*-Ar*C*), 131.9 (s, *p*-Ar*C*), 133.7 (s, *ipso*-Ar*C*), 146.1, 146.3 (s, *o*-Ar*C*) ppm, signal for N*C*N not observed. **^{31}P NMR** (25 °C, C_6D_6, 202.46 MHz): δ = 32.0 (s broad) ppm.

Spectroscopic characterization of 7-NHC^{Dip}-11-(=O)-11,5,6-$PC_2B_8H_9$ (*o*-1b)

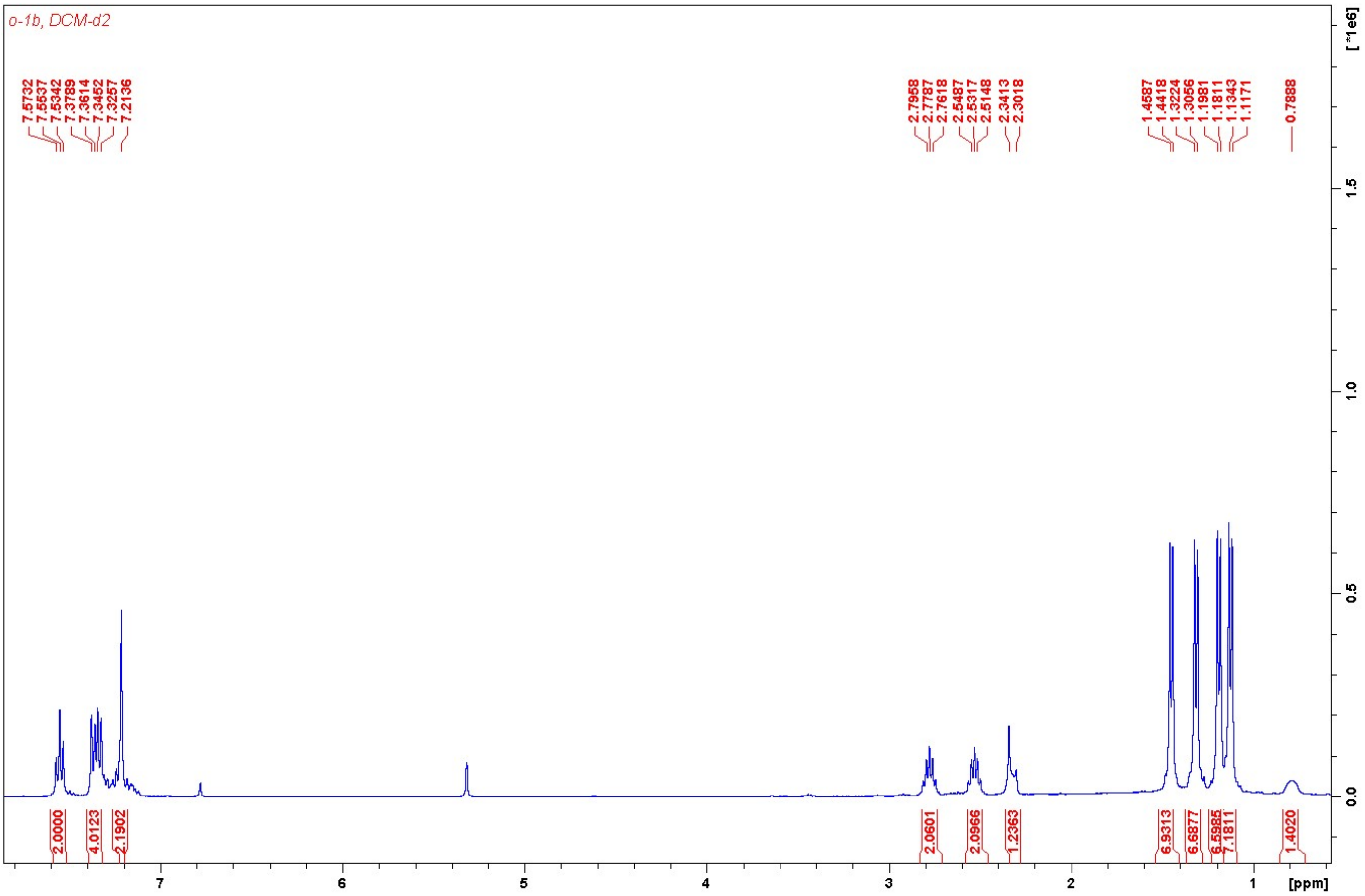

Supplementary Figure 43. ^{1}H NMR spectrum of *o*-1b.

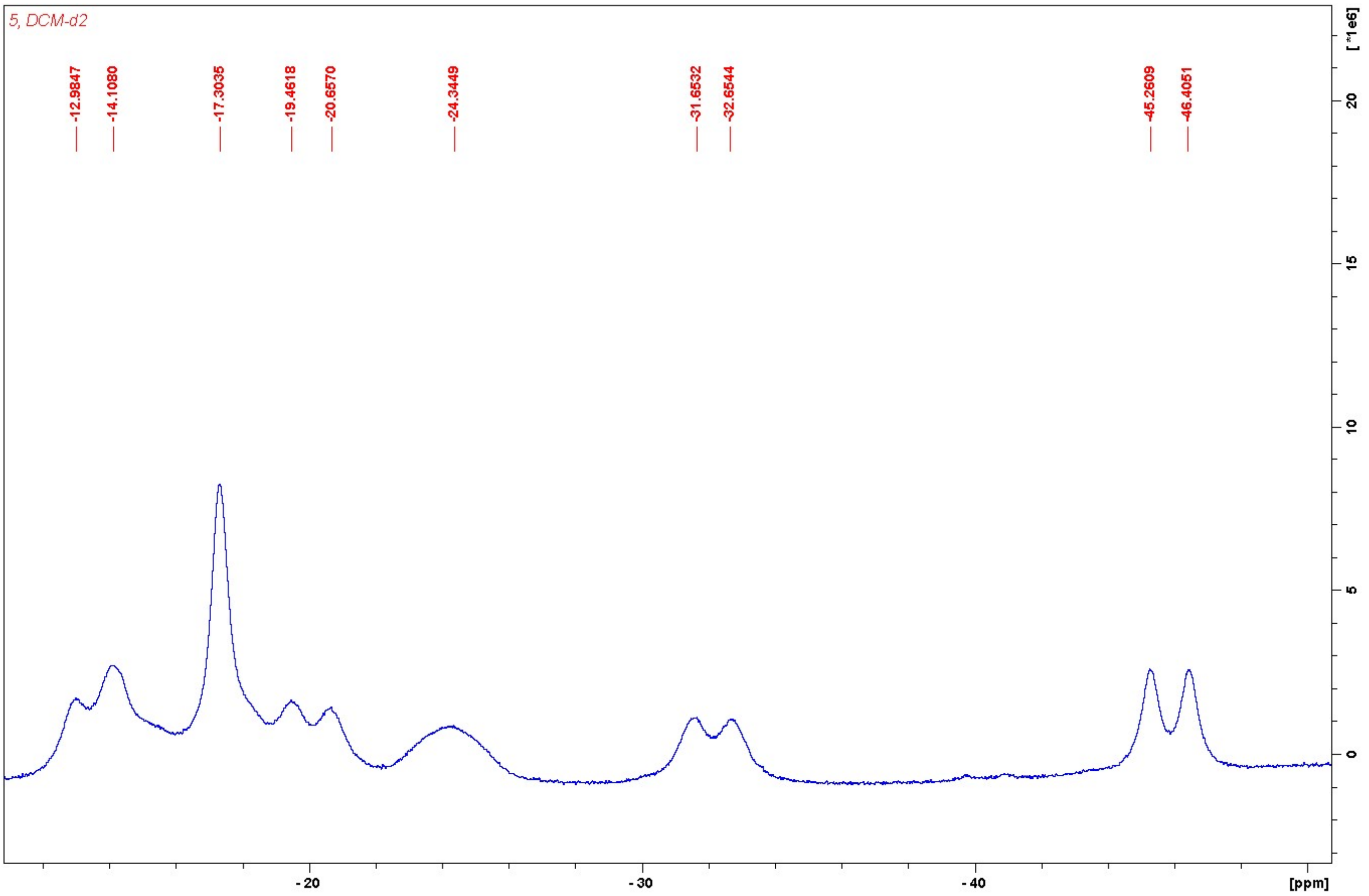

Supplementary Figure 44. ^{11}B NMR spectrum of *o*-1b.

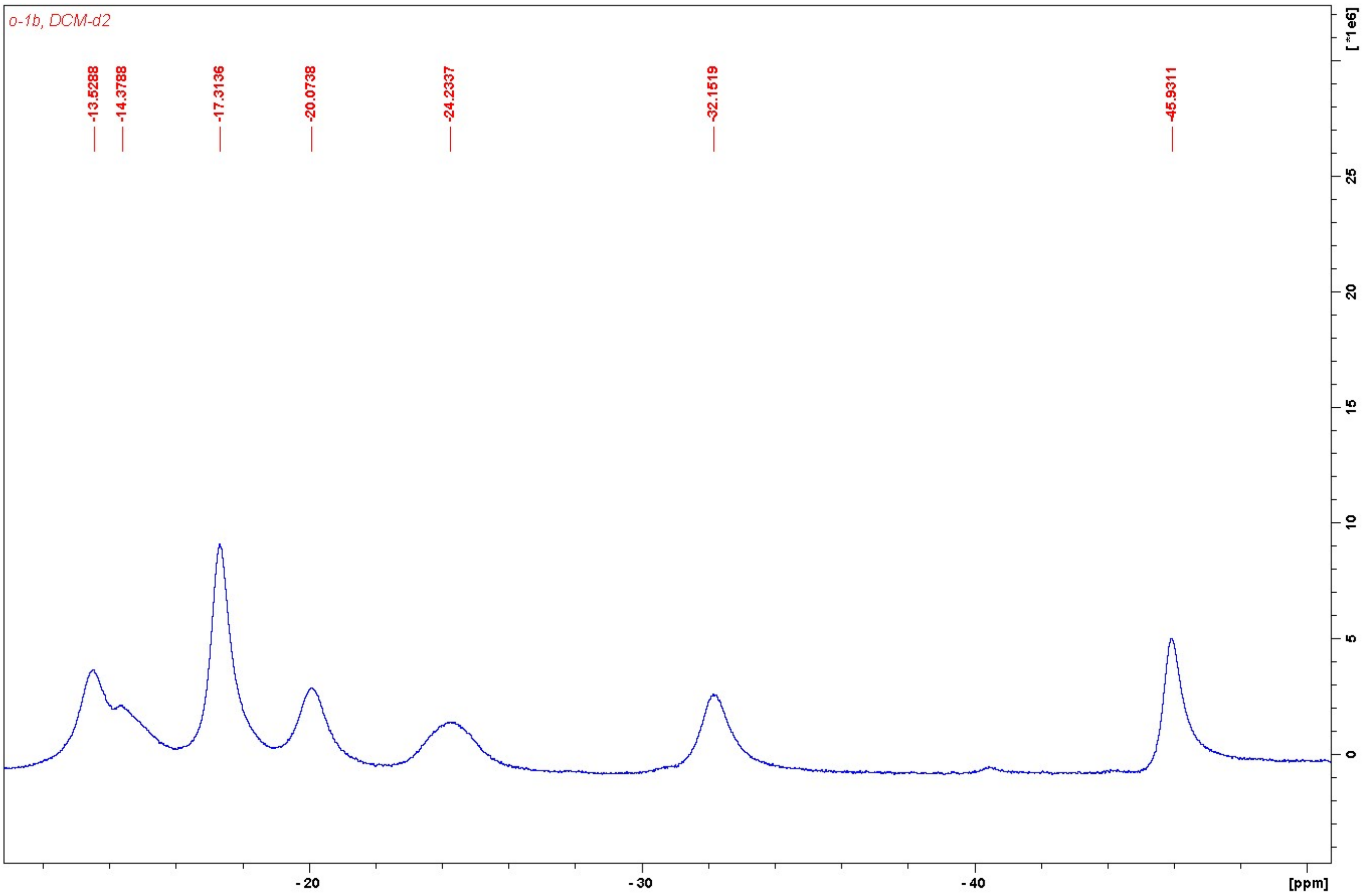

Supplementary Figure 45. $^{11}B\{^{1}H\}$ **NMR spectrum of *o*-1b.**

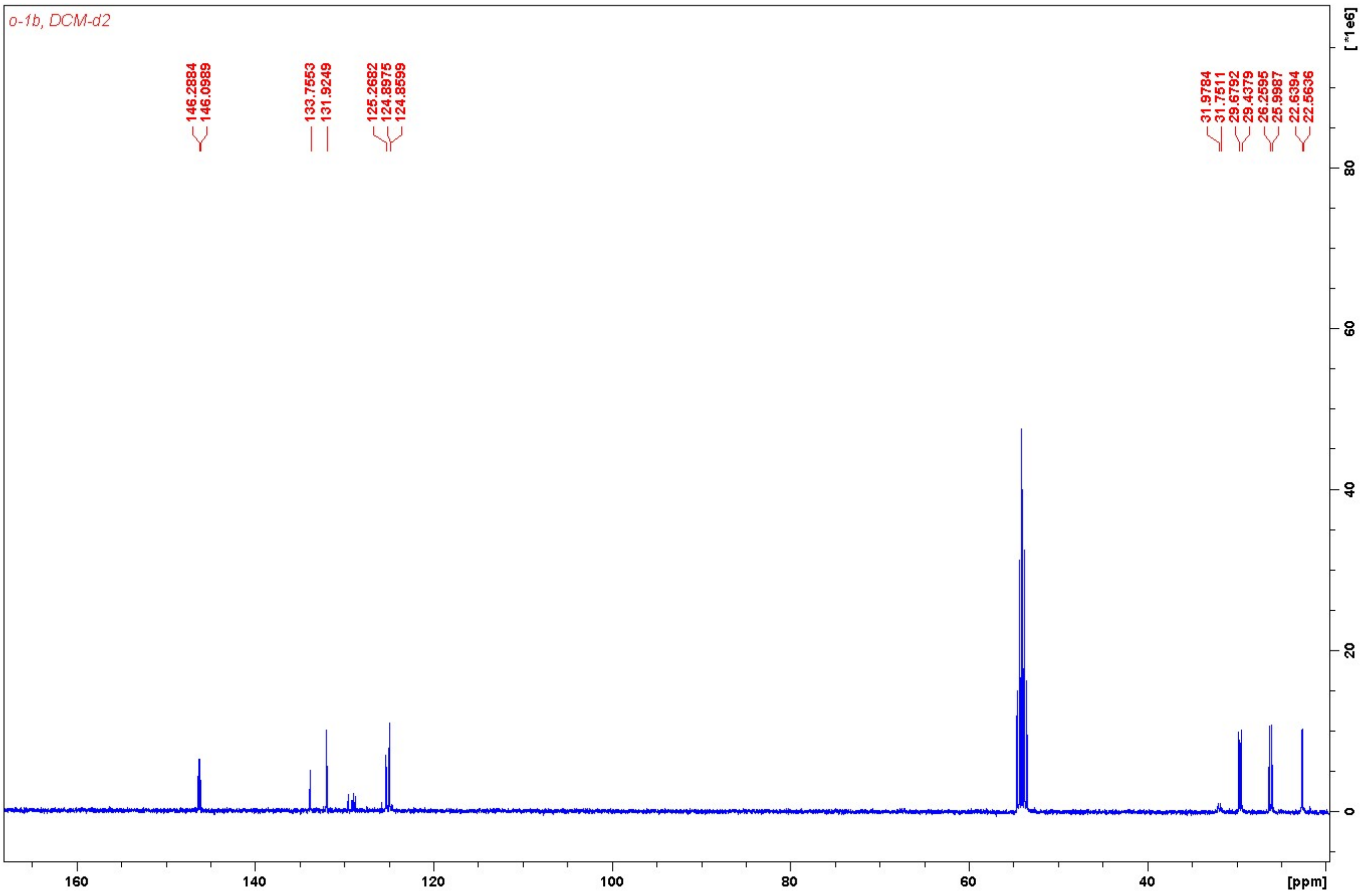

Supplementary Figure 46. $^{13}C\{^{1}H\}$ **NMR spectrum of *o*-1b.**

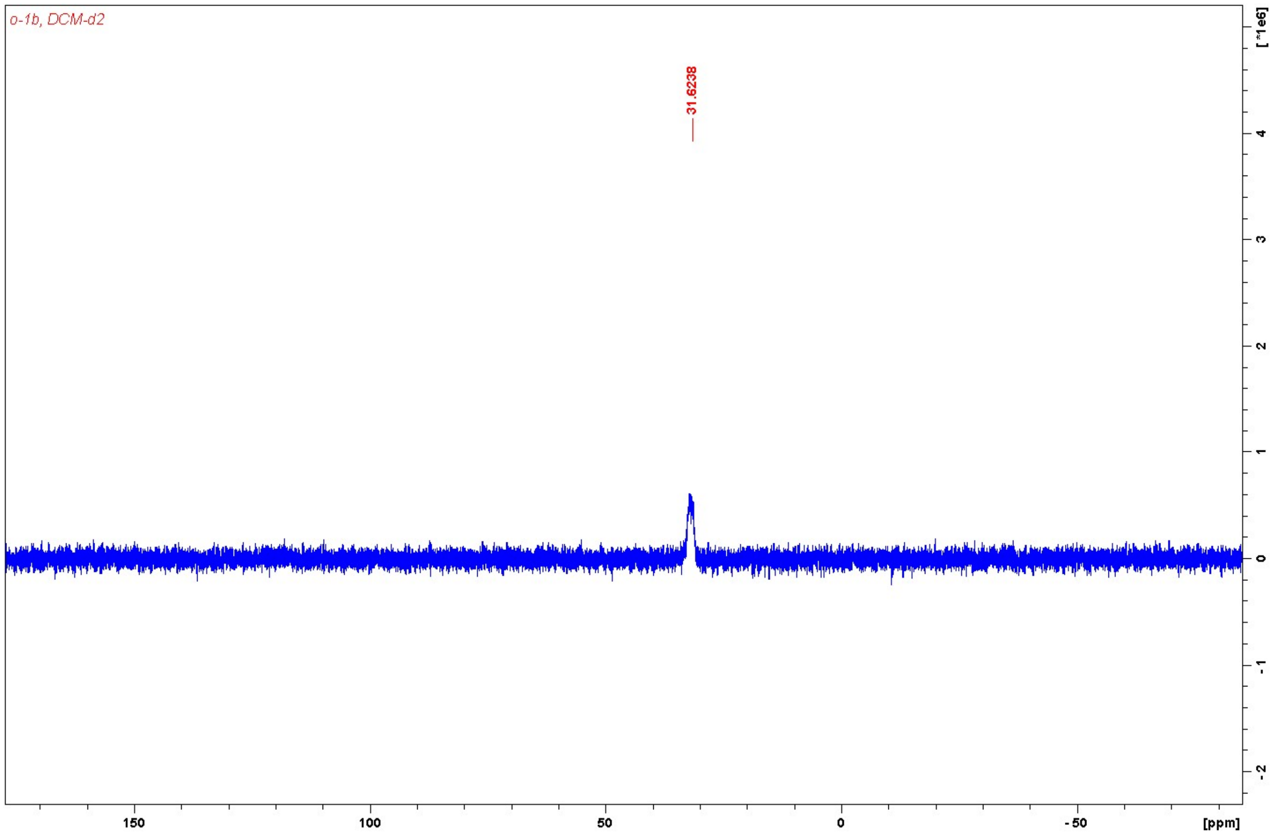

Supplementary Figure 47. ^{31}P NMR spectrum of *o*-1b.

Supplementary Table 8. Crystal data and structure refinement for *o*-1b.

Chemical formula	$C_{30}H_{47}B_8Cl_2N_2OP$
M_r	640.04
Crystal system, space group	Triclinic, *P*1
Temperature (K)	150
a, *b*, *c* (Å)	10.1870 (4), 18.0599 (8), 20.4354 (7)
α, β, γ (°)	74.523 (2), 81.375 (2), 82.558 (2)
V (Å^3)	3566.5 (2)
Z	4
Radiation type	Mo *K*α
μ (mm^{-1})	0.25
Crystal size (mm)	0.43 × 0.33 × 0.28
Data collection	
Diffractometer	Bruker D8 - Venture
Absorption correction	Multi-scan<br>*SADABS2016*/2 - Bruker AXS area detector scaling and absorption correction
T_{min}, T_{max}	0.584, 0.746
No. of measured, independent and observed [$I > 2\sigma(I)$] reflections	121539, 17811, 12047
R_{int}	0.100
$(\sin \theta/\lambda)_{max}$ (Å^{-1})	0.669
Refinement	
$R[F^2 > 2\sigma(F^2)]$, $wR(F^2)$, S	0.082, 0.219, 1.05
No. of reflections	17811
No. of parameters	881
No. of restraints	880
H-atom treatment	H atoms treated by a mixture of independent and constrained refinement
$\Delta\rho_{max}$, $\Delta\rho_{min}$ (e Å^{-3})	0.90, −1.36

Synthesis of [11,11-Cl_2-5,10-NHC^{Dip}_2-11,5,10-$PC_2B_8H_{10}$]$^+$$Cl^-$ (*o*-2b)

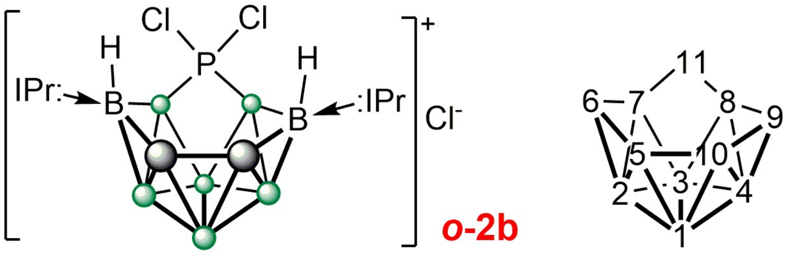

Phosphorus trichloride (0.020 mL, 0.22 mmol) was added to a stirred solution of ***o*-2b** (201 mg, 0.22 mmol) in tetrahydrofuran (5 mL) at -70 °C. The resulting suspension was warmed to room temperature and filtrated. The colourless powder of **6** was dried in vacuo. Yield 88 mg, 38 %. **Dp** 175 °C. **Anal. Calc**. for $C_{56}H_{82}B_8Cl_3N_4P$ (1035.10): C 65.0, H 8.0, N 5.4; found C 65.0, H 8.1, N 5.3. **^{1}H NMR** (25 °C, dcm-d_2, 500 MHz): δ = 1.08 (d, $^3J(^1H-^1H)$ = 6.8 Hz, 12H, CH(C*H*$_3$)$_2$), 1.11 (d, $^3J(^1H-^1H)$ = 6.8 Hz, 12H, CH(C*H*$_3$)$_2$), 1.23 (d, $^3J(^1H-^1H)$ = 6.8 Hz, 12H, CH(C*H*$_3$)$_2$), 1.29 (d, $^3J(^1H-^1H)$ = 6.8 Hz, 12H, CH(C*H*$_3$)$_2$), 1.76 (s broad, 2H, BC*H*), 2.36 (sept, $^3J(^1H-^1H)$ = 6.7 Hz, 8H, C*H*(CH$_3$)$_2$), 7.25 (s, 4H, C*H*=C*H*), 7.28 (d, $^3J(^1H-^1H)$ = 7.8 Hz, 8H, *m*-Ar*H*), 7.52 (t, $^3J(^1H-^1H)$ = 7.8 Hz, 4H, *p*-Ar*H*) ppm. **^{11}B NMR** (25 °C, dcm-d_2, 160.42 MHz): δ = -37.3 (s broad, 1B, B3), -34.3 (s broad, 2B, B6,9), -32.5 (s broad, 1B, B1), -7.8 (s broad, 2B, B7,8), -2.9 (s broad, 2B, B2,4) ppm. **^{13}C{^{1}H} NMR** (25 °C, dcm-d_2, 125.76 Hz): δ = 22.8, 22.9, 26.1, 26.3 (s, CH(*C*H$_3$)$_2$), 29.5, 29.7 (s, *C*H(CH$_3$)$_2$), 39.4 (s broad, B*C*H), 125.2 (s, *m*-Ar*C*), 125.3 (s, *C*H=*C*H), 125.8 (s, *m*-Ar*C*), 132.0 (s, *p*-Ar*C*), 133.0 (s, *ipso*-Ar*C*), 145.5, 145.8 (s, *o*-Ar*C*), 160.7 (s very broad, N*C*N) ppm. **^{31}P NMR** (25 °C, C_6D_6, 202.46 MHz): δ = 97.1 (s broad) ppm.

Spectroscopic characterization of [11,11-Cl_2-5,10-NHC^{Dip}_2-11,5,10-$PC_2B_8H_{10}$]$^+$$Cl^-$ (*o*-2b)

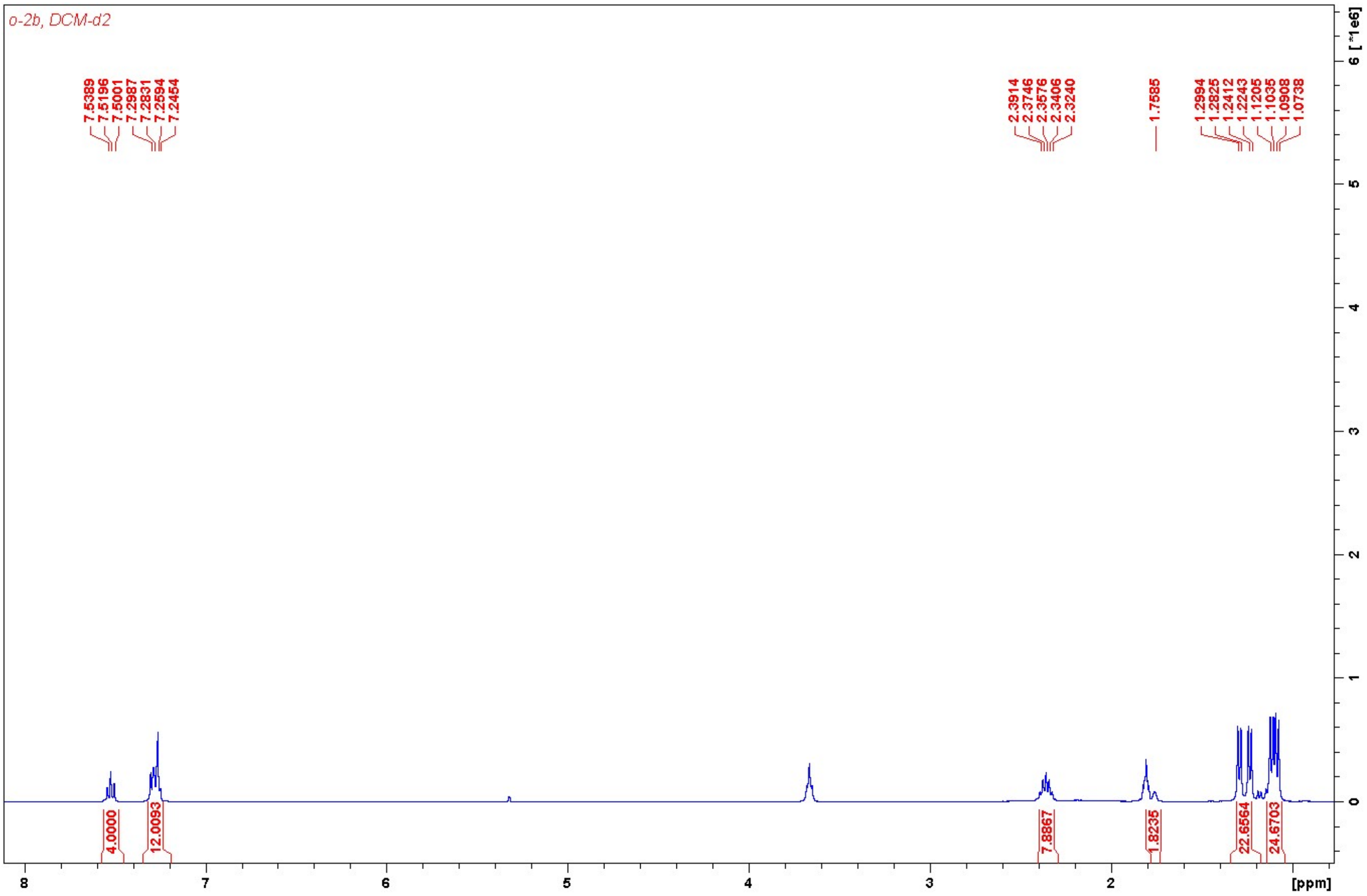

Supplementary Figure 48. ^{1}H NMR spectrum of *o*-2b.

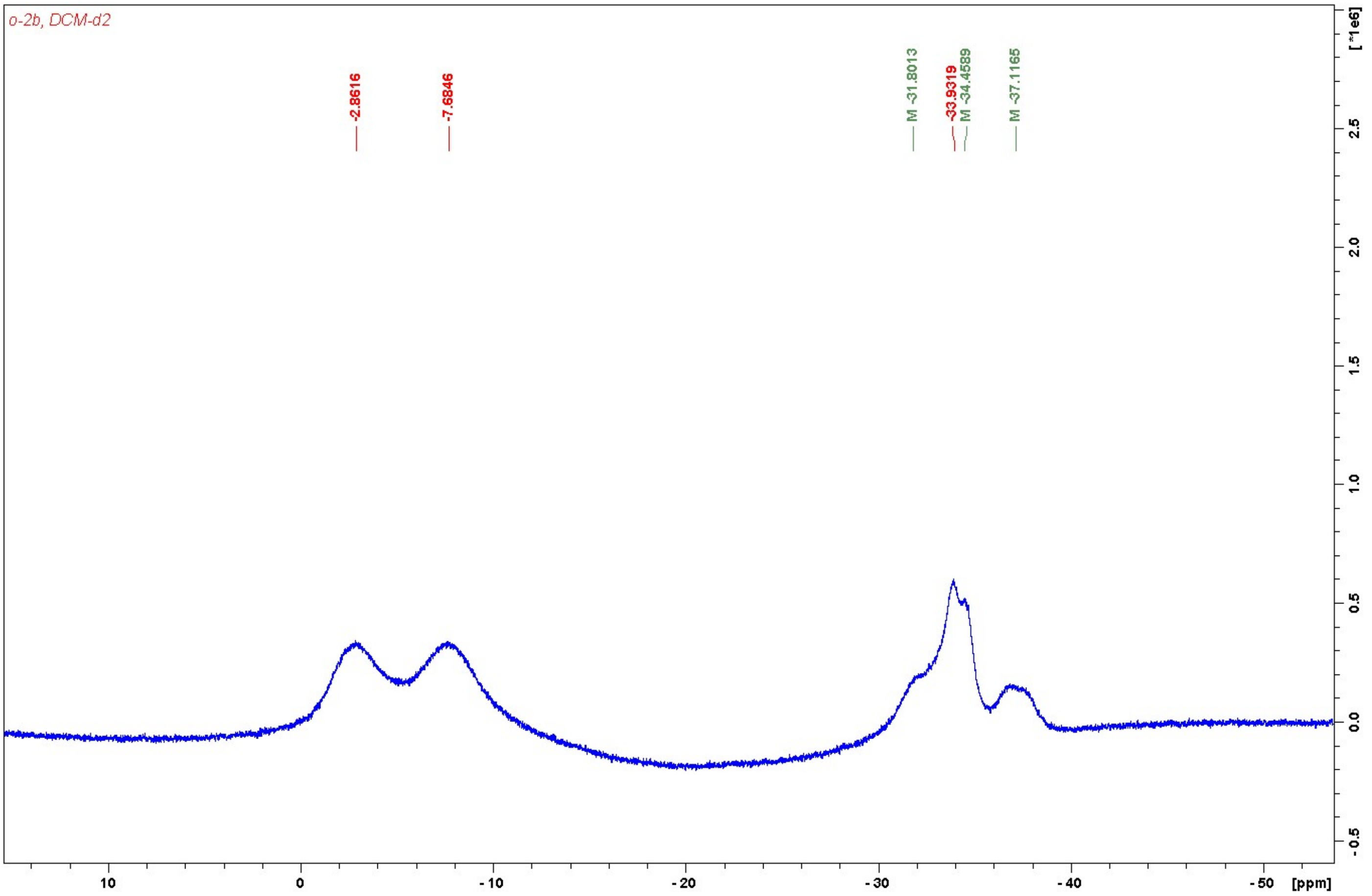

Supplementary Figure 49. ^{11}B NMR spectrum of *o*-2b.

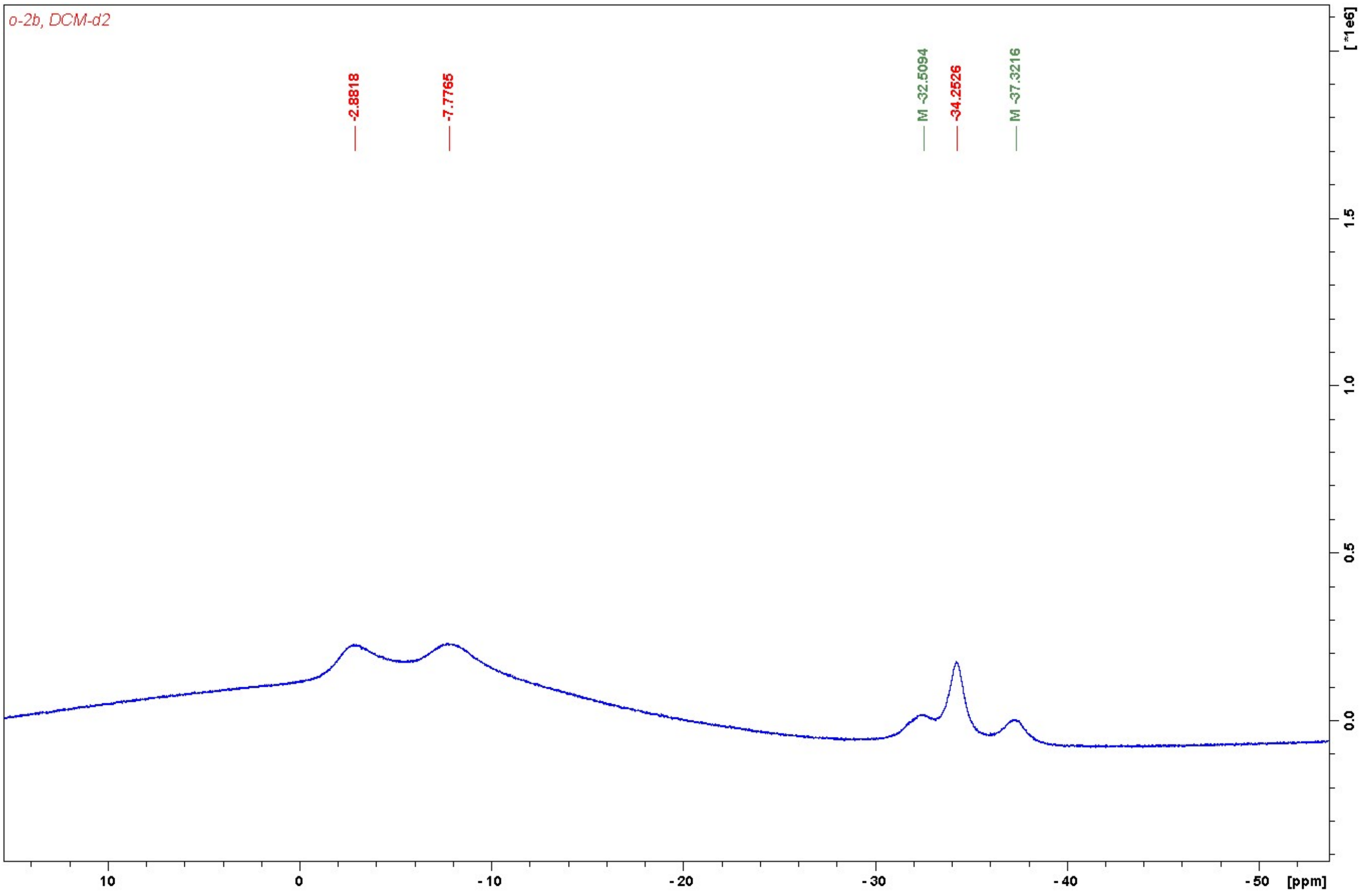

Supplementary Figure 50. ^{11}B{^{1}H} NMR spectrum of *o*-2b.

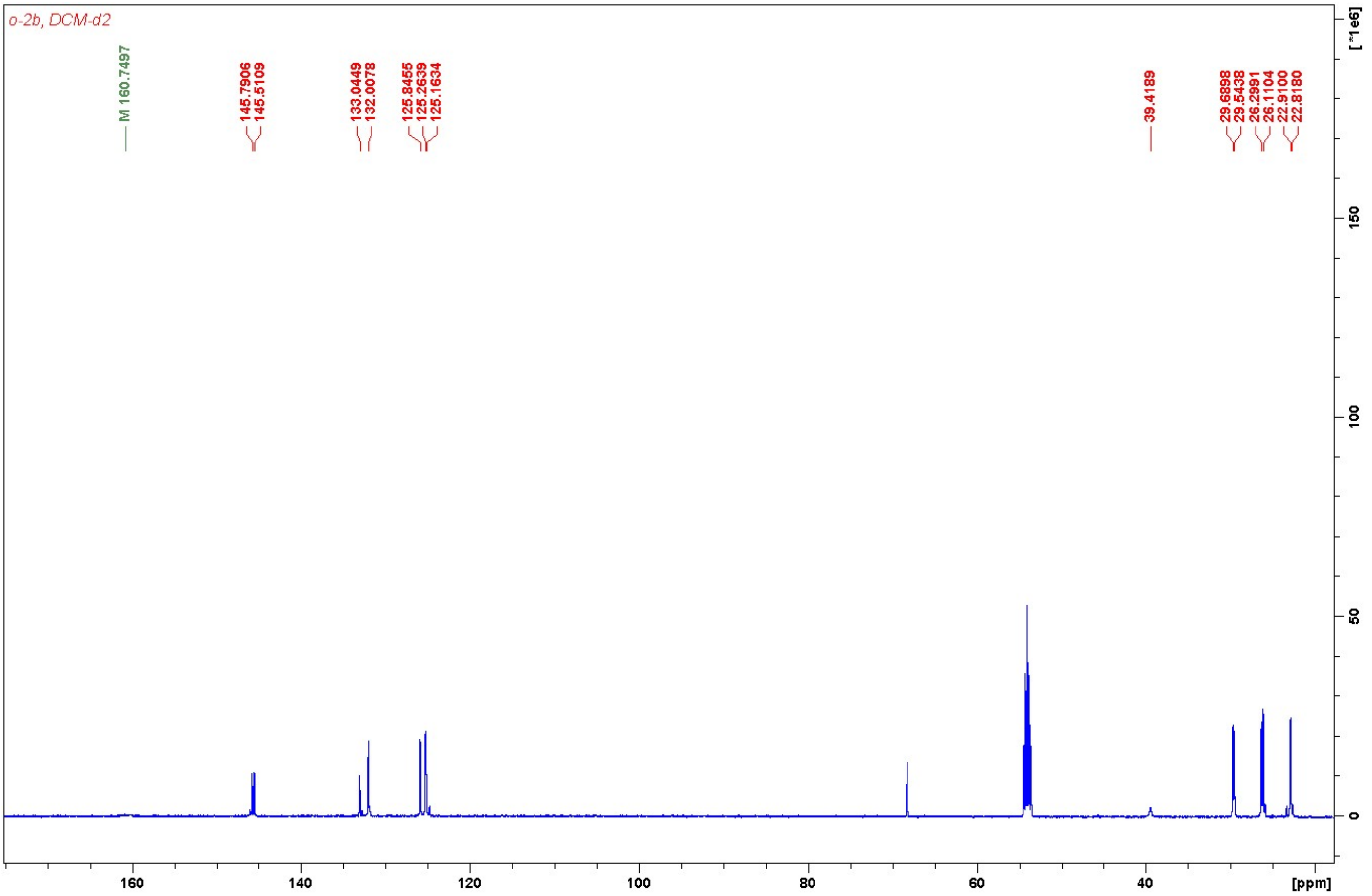

Supplementary Figure 51. $^{13}C\{^{1}H\}$ NMR spectrum of *o*-2b.

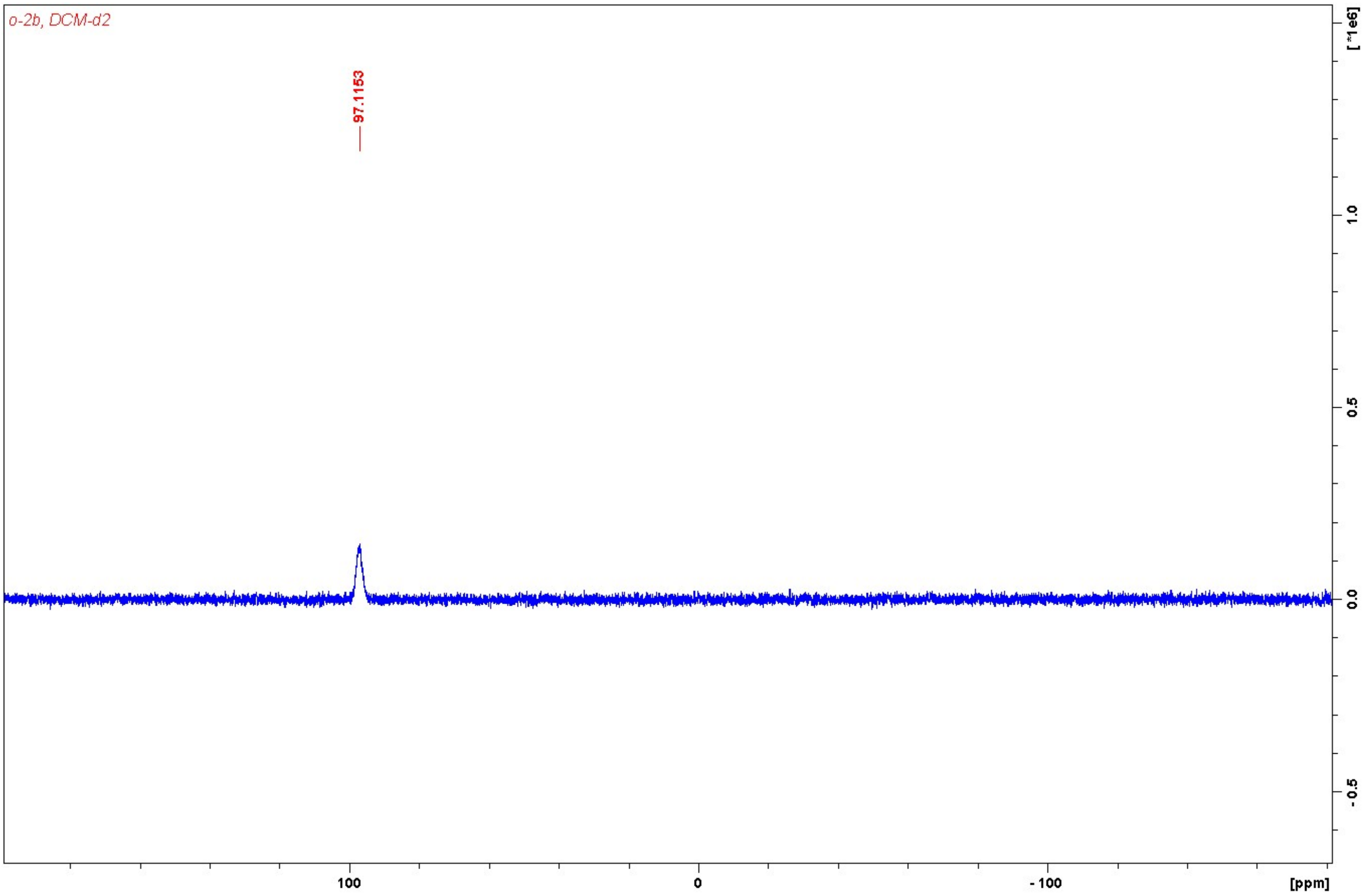

Supplementary Figure 52. ^{31}P NMR spectrum of *o*-2b.

Supplementary Table 9. Crystal data and structure refinement for *o*-2b.

Crystal data	
Chemical formula	$C_{58.50}H_{87}B_8Cl_8N_4P$
M_r	1247.37
Crystal system, space group	Triclinic, *P*-1
Temperature (K)	150
a, *b*, *c* (Å)	10.2456 (4), 15.3570 (6), 23.7234 (10)
α, β, γ (°)	72.2456 (19), 78.483 (2), 78.3490 (19)
V (Å^3)	3443.3 (2)
Z	2
Radiation type	Mo *K*α
μ (mm^{-1})	0.39
Crystal size (mm)	0.62 × 0.46 × 0.39
Data collection	
Diffractometer	Bruker D8 - Venture
Absorption correction	Multi-scan<br>*SADABS2016*/2 - Bruker AXS area detector scaling and absorption correction
T_{min}, T_{max}	0.764, 0.850
No. of measured, independent and observed [$I > 2\sigma(I)$] reflections	56766, 13409, 10644
R_{int}	0.048
$(\sin \theta/\lambda)_{max}$ (Å^{-1})	0.596
Refinement	
$R[F^2 > 2\sigma(F^2)]$, $wR(F^2)$, S	0.108, 0.287, 1.07
No. of reflections	13431
No. of parameters	802
No. of restraints	92
H-atom treatment	H atoms treated by a mixture of independent and constrained refinement
$\Delta\rho_{max}$, $\Delta\rho_{min}$ (e Å^{-3})	2.14, −1.89; located 0.69 and 1.19 Å from Cl7ʻ and Cl4, respectively

Supplementary Discussion

Position of the hydrogen bridge in *o*-1, *o*-2 and *o*-2a

As it was mentioned in the main text, in the structure of ***o*-1**, the carbene moiety is coordinated to a B-vertice without a hydrogen atom, which moved to the bridging position between boron atoms B8 and B9 (observed by ^{1}H NMR, see Supplementary Figure 53). Addition of the second equivalent of NHC yielded compound ***o*-2**, however, we were not able to detect a hydrogen bridge in its structure (the same works for ***m*-2** and ***p*-2**) in both NMR in solution (Supplementary Figure 53) or scXRD (***m*-2** and ***p*-2**) in the solid state. There is a possibility of the presence of an equilibrium between the isomer with two IPr→B-H vertices (Supplementary Figure 54A) and isomers with one or two hydrogen bridges (Supplementary Figure 54 B, C and D), which has not been proved experimentally by the NMR spectroscopy (not even in the range of -50 – +50 °C). Such an equilibrium could also explain the absence of any cross-peaks in ^{11}B-^{11}B COSY experiments of ***o*-2**, ***m*-2** and ***p*-2**. Interestingly, in ^{1}H NMR spectrum of ***o*-2a** is the hydrogen bridge again clearly visible (Supplementary Figure 53). Moreover, the scXRD method could not reveal such a phenomenon, since the isomer with hydrogen bridge always exhibits lower symmetry (C_1) than its analogue and thus lower tendency for crystallization. In addition, based on our long time expertize (more than 50 papers in top-tier journals during the last 15 years) on the field of (hetero)borane clusters crystallography, we know the H-bridge atom is easily visible in most of the cases as one of the first H-atoms during the *per partés* refinement on the Fourier difference electron density maps. In the structures of ***o*-1**, ***m*-2a** and ***o*-2a** the H-bridges are assigned according the maxima on the Fourier maps, while no maxima of such type is found on the maps of ***m*-2** and ***p*-2**.

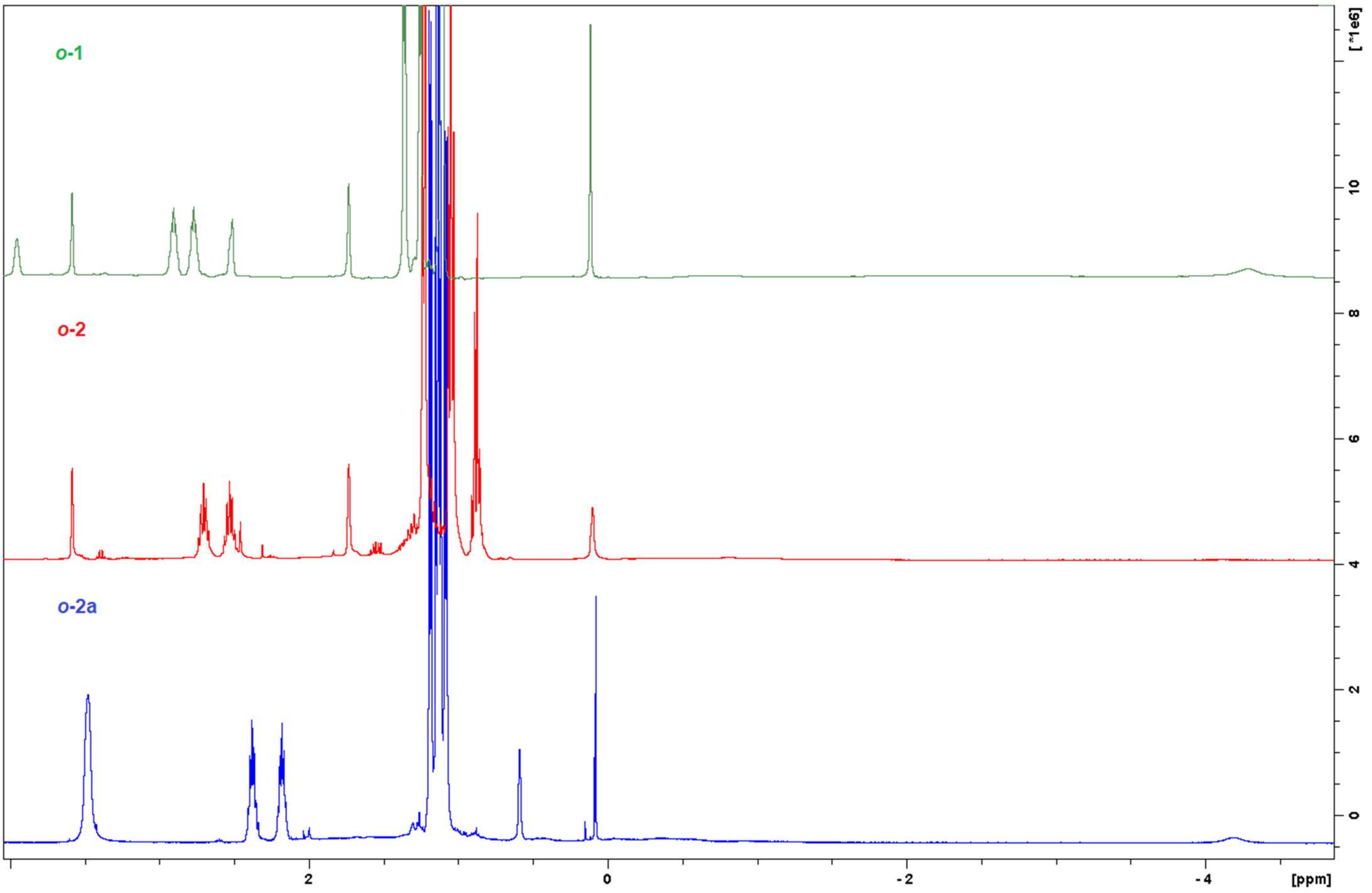

Supplementary Figure 53. ^{1}H NMR spectra of *o*-1, *o*-2 and *o*-2a.

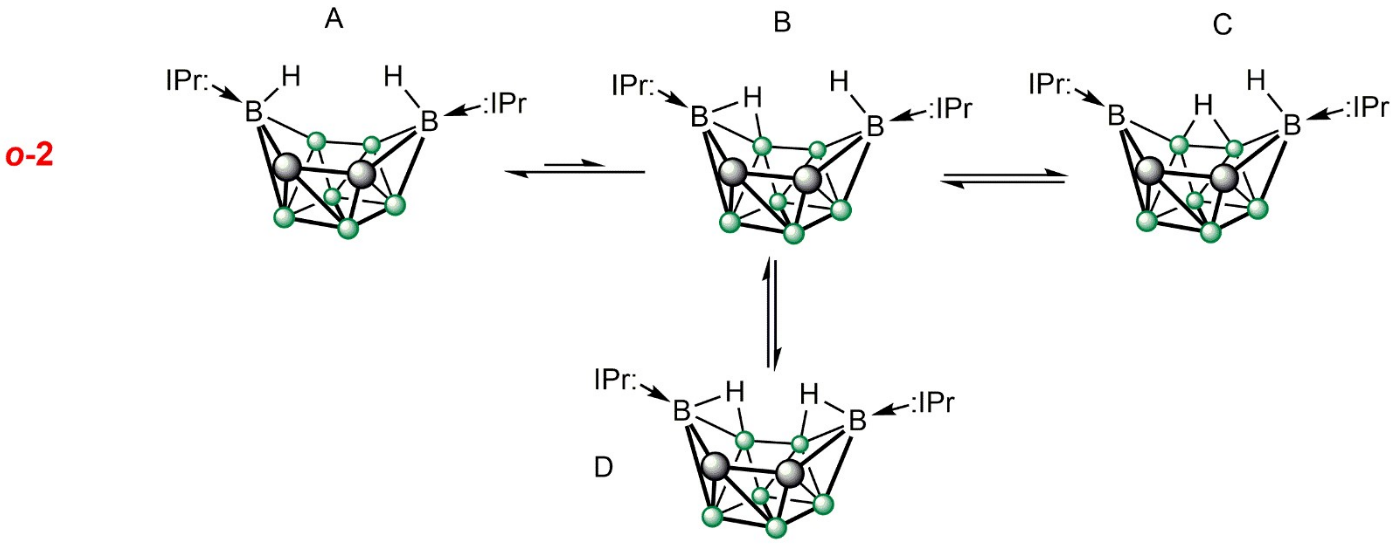

Supplementary Figure 54. Plausible equilibrium between suggested isomers of *o*-2.

Thermal decomposition of *o*-2, *m*-2 and *p*-2

As it has been already mentioned in the paper, ***o*-2** and ***m*-2** decompose at 100 °C in thf to the parent carborane ***p***. Both reactions were monitored by the help of ^{11}B NMR spectroscopy during one month (***o*-2** - Supplementary Figure 55 and - ***m*-2** Supplementary Figure 56). In both cases the first step is formation of an intermediate, which we were, unfortunately, not able to isolate and fully characterize. However, both intermediates exhibit very similar patterns in ^{11}B NMR spectra as the parent compounds. Moreover, none of the intermediates is ***p*-2**, which decomposes under the same conditions to ***p*** in one day. The decomposition of ***m*-2** is completed within one month while ***o*-2** shows ca 5 % conversion after the same time. Compared to the rearrangement of the parent carboranes ***o*** and ***m*** to ***p***, our method uses more gentle reaction conditions. However, considering the long reaction time and the presence of decomposition products of the carbene makes it inapplicable in bulk synthesis. The published method is much more simple, faster and high-yielding. Nevertheless, such kind of decomposition at lower temperatures has not been described yet and definitely deserves attention in the future studies.

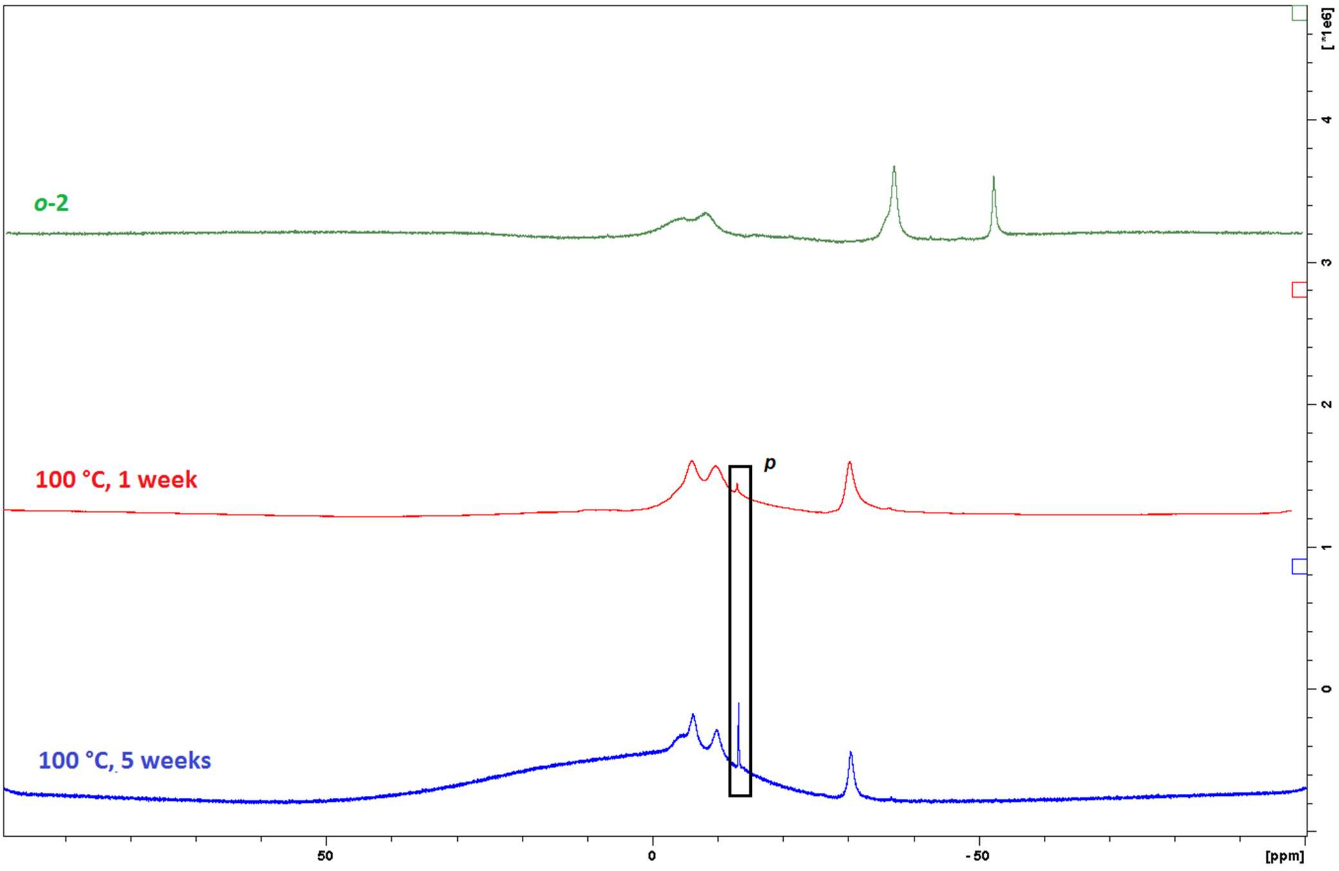

Supplementary Figure 55. **$^{11}B\{^{1}H\}$ NMR spectra of thermal decomposition of *o*-2.**

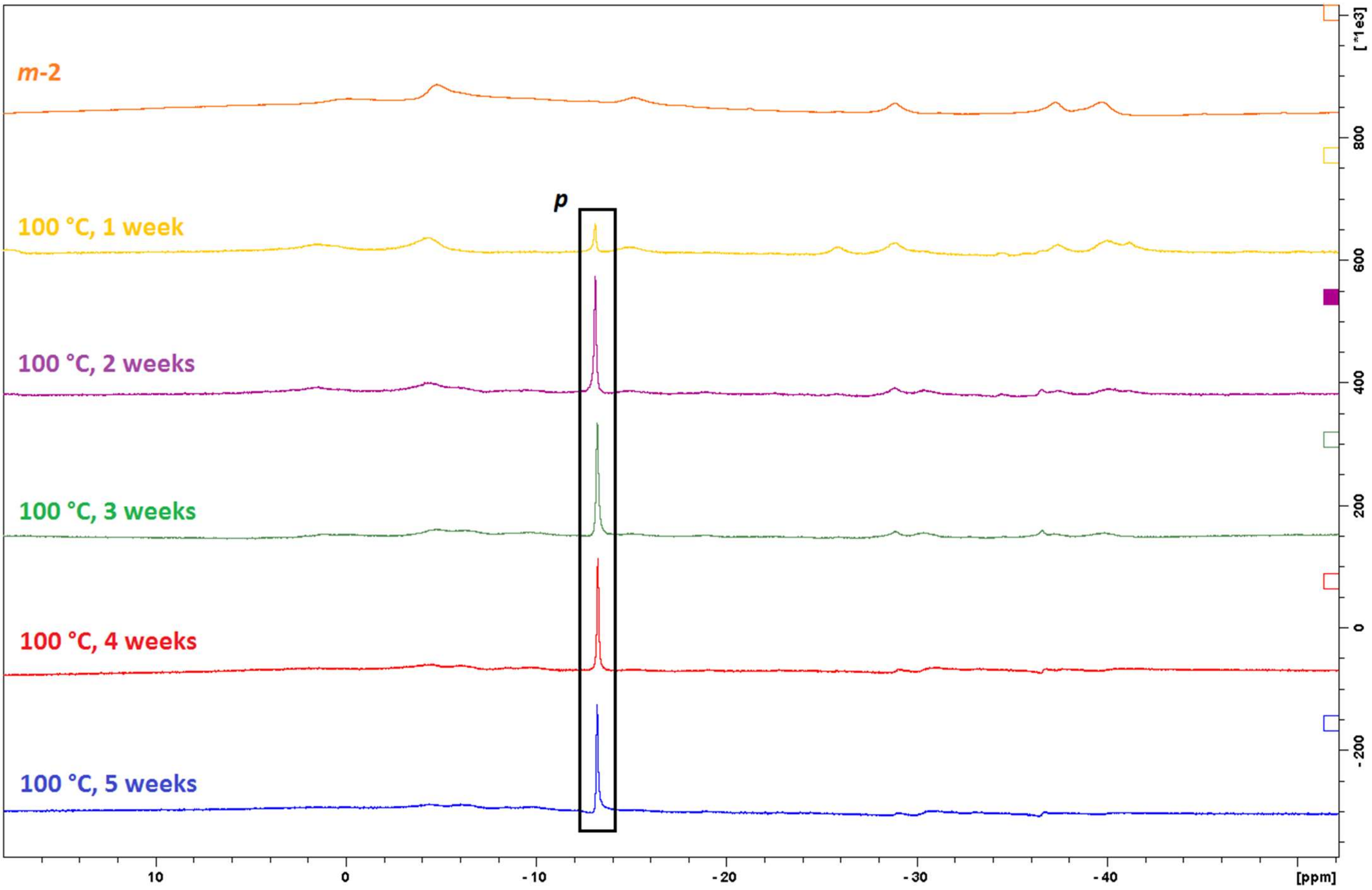

Supplementary Figure 56. **$^{11}B\{^{1}H\}$ NMR spectra of thermal decomposition of *m*-2.**

Synthesis of *o*-1a and *o*-1b

We proposed a simple mechanism of the formation of ***o*-1a** cluster (Supplementary Figure 57). First, the phosphorus atom is bound to atoms B8 and B9 (originally the position of the hydrogen bridge) and atom C6 (Supplementary Figure 57A). Subsequently, the bonds of the B2 atom with other boron atoms are cleaved (Supplementary Figure 57B) and B2 is turned around the C-C axis forming three new bonds (Supplementary Figure 57D). Finally, one last bond between the phosphorus atom and B11 (numbering of ***o*-1a**) is formed and a mirror analogue of ***o*-1a** is obtained. In the crystal structure, both mirror forms of ***o*-1a** are present in the solid state according to the X-Ray analysis. Both can be obtained by the same mechanism from the corresponding isomer of ***o*-1**, which presence was not confirmed not excluded in the solid state by the X-Ray analysis, however, since the difference between both forms is only the position of hydrogen bridge in two, chemically equivalent positions, it is highly possible, that both isomers are present in the solution.

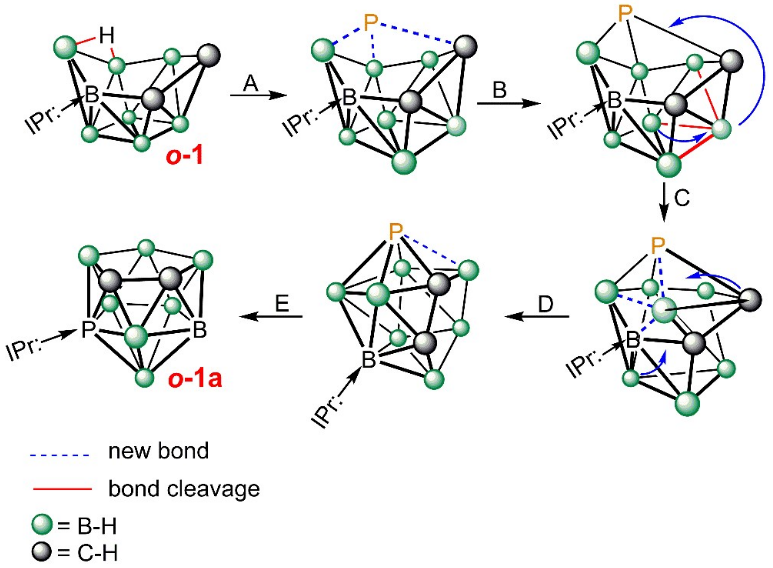

Supplementary Figure 57. Plausible mechanism of the formation of *o*-1a.

We have proposed a plausible mechanism for the rearrangement of the borane cage (Figure 4), which consists of oxidation as the initial step (**A**), followed by a simple cleavage of three skeletal bonds of atom B1 (**B**) with its subsequent turn around the C-C (**C**) bond, and finally the closure of the borane cage.

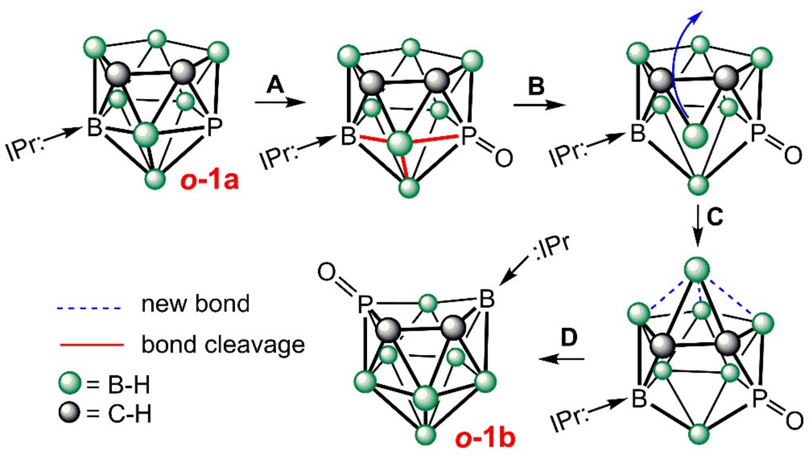

Supplementary Figure 58. A plausible mechanism for the atom rearrangement after the oxidation of *o*-1a to *o*-1b.

Supramolecular architecture of *o*-2a, *m*-2a and *p*-2a

Supplementary Figures 59 – S61 show the hydrogen bonding or short contacts between negative and positive parts in ***o*-2a**, ***m*-2a** and ***p*-2a**.

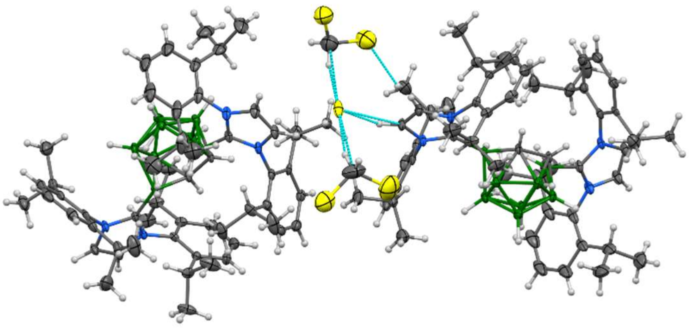

Supplementary Figure 59. **Supramolecular architecture of *o*-2a.** Colour coding: carbon-grey, hydrogen-white, boron-green, nitrogen-blue and chlorine-yellow.

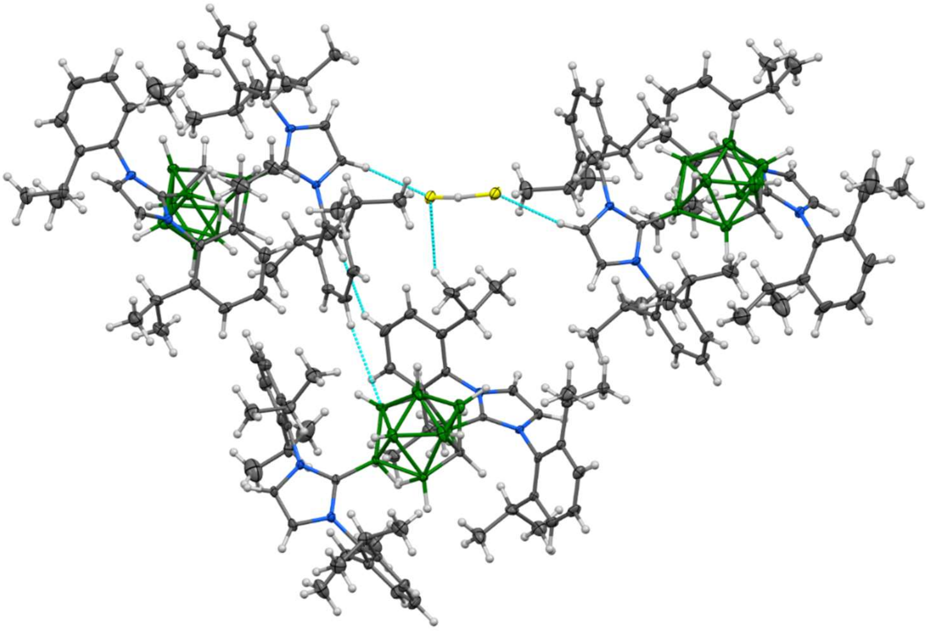

Supplementary Figure 60. Supramolecular architecture of *m*-2a. Colour coding: carbon-grey, hydrogen-white, boron-green, nitrogen-blue and chlorine-yellow.

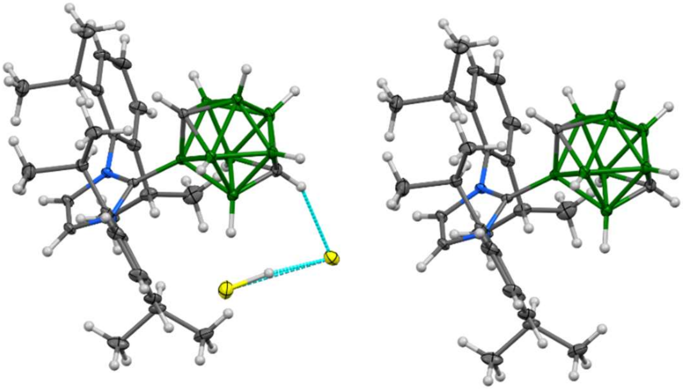

Supplementary Figure 61. Supramolecular architecture of *p*-2a. Colour coding: carbon-grey, hydrogen-white, boron-green, nitrogen-blue and chlorine-yellow.

Supplementary Computational Data

All the calculations (except GIAO-MP2, see bellow) were performed with the Gaussian 16 program.[6] The geometries of the compounds (***o***, ***m***, ***p***, ***o*-1**, ***m*-1**, ***p*-1**, ***o*-2**, ***m*-2**, ***p*-2**) were fully optimized at B3LYP-D3/cc-pVDZ level of theory[7] without any simplifications. The structures of complexes obtained by X-ray diffraction were used as the initial data. The polarizable continuum model (PCM)[8] was employed for the solvation effects (diethyl ether). All the structures are minima on the potential energy surface, as confirmed by the frequency calculations at the same level of theory and transition states by only one imaginary frequency. The topological analysis of the theoretical function ρ(*r*) was performed using the AIMALL program package.[9] Thus structures (***o*-1**, ***o*-2**, ***o*-2a**, ***o*-2b**, ***m*-2**, ***m*-2a**, ***p*-2**, ***p*-2a**) were fully optimized at B3LYP/cc-pVTZ level of theory. The numbering of atoms corresponds to the numbering for XRD.

NMR Shifts

Magnetic shielding was calculated using the GIAO-MP2 method incorporated into Gaussian 16[10] utilizing the IGLO-II basis with the MP2/TZVP geometries and frozen core electrons. In order to reduce the GIAO-MP2 jobs to manageable dimensions, we approximated the Dipp groups with hydrogen atoms. Earlier work[11] provided a very good back-up for such an assumption. The best-fit assignments reveal very good accords between the GIAO-MP2 and experimental values (Supplementary Table 10). However, there are a few boron vertices that are apparently influenced by the 'computational' deshielding by the NHC moieties. Therefore, the computed ^{11}B chemical shifts resonate at a little bit higher frequencies (downfield) than the measured values show.

Supplementary Table 10. Computed and experimental ^{11}B NMR shifts [ppm].

Compound		Vertex										
		1	2	3	4	5	6	7	8	9	10	11
***o*-1**	Comp.	-2.4	-31.1	-12.2	-26.2	C	C	11.2	2.0	13.7	-19.3	X
	Exp.	-1.2	-28.0	-14.0	-32.1			10.3	-3.4	18.6	-15.8	
***o*-1a**	Comp.	-43.8	P	-25.1	-14.9	-19.1	-10.5	C	C	-0.8	1.7	-19.3
	Exp.	-39.6		-29.5	-17.3	-12.0	-10.4			-8.0	-5.9	-24.9
***o*-1b**	Comp.	-45.4	-17.0	-12.0	-17.7	-31.3	-14.7	P	C	C	-21.0	-20.1
	Exp.	-45.9	-17.4	-13.5	-17.4	-32.1	-14.4				-24.3	-20.1
***o*-2**	Comp.	-33.2	-2.5	-50.0	-2.5	C	-35.6	-3.3	-3.3	-35.6	C	X
	Exp.	-37.1	-4.8	-52.3	-4.8		-37.1	-8.2	-8.2	-37.1		
***o*-2a**	Comp.	-22.2	-7.1	-53.5	-7.1	C	-31.2	-1.4	-1.4	-31.2	C	X
	Exp.	-22.6	-7.3	-54.7	-7.3		-31.7	-4.7	-4.7	-31.7		
***o*-2b**	Comp.	-34.0	-2.8	-34.6	-2.8	C	-33.5	-4.9	-4.9	-33.5	C	P
	Exp.	-32.5	-2.9	-37.3	-2.9		-34.3	-7.8	-7.8	-34.3		
***m*-2**	Comp.	-39.6	2.5	-39.6	-7.4	C	-30.6	C	-4.8	-39.3	-4.8	X
	Exp.	-39.8	0.3	-39.8	-15.1		-28.9		-4.8	-37.3	-4.8	
***m*-2a**	Comp.	-35.6	-3.3	-35.6	-31.6	C	-22.2	C	-5.4	-37.8	-5.4	X
	Exp.	-36.0	-1.5	-36.0	-33.7		-24.9		-5.0	-37.5	-5.0	
***p*-2**	Comp.	-43.6	-3.1	-43.6	-3.1	C	-37.4	-10.3	C	-37.4	-10.3	X
	Exp.	-44.7	-7.0	-44.7	-7.0		-36.4	-9.9		-36.4	-9.9	
***p*-2a**	Comp.	C	-13.9	-11.0	-9.6	-9.6	-12.6	-7.9	-9.6	-14.9	C	X
	Exp.		-13.6	-11.6	-11.6	-11.6	-11.6	-11.6	-11.6	-13.6		

Reactions of ***o***, ***m*** and ***p*** with :IPr

To understand the differences in the reactions of ***o***, ***m*** and ***p*** with :IPr, the geometries of the ground states were optimized, and then the corresponding free energies were calculated (Supplementary Figure 62). The ΔGs calculated in order to shed more light on the reactivity of parent carboranes are consistent with the experiment. The low energy of the first reaction with ***o*** (−27.19 kcal/mol) and the higher ΔG of the second one (-13.36 kcal/mol) are explained both by the simultaneous presence of compounds ***o*-1** and ***o*-2** in the reaction mixture and by the willingness of ***o*** to react. Contrastingly, the high value of the ΔG of the reaction of ***p*** with the first :IPr (8.98 kcal/mol) and the negative ΔG value for the subsequent :IPr addition (−32.57 kcal/mol) explain the reluctance of ***p*** to react and the impossibility to detect ***p*-1**. The reactions of ***m*** exhibit highly negative values of both ΔGs (-24.10 and -17.2 kcal/mol), which also confirm reaction progress with no intermediate.

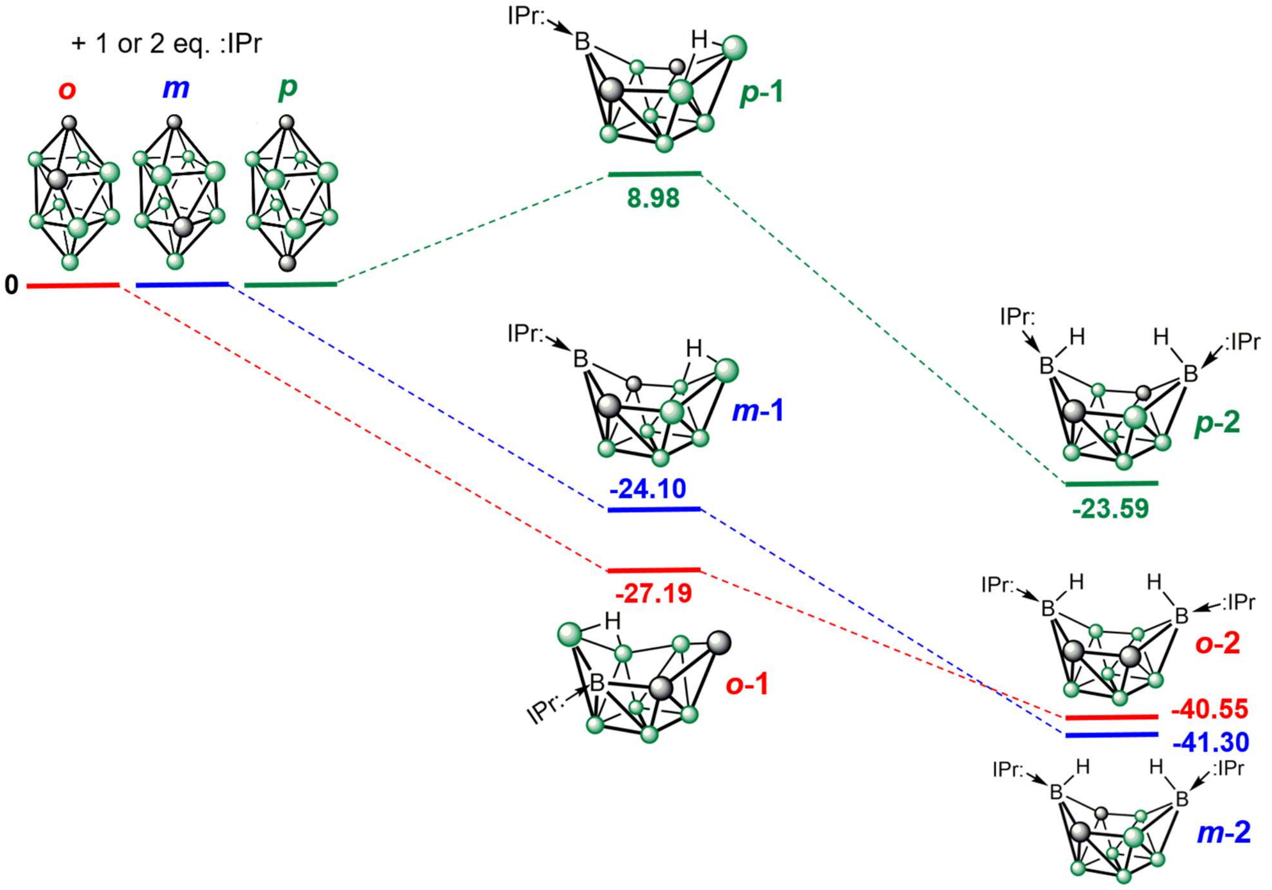

Supplementary Figure 62. **The DFT-estimated (B3LYP-D3/cc-pVDZ theory level) relative Gibbs free energy comparison for reactants and products (kcal/mol).** (Notes: the lines connecting the red, green and blue energy levels are shown only as a guide to the reader's eye. The relative Gibbs free energies of parent carboranes ***o***, ***m*** and ***p*** are obviously not 0, but 38.3, 20.2 and 0 kcal/mol for ***o***, ***m*** and ***p*** –the comparative approach is used here in order to illustrate differences between reactants and products in all series more clearly).

QTAIM

Within the framework of R. Bader's theory "Atoms in Molecules", the atomic charges in compounds ***o*-1**, ***o*-2**, ***m*-2**, ***p*-2** were calculated (Supplementary Table 11). In the series of bis-carbene derivatives, the smallest negative charge on carbon atoms C5, C10 (-0.996, -1.010 e) is observed in ***o*-2**, while C5, C7 (-1.656, -1.656 e) and C5, C8 (-1.616, -1.615 e) in ***m*-2** and ***p*-2**, respectively, have similar charges. At the same time, in ***m*-2**, the value of the positive charge on the B6 atom (1.740 e), which is associated with C5, C7 and C11 (carbene), significantly increases. Due to this redistribution of the charge, the second carbene (C38) becomes partially

negative (-0.061 e). As for the ionic compounds ***o*-2a**, ***o*-2b**, ***m*-2a**, ***p*-2a** (Supplementary Table 12), the positive charge of $B_8C_2H_{9/11}$/$B_8C_2H_{10}PCl_2$ fragments is delocalized through the whole cage with only small changes when compared to the parent compounds.

Supplementary Table 11. B3LYP/cc- pVTZ AIM atomic charges (a.u.) in *o*-1, *o*-2, *m*-2, *p*-2.

Charges in B_8C_2 cluster:									
***o*-1**									
B1	B2	B3	B4	B5	C6	C7	B8	B9	B10
0.506	1.085	0.760	0.636	1.302	-1.049	-0.924	0.796	0.841	0.838
***o*-2**									
B1	B2	B3	B4	C5	B6	B7	B8	B9	C10
0.965	0.501	0.742	0.509	-0.996	1.356	0.680	0.665	1.385	-1.010
***m*-2**									
B1	B2	B3	B4	C5	B6	C7	B8	B9	B10
0.857	1.169	0.857	0.562	-1.656	1.740	-1.656	1.086	0.750	1.086
***p*-2**									
B1	B2	B3	B4	C5	B6	B7	C8	B9	B10
0.858	0.723	0.858	0.723	-1.616	1.305	1.124	-1.615	1.306	1.124

Total charge in fragments:			
	$B_8C_2H_{10}$	Carbene (C11)	Carbene (C38)
***o*-1**	-0.029	0.032	–
***o*-2**	-0.044	0.041	0.006
***m*-2**	-0.023	0.088	-0.061
***p*-2**	-0.030	0.016	0.017

Supplementary Table 12. B3LYP/cc-pVTZ AIM atomic charges (a.u.) in *o*-2a, *o*-2b, *m*-2a, *p*-2a.

Charges in B_8C_2/$B_8C_2PCl_2$ cluster:									
***o*-2a**									
B1	B2	B3	B4	C5	B6	B7	B8	B9	C10
1.049	0.769	0.634	0.766	-1.067	1.477	0.898	0.907	1.477	-1.055
***o*-2b**									
B1	B2	B3	B4	C5	B6	B7	B8	B9	C10
1.074	0.725	0.712	0.748	-1.053	1.444	0.847	0.865	1.487	-1.047
P11	Cl1	Cl2							
0.390	-0.503	-0.530							
***m*-2a**									
B1	B2	B3	B4	C5	B6	C7	B8	B9	B10
0.918	1.324	0.918	0.479	-1.720	1.772	-1.733	1.343	1.178	1.365
***p*-2a**									
C1	B2	B3	B4	B5	B6	B7	B8	B9	C10
-2.164	1.037	1.106	1.120	1.106	1.105	1.095	1.095	1.105	-2.131

Total charges in fragments:				
	$B_8C_2H_{9/11}$/$B_8C_2H_{10}PCl_2$	Carbene (C8/C11)	Carbene (C38)	Cl^-/HCl_2^-
***o*-2a**	0.692	0.103	0.071	-0.865
***o*-2b**	0.659	0.089	0.123	-0.865
***m*-2a**	0.631	0.140	0.141	-0.911
***p*-2a**	0.713	0.170	–	-0.884

The atomic charges also shed more light on the diversity in the reactions of ***o*-2**, ***m*-2** and ***p*-2** with hydrogen chloride. The elimination of the dihydrogen in the case of ***p*-2** is most probably caused by the impossibility to form stable compound containing a hydrogen bridge. In ***o*-2** and ***m*-2**, the hydrogen bridge is placed between the least positively charged B-atoms in the upper rim of the carborane cage (0.68 and 0.665 e for ***o*-2**; 1.086 and 0.75 e for ***m*-2**). On the contrary, ***p*-2** does not have such low atomic charges in the upper rim (1.124-1.306 e). Moreover, the distant position of the C-atoms also lowers the number of plausible locations for the hydrogen bridge. Therefore, the ***p*-2**H^+ adduct releases imidazolium and the newly formed ***p*-1** (which is not thermodynamically very stable, see above) reacts with another equivalent of hydrogen chloride releasing dihydrogen and closing the carborane cage to ***p*-2a**.

An analysis of the distribution the Laplacian of the electron density ($\nabla^2\rho(r)$) of ***o*-2b** in the B6-P11-B8 plane demonstrates the regions of electron density (ED) concentration between those atoms. Taking into account the other topological parameters at these bond critical points (BCP, Supplementary Table 13, Supplementary Figure 63), such interactions can be characterised to as shared interactions.

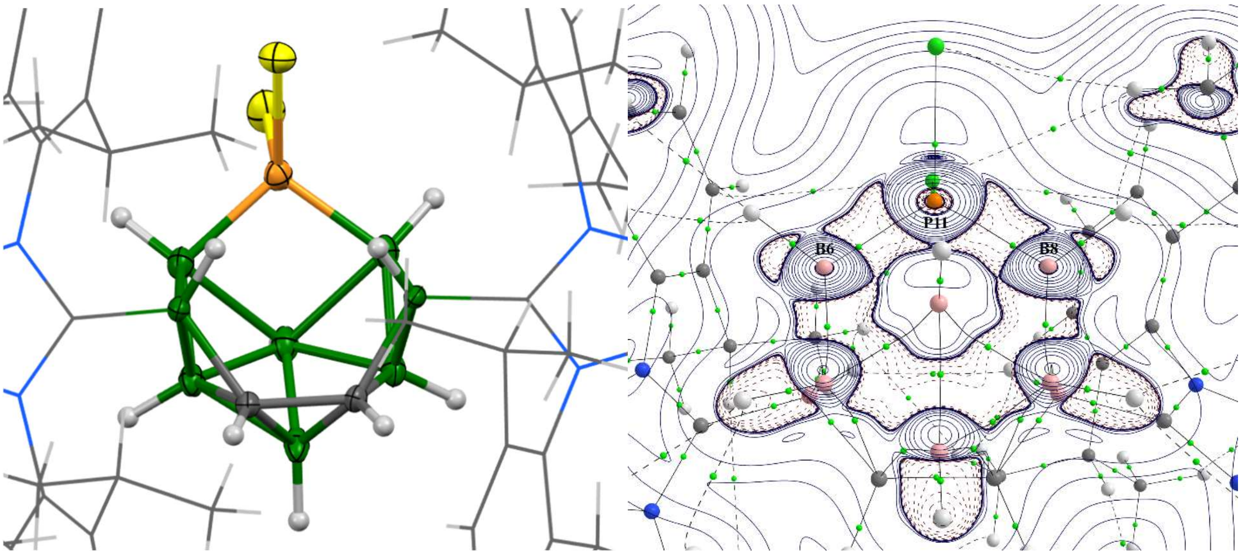

Supplementary Figure 63. Laplacian plot of the electron density distribution (right) in B6-P11-B8 plane of *o*-2b. Red dashed lines correspond to negative values of $\nabla^2\rho(r)$, solid blue – positive. Only BCP(3,-1) are presented in green for clarity.

Supplementary Table 13. **Topological properties of BCPs for *o*-2b in a.u.**[a]

Bond	$\rho(r_{cp})$	$\nabla^2\rho(r_{cp})$	$G(r_{cp})$	$V(r_{cp})$	$H(r_{cp})$
B6-P11	0.142	-0.228	0.077	-0.210	-0.134
B8-P11	0.144	-0.255	0.070	-0.205	-0.134

[a]$\rho(r_{cp})$ - the electron density, $\nabla^2\rho(r_{cp})$ - the Laplacian function of the electron density, $G(r_{cp})$ - the kinetic electron energy density, $V(r_{cp})$ - the potential electron energy density, $H(r_{cp})$ - the total electron energy density.

ESP

In order to understand the charge distribution on the molecular surfaces of cationic species, the electrostatic potential (ESP) molecular surfaces of the ***o*-2a**, ***o*-2b**, ***m*-2a** and ***p-2a*** were computed on the 0.001 a.u. molecular surfaces at the B3LYP/cc-pTZVP level using the Gaussian 16[6] and Molekel4.3 programs.[12,13] The ESP color range is in kcal/mol. The negative surface is always located on Cl^- and HCl_2^- parts, respectively. The positive surface is always found on whole carborane moiety, except of one BH vertex next to the carbene(s). The areas with the most positive ESP, which interact with counter-anion, are the CH vertexes of carborane cages and CH=CH fragment of the NHCs.

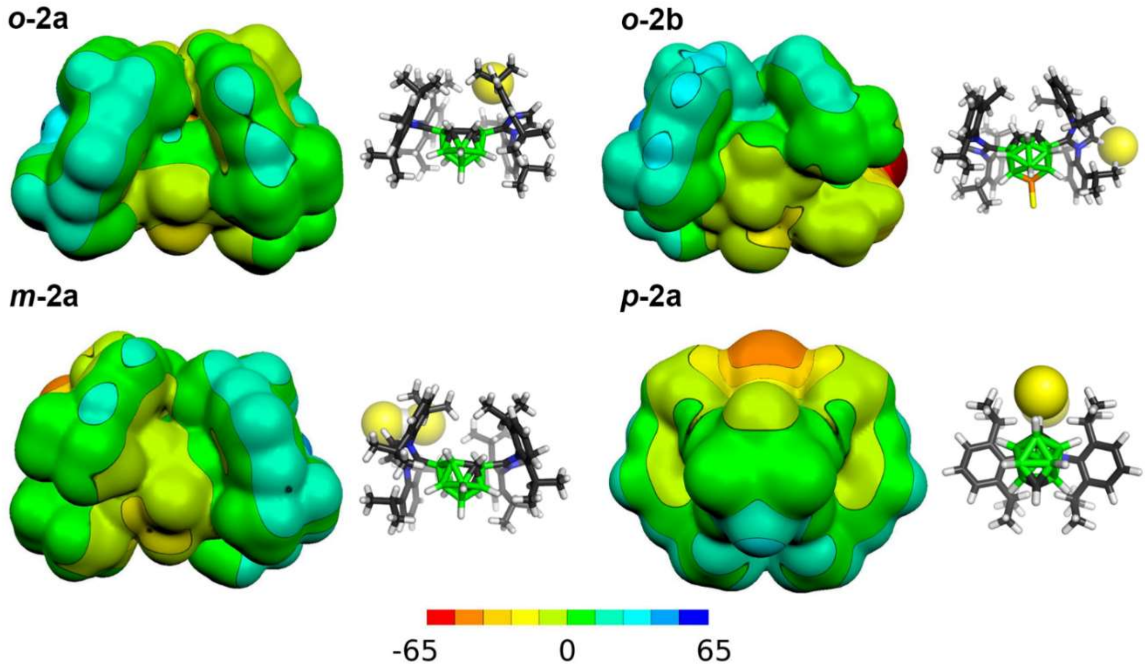

Supplementary Figure 64: Computed (B3LYP/cc-pVTZ) electrostatic potential (ESP) surface for *o*-2a, *o*-2b, *m*-2a and *p*-2a. The color range of the ESP in kcal/mol.

IBO

The intrinsic atomic/bond orbital (IAO/IBO) method,[14] which works well for heteroboranes[15] was run at B3LYP/def2-TZVPP//MP2/TZVP. The computed IBO orbitals for classify the bonding patterns in ***o*-2b** as follows: 2e-2c B-P bond, 2e-2c B-C(NHC) bond, 2e-2c P-Cl, 2e-3c B7-B-B bond, 2e-4c B1-B2-B3-B4, 2e-2c C5-C10 bond, 3 π orbitals emerge in NHC. This system might be viewed as a 'staggered' P^+ system that is stabilized with the cage. The P-Cl bond is more polarized than the B-C(NHC). P atom is bonded classically (2e-2c) both to chlorine and boron atoms. The B7-B8 nearest-neighbor separation is extremely long, i.e. 2.91 Å (MP2/TZVP and X-ray). The bond length of P-B7(or B8) amounts to 95 % of the sum of the van der Waals radii.

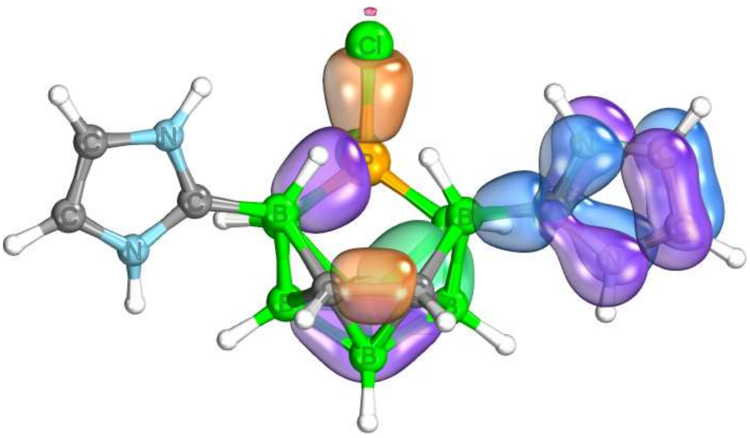

Supplementary Figure 65: The most decisive IBO orbitals for *o*-2b.

Supplementary References

1. Tok, O. L. et al. Click dehydrogenation of carbon-substituted *nido*-5,6-C2B8H12 carboranes: a general route to *closo*-1,2-C2B8H10 Derivatives. *Inorg. Chem.* **55**, 8839−8843 (2016).

2. Bakardjiev, M., Štíbr, B., Holub, J., Padělková, Z. & Růžička, A. Simple synthesis, halogenation, and rearrangement of *closo*-1,6-C2B8H10. *Organometallics* **34**, 450−454 (2015).

3. Holub, J., Jelínek, T. & Janoušek, Z. New multigram-scale preparation of 1,10-dicarba-*closo*-decaborane. *Collect. Czech. Chem. Commun.* **67**, 949−952 (2002).

4. Benhamou L., Chardon, E., Lavigne, G., Bellemin-Laponnaz, S. & César, V. Synthetic Routes to N-Heterocyclic Carbene Precursors. *Chem. Rev.* **111**, 2705–2733 (2011).

5. Sheldrick, G. M. *SHELXT* - Integrated space-group and crystal-structure determination. *Acta Cryst.* **A71**, 3−8 (2015).

6. Frisch, M. J. et al. Gaussian 16, Revision A.03 (Gaussian, Inc., Wallingford CT, USA, 2016)

7. Becke, A. D. Density-functional thermochemistry. III. The role of exact Exchange. *J. Chem. Phys.* **98**, 5648−5652 (1993); Grimme, S., Antony, J., Ehrlich, S. & Krieg, H. A consistent and accurate *ab initio* parametrization of density functional dispersion correction (DFT-D) for the 94 elements H-Pu. *J. Chem. Phys.* **132**, 154104 (2010). Dunning, T. H. Jr. Gaussian basis sets for use in correlated molecular calculations. I. The atoms boron through neon and hydrogen. *J. Chem. Phys.* **90**, 1007−1023 (1989).

8. Tomasi, J., Mennucci, B. & Cammi, R. Quantum mechanical continuum solvation models. *Chem. Rev.* **105**, 2999−3093 (2005).

9. Keith, T. A. AIMAll, Version 19.10.12 (TK Gristmill Software, Overland Park KS, USA, 2019)

10. Frisch, M. J. et al. Gaussian 16, Revision C.01 (Gaussian, Inc., Wallingford CT, USA, 2016)

11. Vrána, J. et al. Investigation of thiaborane *closo*–*nido* conversion pathways promoted by *N*-heterocyclic carbenes. *Inorg. Chem*. **58**, 2471−2482 (2019).

12. Flűkiger, P.; Lűthi, H.P.; Portmann, S.; Weber, J. MOLEKEL 4.3; Swiss Center for Scientific Computing: Manno, Switzerland, 2000.

13. Portmann, S.; Lűthi, H. P. MOLEKEL: An Interactive Molecular Graphic Tool. CHIMIA. *Int. J. Chem*. **54**, 766–770 (2000).

14. Knizia, G. Intrinsic Atomic Orbitals: an unbiased bridge between quantum theory and chemical concepts. *J. Chem. Theory Comput*. **9**, 4834–4343 (2013).

15. Melichar, P., Hnyk, D., & Fanfrlík, J. A systematic examination of classical and multi-center bonding in heteroborane clusters. *Phys. Chem. Chem. Phys.* **20**, 4666–4675 (2018).
